# Fundamentals and Perspectives on Materials for Bifunctional Electrocatalysis

**DOI:** 10.1002/advs.202509902

**Published:** 2025-08-21

**Authors:** Iqra Fareed, Muhammad Danish Khan, Mashal Firdous, Tahmina Maqsood, Masood ul Hassan Farooq, Muhammad Tahir, Faheem K. Butt, Ji‐Jun Zou, Shangfeng Du

**Affiliations:** ^1^ Laboratory of Eco‐Materials and Sustainable Technology (LEMST) Natural Sciences and Humanities Department New Campus, UET Lahore 54890 Pakistan; ^2^ Department of Physics University of Engineering and Technology Lahore 54890 Pakistan; ^3^ Department of Physics Division of Science and Technology University of Education Lahore 54770 Pakistan; ^4^ School of Chemical Engineering and Technology Tianjin University Tianjin 300072 China; ^5^ School of Chemical Engineering University of Birmingham Birmingham B15 2TT UK

**Keywords:** bifunctional electrocatalyst, electrocatalysis, hydrogen evolution reaction (HER), nanocomposite, oxygen evolution reaction (OER)

## Abstract

Electrocatalysts are the core material for water electrolysis, as a promising technology for green hydrogen production coupled with renewable energy resources. Among various electrocatalyst development strategies, bifunctional electrocatalysts have attracted fast‐growing efforts, due to their abilities for catalyzing both hydrogen evolution reaction (HER) and oxygen evolution reaction (OER) involved in the electrocatalysis process for reducing the cost in fabrication, operation, and systems. In this study, a comprehensive review of the latest advancements and ongoing research for the bifunctional electrocatalysts uniquely based on their families is presented, aiming to gain deep insights into the field and provide an outlook for the direction of future developments. First, an in‐depth discussion is conducted on the mechanisms for both reactions involved in the electrocatalysis process, followed by examining their critical affecting factors. Then, as the heart of this work, a wide range of bifunctional electrocatalyst families are reviewed, including metal–organic frameworks, metal oxide‐based catalysts, hydrides and hydroxides, carbon‐based materials, phosphides, and chalcogenides. The synergistic effects are also explored for combining different materials through the formation of nanocomposites and heterojunctions. Finally, the challenges encountered with current technologies and perspectives are explored to provide practical and scalable electrocatalyst solutions for water electrolysis.

## Graphical abstract

This review presents a comparative evaluation of key catalyst families for HER and OER, including MOFs, metal oxides, hydroxides/hydrides, carbon‐based materials, phosphides, and chalcogenides. Their electrochemical activity, stability, and commercialization potential are critically analyzed, with design strategies for hybrid catalysts also discussed.

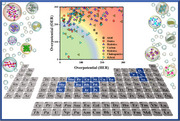

## 1 Introduction

The energy demand with an expanding population and rising living standards continues to grow. According to the International Energy Agency (IEA), global energy demand is expected to reach 30 TW and 46 TW in 2050 and 2100, respectively.^[^ ^1^ ^]^ The increasing energy requirements and limited supply of fossil fuels, along with environmental impacts, necessitate research for novel clean energy production strategies. Energy production via renewable sources has thus been identified as the most affordable and sustainable technology in this regard, such as solar energy and wind energy. By 2020, renewable energy sources will have accounted for ≈29% of the global electricity production, which is anticipated to increase to ≈45% in 2040.^[^ ^2^ ^]^ The United Nations climate change conference, COP26, has presented a top ambition to attain net‐zero carbon emissions by 2050. Renewable energy sources could play a crucial role in reducing carbon emissions, offering sustainable alternatives to conventional fossil fuels to meet the targets. However, their intermittent nature poses challenges for a consistent energy supply, necessitating efficient energy storage solutions. Hydrogen emerges as a highly promising candidate for this purpose due to its high energy density and environmental friendliness, enabling it to bridge the gap between energy generation and storage in a sustainable energy ecosystem.

**Figure 1:**
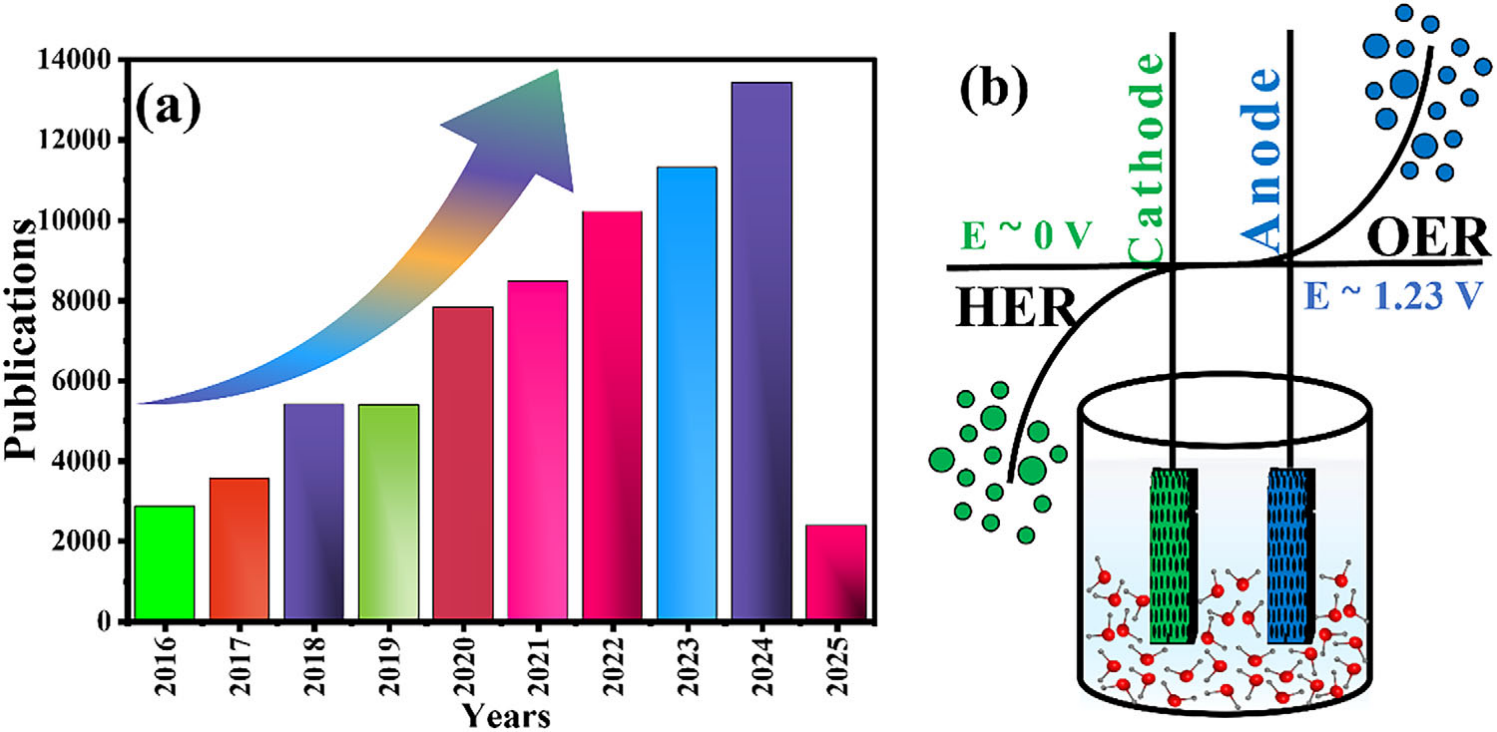
a) Publications per year between 2015–2025 on “HER” and “OER”, data acquired from dimensions.ai (09‐02‐2025), b) Electrocatalytic water‐splitting reaction displaying HER at the cathode and OER at the anode.

Hydrogen and oxygen in the form of water are readily available in our atmosphere and are environmentally friendly. The study of the breakdown of water to produce hydrogen has been taking place since the 18th century, and electrocatalysis has maintained its consistency in the splitting of water till now. Electrochemical oxygen evolution reaction (OER) and hydrogen evolution reaction (HER) are the core technologies of water electrolysis for green hydrogen production. To address the challenges with water electrolysis, extensive research has been performed in the past decade, especially on the development of high‐performance and low‐cost electrocatalysts for OER and HER.^[^ ^3^ ^]^ Only in 2024, more than 13 000 papers have been published in this field. **Figure** **1**a shows the yearly published article number in the field of HER and OER.

### 1.1 Challenges for Water Electrolysis

HER is a process in which H^+^ ion is reduced at the cathode, and OER takes place at the anode, where oxidation of water takes place, as displayed in Figure 1b. Consequently, hydrogen and oxygen in the form of gas are liberated during the electrochemical water‐splitting. Despite significant progress over the past decades and the deployment of water electrolysis for hydrogen production in commercial applications, particularly alkaline water electrolysis, which has been a mature technology for nearly a century, water electrolysis still faces significant limitations due to high cost, poor activity and stability of the materials used at anode and cathode for OER and HER, respectively. The thermodynamic potential required for the initiation of electrochemical water‐splitting is 1.23 V with a Gibbs free energy of ΔG = 273 kJ mol^−1^. In practical operation, the required energy is greater than 1.23 V, and this extra potential is known by the name of overpotential. An electrocatalyst can be used to reduce this overpotential for the phenomenon to take place. It is reported that the best materials for HER are platinum (Pt), palladium (Pd), and ruthenium (Ru); and for OER are ruthenium oxide (RuO_2_) and iridium Oxide (IrO_2_). However, their high costs and scarcity restrict the widespread use of this clean technology. For instance, South Africa holds ≈80% of the world's Pt reserves; Ru and Ir are even rarer, mostly found in Russia and South Africa.^[^ ^4^ ^]^ To overcome this barrier, a lot of research has been conducted to develop Pt‐group metal‐free (PGM‐free) electrocatalysts, such as Ni, Co, etc.^[^ ^5^, ^6^ ^]^ Among the reported new electrocatalyst development strategies, bifunctional electrocatalysts, where the same catalyst material has the bifunctional activity to catalyze both the HER and OER, attract fast‐growing efforts. With a bifunctional electrocatalyst, material synthesis and electrode fabrication are simplified. Furthermore, a water electrolysis system with these single bifunctional electrocatalysts simplifies the setup of electrolyzers and decreases the overall cost as well.^[^ ^7^, ^8^ ^]^

During water‐splitting, slow kinetics of HER and OER pose significant challenges, limiting the overall efficiency of the process. Many catalysts, including even precious metals used in alkaline conditions, also lack sufficient activity to drive these reactions efficiently, resulting in lower hydrogen production. The sluggish kinetics of both reactions hamper the performance, necessitating more effective electrocatalysts to boost efficiency and scalability. Therefore, to improve water‐splitting performance, the development of advanced electrocatalysts is essential. The optimized heterointerfaces, involving complex engineering to facilitate better electron transfer, are thus required to enhance the reaction kinetics in this regard.^[^ ^9^ ^]^ Another key challenge is achieving the right balance of adsorption energies for reaction intermediates, which depends on precise control over the electronic structure of the catalyst.^[^ ^10^ ^]^ To address these challenges, various bifunctional electrocatalyst families, especially nanocomposites and heterojunctions showing synergistic effects with optimized heterointerfaces and balanced adsorption energies for reaction intermediates, have attracted extensive attention.

The rapidly growing interest in the bifunctional electrocatalyst has led to a large amount of work reported, and thus, many outstanding review papers have also been published to summarize this progress and provide an improved understanding of this field. Very recently, You et al. conducted a comprehensive review on bifunctional electrocatalysts for water electrolysis, including the physicochemical characterization techniques and application for both hybrid water splitting and overall water splitting. The review provided detailed discussions based on different metal elements, such as Ru, Ir, Fe, Co, Ni, and other mono metal or multi‐metal‐based catalysts.^[^ ^11^ ^]^ Like this work, Singh et al. specifically discussed the development of noble metal‐based, carbon‐encapsulated, and transition metal‐based bifunctional electrocatalysts.^[^ ^12^ ^]^ There are also quite a few other review papers focusing on a specific subject. Angaiah and co‐workers reviewed recent progress on noble metals and noble metals‐based hybrid materials as bifunctional electrocatalysts for overall water splitting.^[^ ^13^ ^]^ Tang et al. detailed the Ni‐based bifunctional catalysts for alkaline water electrolyzers.^[^ ^5^ ^]^ In particular, the differences in operating and testing conditions were outlined between laboratory and industrial systems, as well as the gaps in electrocatalytic equipment, evaluation methods, and preparation principles of electrodes. Based on these, the required academic efforts in advancing industrially relevant electrocatalysts were highlighted. The progress on other bifunctional electrocatalysts, such as those based on 2D metal organic frameworks (MOFs),^[^ ^14^ ^]^ MXene,^[^ ^15^ ^]^ Fe–N–C– and Co–N–C,^[^ ^16^ ^]^ and cobalt^[^ ^17^ ^]^ were all reviewed, as well as carbon fiber‐supported bifunctional electrocatalysts.^[^ ^18^ ^]^ Tang et al. also discussed the bifunctional electrocatalysts for the overall seawater splitting application.^[^ ^19^ ^]^

However, all these review papers discussed the bifunctional electrocatalysts mainly based on their element material. There is no work conducted to comprehensively review the influence of different families of bifunctional electrocatalysts and the synergistic effects between them, although this has been demonstrated, showing a key role for improving the catalytic performance. Therefore, in this work, we carry on a thorough review to highlight recent advances on different bifunctional electrocatalyst families with their structures, including MOFs, oxides, hydrides/hydroxides, carbon‐based, phosphides, and chalcogenides, and an assessment of their synergistic effect with nanocomposites and heterojunctions for both HER and OER. The requirements for designing a bifunctional electrocatalyst will be discussed. We will also conduct a systematic inter‐comparison of different material families, emphasizing their unique properties, performance metrics, and application potential, as well as providing valuable information on optimizing electrocatalyst design.

## 2 Mechanism and Affecting Factors for Water‐Splitting

### 2.1 Mechanisms for Water Electrolysis

Electrocatalytic water‐splitting, involving the breaking down of water molecules into hydrogen and oxygen, is a process that can be accomplished at room temperature. The procedure frequently uses electrocatalysts to enhance the rate of electrochemical reactions on the electrode surface to minimize energy consumption. The latest understanding of the mechanisms for HER and OER is discussed below at first to give a deeper insight into the electrocatalytic water‐splitting process.

#### 2.1.1 HER

**Figure 2:**
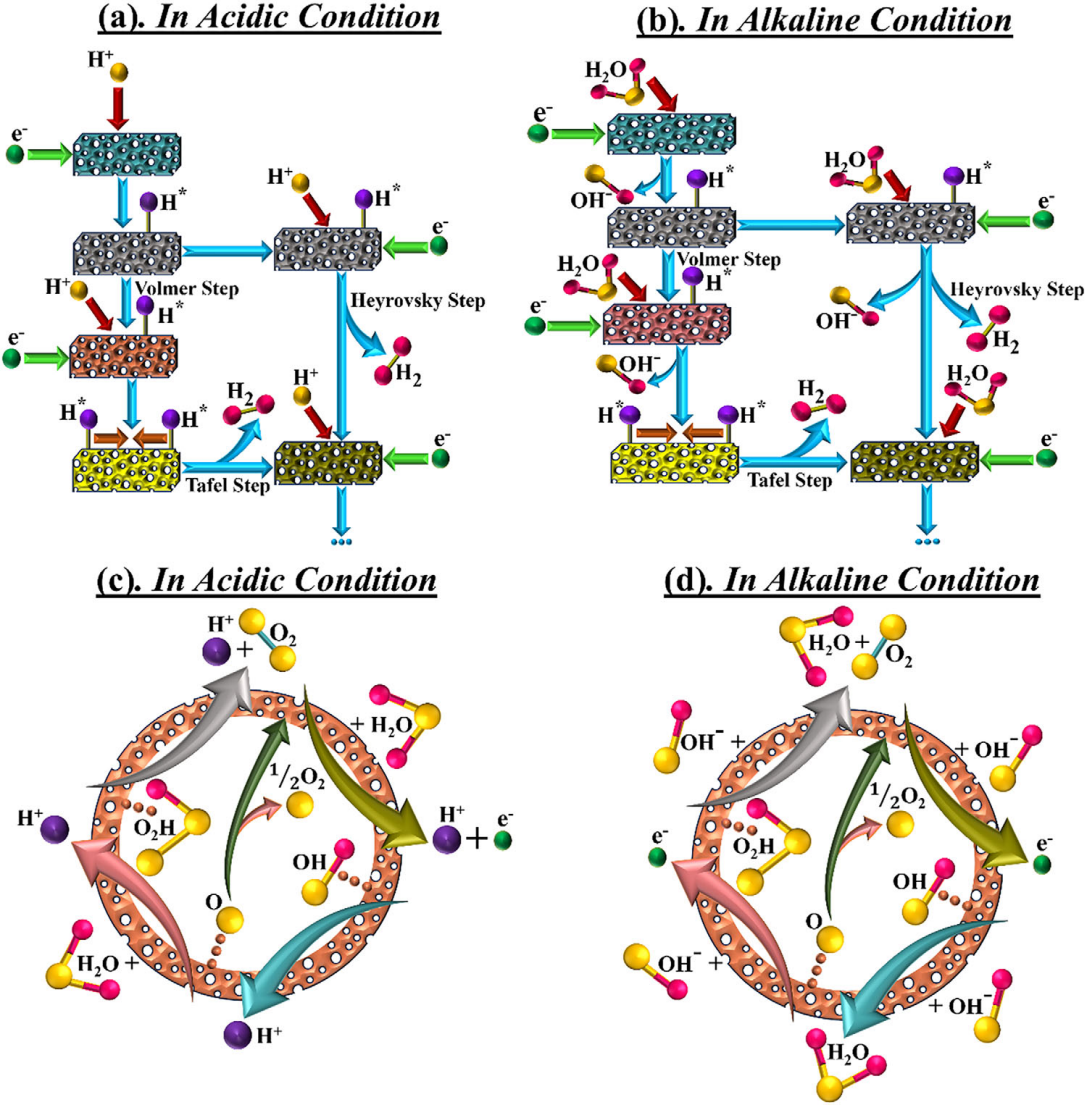
HER mechanism in a) acidic electrolyte and b) alkaline electrolyte, and OER mechanism in c) acidic electrolyte and d) alkaline electrolyte.

The typical HER process consists of two electron transfers. In case of an acidic electrolyte, protons (H^+^) are abundant. The reaction initiates with the adsorption of H^+^ from the electrolyte on the surface of the catalyst (H^*^), where it gains an electron, known as the Volmer reaction. The second step implies the desorption of hydrogen molecules (H_2_) from the surface of the cathode either through electrochemical means by adsorbing another H^+^ to form H─H bond (Heyrovsky reaction) or through a chemical route by interacting with another H^*^ (Tafel reaction). The whole mechanism is shown in **Figure** **2**a. The rate‐determining step (RDS) in acidic HER depends on the catalyst type: for noble metals like Pt, Tafel or Heyrovsky step often dominates, while for non‐noble catalysts, the Volmer step can be limiting due to weaker H^+^ adsorption.

For an alkaline electrolyte, the main distinction is the availability of protons in the Volmer step. Here, the adsorbed water molecules (H_2_O) initiate the reaction, followed by the H─O bond cleavage to form H^*^. Hydrogen is thus produced when H^*^ reacts with another H^*^ or adsorbed H_2_O. The mechanism is presented in Figure 2b. The Volmer step (dissociation of H_2_O) is widely regarded as RDS in alkaline HER, due to the high energy barrier associated with breaking the H─OH bond and additional competition from OH^−^ species for active sites. The HER efficacy in alkaline electrolyte is found to be roughly two or three times lower than that in the acidic electrolyte. The reason is water dissociation, the concentration of OH^−^ and H_2_O on active sites, and catalyst‐hydrogen bonding. There is a strong emphasis on the design of HER catalysts with superior capability to dissociate H_2_O while working in an alkaline condition.^[^ ^20^ ^]^

#### 2.1.2 OER

OER, being another half‐reaction of water‐splitting, generally demands a higher potential than HER, as it entails four‐electron transport, breaking of O─H bond, and formation of O═O. In acidic conditions, oxygen evolution initiates with the cleavage of H_2_O and subsequent production of H^+^. However, water hinders the formation of H^+^ as it is in equilibrium with H^+^ and OH^−^. The adsorption and desorption of various oxygen intermediates at the surface of the electrocatalyst, depending upon the bond strength between active sites and O‐ occurs, aided by electrolyte, leading to O_2_ evolution. The four‐electron transfer in an acidic electrolyte is shown in Figure 2c. In case of alkaline electrolyte, OER proceeds through the adsorbed hydroxyl ions (OH^*^) on active sites of the catalyst surface. The mechanism is evident in the formation of adsorbed O^*^ and OOH^*^ intermediates from oxidation of OH^*^ in Figure 2d, resulting in O_2_ evolution. A high concentration of OH^−^ facilitates the OER in alkaline conditions. Across both environments, the formation of OOH* intermediate is widely recognized as RDS. This step involves a high energy barrier due to the difficulty in forming and stabilizing the O─O bond on most catalyst surfaces. According to theoretical studies and DFT calculations, the energy required to convert O* to OOH* is generally the highest among all OER steps, particularly due to the scaling relationships between adsorbed intermediates that limit simultaneous optimization.^[^ ^20^, ^21^ ^]^

In contrast to acidic and alkaline electrolytes, neutral electrolytes need higher driving potentials to attain the same values of current density (**J**) during the water‐splitting reaction. They prevent corrosion problems, resulting in the construction of an environmentally friendly water‐splitting system. However, complicated mechanisms, large ohmic losses, and low ionic concentration limit their commercialization.^[^ ^9^ ^]^

### 2.2 Parameters Affecting Water Electrocatalysis

There are several parameters that affect the water electrolysis, including pH of electrolyte, overpotential, Tafel slope, turnover frequency, faradic efficiency, electrochemical active surface area (ECSA), and stability of electrocatalysts. These factors are elaborated upon below:

#### 2.2.1 pH of Electrolyte

Electrolytes allow the transfer of charge carriers for the completion of the reaction and thus play an important role in water‐splitting. When an electrolyte is brought in contact with an electrocatalyst, its charges redistribute to a dynamic equilibrium of electrochemical potential. The surface of the electrocatalyst will get a positive charge when pH of the electrolyte is lower than the point of zero‐charge, and vice versa. The electrolysis reaction is influenced differently by pH of electrolytes. The majority of OER electrocatalysts offer excellent activity in alkaline electrolytes, while HER catalysts have consistently shown exceptional behavior in acidic electrolytes. Because of their high conductivity, aqueous solutions of NaOH and KOH are mostly used as alkaline electrolytes, whereas aqueous H_2_SO_4_ is typically employed as an acidic electrolyte. Wang et al.^[^ ^22^ ^]^ found that HER activity of carbon nanotube‐supported diiron dithiolato is higher in alkaline solutions (pH 13) compared to neutral (pH 7) and acidic (pH 1) solutions. Such a difference in HER activity across pH levels is attributed to the distinct electrochemical processes of the diiron cluster in various aqueous media. In an investigation, Liu et al.^[^ ^23^ ^]^ reported the HER activity at Au (111) in acidic solutions with varying pH levels to understand the intrinsic kinetic parameters and their dependence on pH through experimental measurements and kinetic simulations. The key finding was that the intrinsic rate constant for HER increases with an increase in pH, which contrasts with the general trend where the HER current decreases as pH increases, emphasizing the pH effects on electrocatalytic reactions require careful consideration.

#### 2.2.2 Overpotential

Overpotential (η) is considered one of the most crucial parameters when assessing the catalytic performance for water electrolysis. It can be defined as the difference between the thermodynamic cell potential and the cell potential that would be experimentally required to overcome the intrinsic kinetic barrier of water‐splitting. Overpotential at a particular **J** can be estimated by using Linear Sweep Voltammetry (LSV) polarization analysis. The value of overpotential at specified **J**, i.e., 10 mA cm^−2^ is widely referred to as a benchmark to evaluate HER and OER performance. Overpotential is closely related to water electrolysis efficacy, since its smaller value corresponds to higher electrochemical energy conversion. Overpotential is an inherent characteristic of a catalyst; thus, an appropriate catalyst is the key requisite to attain a smaller overpotential. The development of catalysts like SnS_2_/g‐C_3_N_4_ in ref. [24] and N‐ZnO/ g‐C_3_N_4_ (CNNZO) in ref. [25] by I. Fareed et al. enhances charge transfer efficiency and optimizes the active sites for electrocatalytic reactions, reducing the overpotential values required to drive the electrolysis process at a benchmark **J** of 10 mA cm^−2^.

#### 2.2.3 Tafel Slope

Tafel slope is an important aspect to understand the catalytic activity toward electrochemical reactions and provides valuable insights into the reaction mechanism. Tafel slope is obtained by the linear regime of the plot between overpotential and logarithm of **J** following the Tafel equation, η  =  *a*  +  *b* *log* (*j*/*j_O_*), where **J** denotes the current density at particular η. The Tafel slope is calculated in units of mV dec^−1^, which informs us about the additional potential needed to raise the current by a significant amount. The smaller the Tafel slope, the larger the change in **J,** and thus, fast kinetics. An ideal catalyst exhibits a low value for the Tafel slope to achieve a high reaction rate.

#### 2.2.4 Turnover Frequency

To ascertain the reaction kinetics, the turnover frequency (TOF) is thought to be a crucial factor. It can be described as the actual measure of the intrinsic activity of a catalyst. It provides information about the number of molecules generated per unit time and active sites. Mathematically, TOF can be deduced using the relation *TOF*  =  j*A/n*F*m, where A, n, F, and m represent the area of the working electrode, number of electrons transferred, Faradic constant, and mass of active sites, respectively. A higher value of TOF corresponds to increased electrocatalytic activity. However, the difficulty in identifying the precise number of active sites involved in the catalytic reaction leads to an unintentional error in determining TOF.^[^ ^26^ ^]^

#### 2.2.5 Faradic Efficiency

Faradic efficiency (FE) is defined as the ratio of experimentally formed hydrogen or oxygen to the theoretical hydrogen or oxygen production value. The standard water–gas displacement procedure or gas chromatography are used to measure the actual H_2_ or O_2_ generation, while chronopotentiometry or chronoamperometry investigation is used to estimate theoretical H_2_ or O_2_ yield. When FE is ≈90%, a water‐splitting electrocatalyst is considered appropriate for practical usage. However, FE is limited due to the secondary reactions at the surface of the electrode.^[^ ^26^ ^]^

#### 2.2.6 Electrochemical Active Surface Area

Catalytic activity is generally not directly related to the mass of a catalyst due to its morphology variations and reactive sites. To assess the surface area and availability of active sites while determining HER and OER activity is of utmost importance in water electrolysis. The accessible area of the catalyst to the electrolyte at which the electrochemical reaction proceeds is of interest and called the electrochemical active surface area (ECSA). Electrodes having different geometries result in different current values and hence electrolytic efficacy. One of the most common methods for calculating ECSA is the capacitance‐based approach. In this traditional method, cyclic voltammetry (CV) is performed at varying scan rates in the non‐faradaic region, allowing the double‐layer capacitance of the material to be measured, which can then be used to calculate ECSA. An alternative approach involves using EIS to capture the capacitance in both the non‐faradaic region and during faradaic degradation processes, enabling a more accurate ECSA determination. Another estimation technique involves Faraday's law and the Randles–Sevcik equation. This method uses the redox peak current from a voltametric sweep, as charge is passed through the system, to calculate the ECSA, applicable in various pH ranges and environments.^[^ ^27^ ^]^

#### 2.2.7 Stability

**Figure 3:**
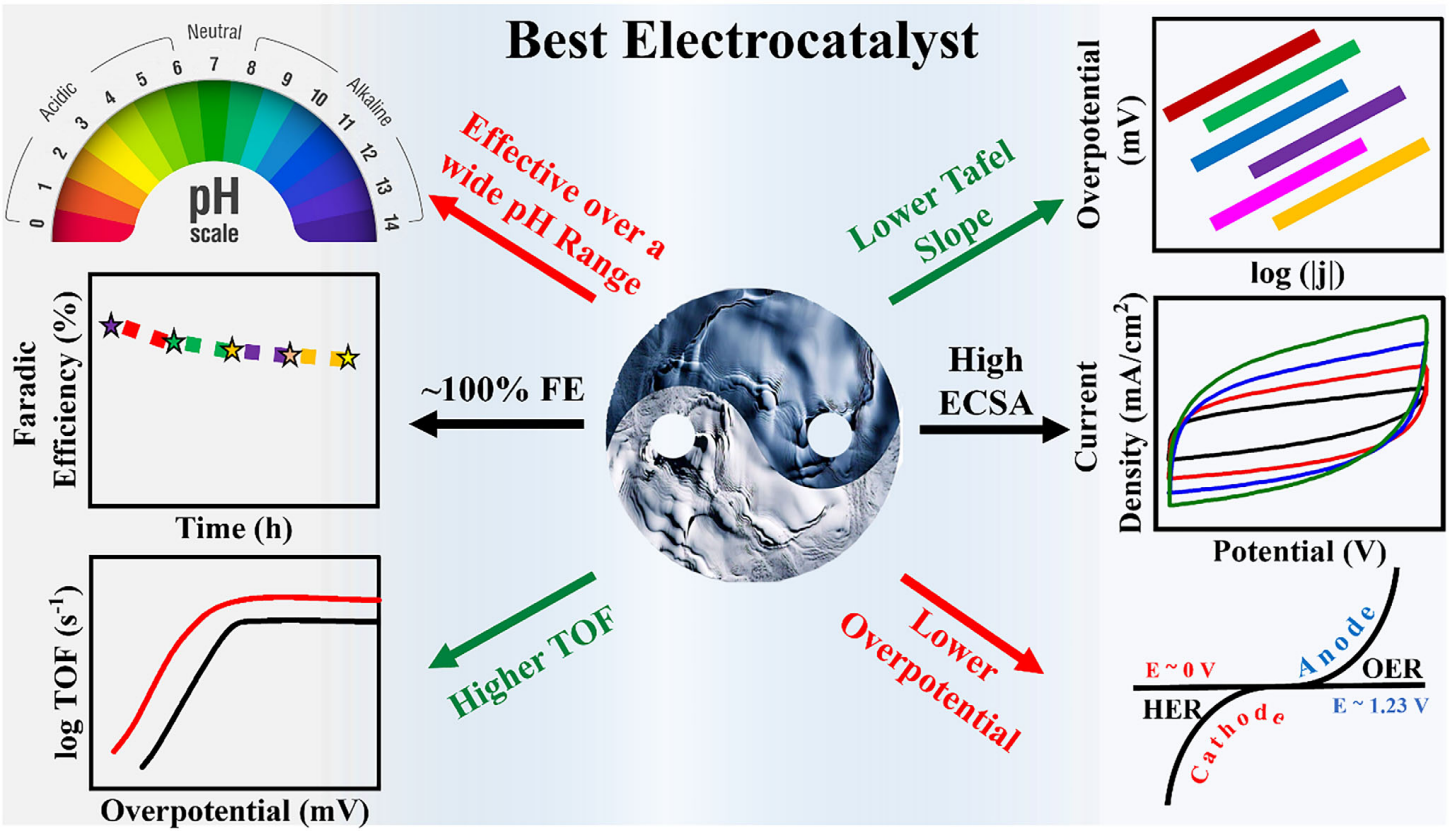
The key parameters of an ideal electrocatalyst. It should be active across all pH ranges, exhibit low overpotential and Tafel slope for high efficiency and fast reaction kinetics, achieve near‐100% Faradaic efficiency, and maintain long‐term stability for extended usability. Additionally, a large ECSA is essential for maximizing catalytic activity.

From the point of view of commercialization, long‐term stability of an electrocatalyst is regarded as a decisive factor. The catalyst is subjected to a chronopotentiometry trial (at an applied η) or a chronoamperometry examination (at a particular **J**) to record the variation in η and **J** values with time, respectively. The stability can also be determined by comparing the alterations in LSV polarization curves before and after multiple scans. A smaller change in activity is acceptable toward improved HER and OER; however, a sudden increase of η or decrease of **J** is ascribed to the loss of catalytic activity of an electrocatalyst. I. Fareed et al.^[^ ^28^ ^]^ developed a Ni‐doped Co(OH)_2_ catalyst (NCHNF) for improved OER, and its efficacy was evaluated over 1000 cycles. Minimal changes in catalytic activity were observed, underscoring the material's remarkable efficiency and stability in alkaline KOH solution for extended use. The key parameters for an ideal catalyst are schematically displayed in **Figure** **3**.

### 2.3 Inspiration for Design of Electrocatalyst

The rational design of electrocatalysts for water splitting necessitates a comprehensive evaluation that integrates both intrinsic catalytic activity and practical applicability. Optimal electrocatalysts are expected to exhibit low overpotentials, high current densities, and elevated TOF, thereby ensuring efficient HER and OER under operational conditions. However, catalytic activity alone is insufficient; Faradaic efficiency must also be maximized to ensure product conversion and suppress undesired side reactions. In parallel, compatibility with various industrial electrolyzer platforms governs material selection and electrode architecture. From a compositional standpoint, elemental abundance and environmental toxicity of active components are critical considerations, with preference toward earth‐abundant, nontoxic constituents. The scalable, cost‐effective, and environmentally benign fabrication pathway is more amenable to industrial integration. Furthermore, life‐cycle considerations such as recyclability, environmental impact, and disposal safety contribute to the overall sustainability profile of the electrocatalyst. Collectively, these factors define a multidimensional design strategy aimed at developing electrocatalysts that are not only catalytically efficient and stable but also scalable, sustainable, and industrially relevant.

## 3 Materials for Water Electrocatalysis

Based on the preceding discussion, the commercialization of electrolytic cells necessitates the exploration of economical and earth‐abundant electrode materials for efficient electrocatalysis and the production of hydrogen as a form of green energy from water sources. In pursuit of this research, numerous groups have contributed valuable insights to potentially replacing traditional electrode materials and improving cell performance. Xiong et al.^[^ ^29^ ^]^ presented a critical review on iron‐based catalysts supported by carbon materials, MOFs, and LDHs; the authors state that although they are economical, they have shown poor catalytic activity and stability. Mir et al.^[^ ^30^ ^]^ discussed Mo_2_C as a low‐cost electrocatalyst but mentioned issues such as carbon deposition and nickel dependency. Liu et al.^[^ ^31^ ^]^ reviewed seawater‐splitting but faced chlorine chemistry and impurity effects. Kulkarni et al.^[^ ^32^ ^]^ highlighted LDH‐carbon nanocomposites treated with plasma but raised concerns on scalability, high costs, and energy consumption. Unnikrishnan et al.^[^ ^33^ ^]^ mentioned carbon quantum dots with high conductivity but only in specific frameworks. Mohammed‐Ibrahim et al.^[^ ^34^ ^]^ provided an overview of earth‐abundant electrocatalysts with promising stability but lacking critical analysis. Aghamohammadi et al.^[^ ^35^ ^]^ described the insights into metal bromides but found incomplete studies requiring significant investment. Zubair et al.^[^ ^36^ ^]^ focused on MXene heterostructures but marked synthesis challenges. Chantenet et al.^[^ ^37^ ^]^ provided a comprehensive thermodynamic and computational analysis, while Quan et al.^[^ ^11^ ^]^ emphasized characterization techniques to assess the capability of the electrocatalysis process. Moreover, single‐atom bifunctional electrocatalysts have recently emerged as a promising frontier in electrocatalysis, offering unique advantages such as high atomic efficiency, adjustable coordination environments, and remarkable catalytic performance. Reviews by Zhang et al.,^[^ ^38^ ^]^ Li et al.,^[^ ^39^ ^]^ and Q. Zhang et al.^[^ ^40^ ^]^ have presented the growing interest in these materials, particularly for applications in zinc‐ion batteries and other redox‐driven systems. Despite their theoretical potential, experimental validation in comprehensive HER and OER electrocatalysis remains limited, indicating a need for further exploration to realize their full capabilities.

These works open gaps in material diversity, scalability, performance metrics, and insights that our review is answering through cost‐effective abundant materials, sustainability with an improved catalytic activity, and scalability for industrial applications. With innovation, the race to become a better material for electrocatalysis compels them to reach higher and higher.

Before delving into a comprehensive examination of diverse materials as potential electrocatalysts, it is essential to establish the common characteristics that define a good electrocatalyst. Fundamental characterization of an electrocatalyst involves studying the overpotential (mV) at a **J** of 10 mA cm^−2^ (**J**_10_) for OER and −10 mA cm^−2^ for HER, denoted as η. If a study examines overpotential at some different **J**, the value will be indicated as a subscript to η. Overpotential values are categorized as follows: 0 to 100 mV is considered ultralow, 100 to 200 mV is low, 200 to 300 mV is moderate, and values exceeding 300 mV are classified as high overpotential, for a better understanding of the reader. The materials are categorized into different subcategories and arranged in order from least effective to most effective for hydrogen production via electrocatalytic HER. The classification is defined as follows:

### 3.1 Metal–Organic Framework (MOF)

**Figure 4:**
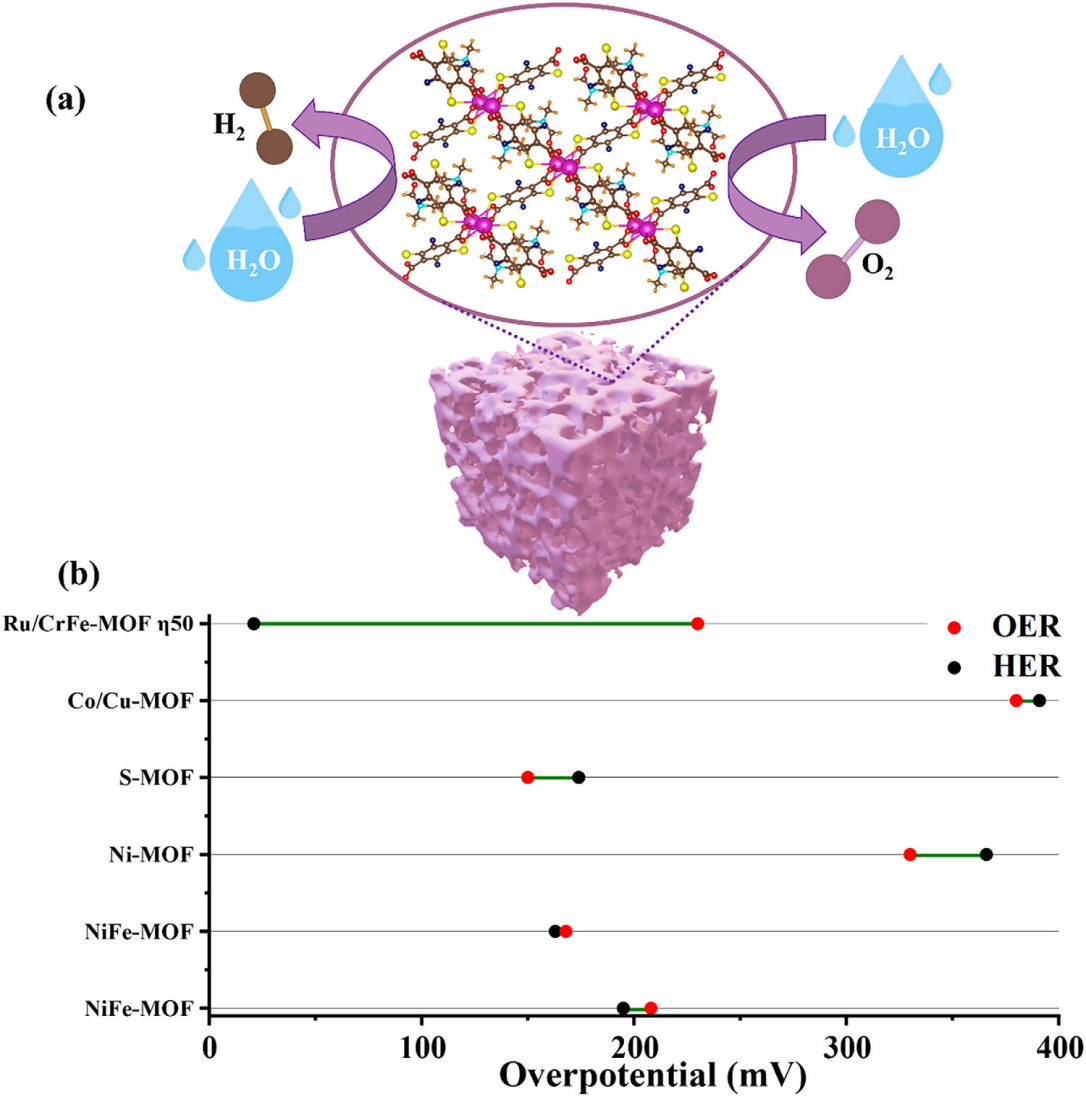
a) Porous structure of MOFs, zooming in on their crystal arrangement. The unique atomic configuration within the structure facilitates improved electrocatalytic performance and b) Intercomparison of HER and OER η for MOFs, based on **Table** **1**.

Metal Organic Frameworks (MOFs) provide an architecture decorated with suitable functional groups. These highly ordered carbon‐based materials possess high porosity, with nanopores below the range of 100 nm as schematically presented in **Figure** **4**a. The complex structures comprise of layers, clusters, chains, and coupled organic linkers, contributing to their performance in electrocatalysis.^[^ ^41^ ^]^

**Figure 5:**
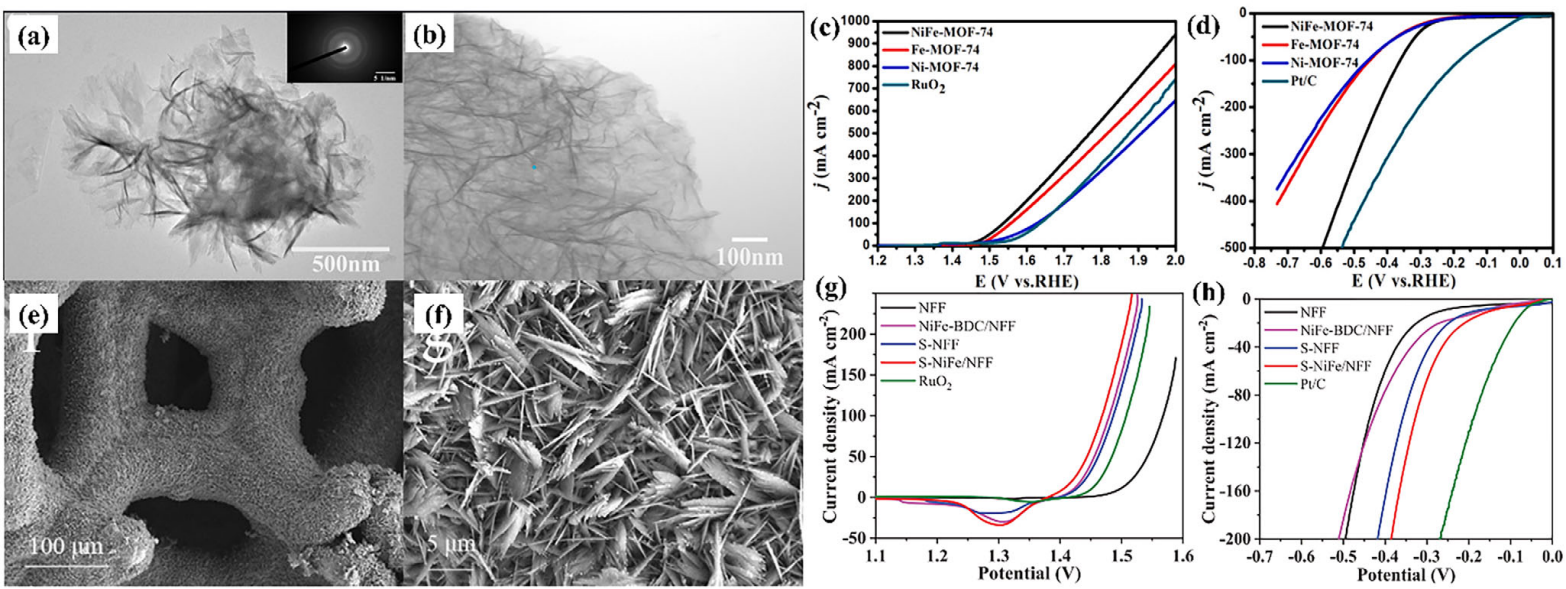
a,b) TEM images showing the morphology of NiFe‐MOF, c) its OER performance and d) HER activity. e,f) Comparative SEM images of NiFe‐MOF and S‐NiFe‐MOF/NFF, g) their OER activity, and h) HER activity, illustrating the enhanced catalytic performance of S‐NiFe‐MOF/NFF. Reproduced from ref. [42] and ref. [43] with permission of Elsevier.

In contrast to conventional 3D bulk MOFs, 2D MOFs are now considered better electrocatalysts due to several benefits, including the increased surface area with more accessible active metal sites and a nanometer thickness for effective mass transport. Chen et al.^[^ ^42^ ^]^ used a one‐step solvothermal (ST) technique to fabricate 2D NiFe‐MOF‐74 nanosheets via direct growth on Nickel Foam (NF), avoiding the need for an annealing procedure. Sheets of NiFe‐MOF‐74 combined to form an intricate network resembling a honeycomb structure; the images are shown in **Figure** **5**a,b. For OER, NiFe‐MOF‐74 achieved a moderate η: 208 mV, while a low η: 195 mV for HER, showing better activity than that of MOF‐74 and benchmark catalyst RuO_2_, summarized in Figure 5c,d. Similarly, NiFe‐based MOF supported on Ni foam was also studied by Mou et al.^[^ ^41^ ^]^ to achieve good OER and HER activities in alkaline solutions. The electrocatalyst presented low η: 168 mV and η: 163 mV for OER and HER, respectively. Furthermore, the catalyst only needed a cell voltage of 1.57 V for overall water‐splitting. In another research, using a straightforward ST technique, Lin Wu et al.^[^ ^43^ ^]^ created a self‐supported S‐NiFe‐MOF/NFF catalyst by sulfurating NiFe‐based MOF on NiFe foam, with the morphology being seen by SEM and TEM images shown in Figure 5e,f. The catalyst S‐NiFe‐MOF/NFF exhibited electrocatalytic activity for OER and HER with low η: of 174 mV and 150 mV in alkaline electrolyte, as shown in Figure 5g,h. S‐NiFe‐MOF/NFF cells merely require 1.50 V for overall water‐splitting.

MOFs consisting of arrays of metal complexes have emerged as a promising class of materials for electrocatalysis due to their high surface area, tunable porosity, and structural versatility. As summarized in Table 1 and Figure 4b, various MOF‐based catalysts have demonstrated potential for water electrolysis. However, most MOFs exhibit poor intrinsic electrical conductivity (≈10^−8^ S cm^−1^), which severely limits efficient electron transport to the catalytically active sites.^[^ ^44^ ^]^ In addition, many MOFs suffer from structural instability under harsh electrochemical conditions, such as acidic or alkaline aqueous media, where they tend to undergo hydrolysis or collapse. These limitations reduce their catalytic efficiency and long‐term durability.^[^ ^45^ ^]^ Furthermore, the microporous nature of many MOFs poses challenges for electrolyte infiltration and mass transport of reactants and products, hindering overall reaction kinetics.^[^ ^46^ ^]^ In situ studies have shown that MOFs, such as ZIF‐67 and NiCo‐MOF, often act as precatalysts, transforming into catalytically active metal hydroxides or oxides during operation.^[^ ^47^ ^]^ To overcome these issues, the development of 2D MOFs has gained attention, as they offer improved charge transport, higher exposure of active sites, and more accessible pathways for mass transfer, factors critical for enhancing electrocatalytic activity. Nevertheless, this subclass of materials requires further exploration to realize lower overpotential and favorable Tafel slope values. Metal oxides, by contrast, address some of these limitations by providing better chemical stability, enhanced conductivity, and a variety of active sites that improve catalytic performance, particularly in reactions like HER and OER.^[^ ^48^ ^]^

**Table 1:** MOF‐based catalysts for water electrolysis.

Electrocatalyst	Synthesis	Morphology	Electrolyte	Hydrogen evolution		Oxygen evolution		Refs.
				η [mV]	Tafel Slope [mV dec^−1^]	η [mV]	Tafel Slope [mV dec^−1^]	
NiFe‐MOF	ST	Nanosheet	KOH	195	136	208	54	[42]
NiFe‐MOF	ST	Nanosheets	KOH	163	139	168	42	[41]
Ni‐MOF	Pyrolysis	Nanospheres	H_2_SO_4_ (HER) KOH (OER)	366	93	330	54	[49]
S‐MOF	ST	Nanosheets	KOH	174	81	150	60	[43]
Co/Cu‐MOF	HT	Bulk Crystals	KOH	391	94	380	49/94	[50]
Ru/CrFe‐MOF	ST	Nanoclusters	KOH	21	25.91	230 η_50_	60.1	[51]

### 3.2 Metal Oxides

**Figure 6:**
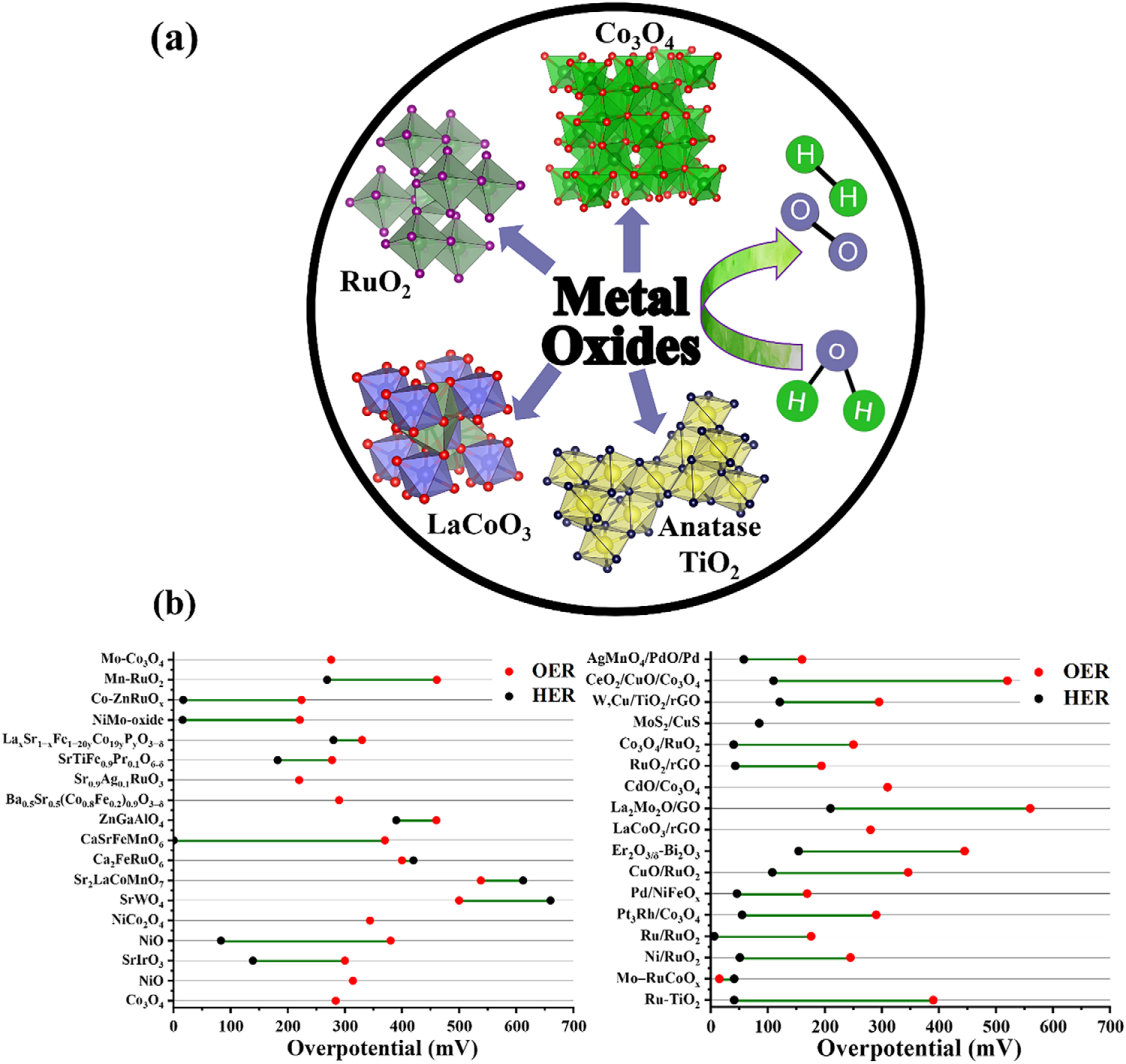
a) The schematic highlights different types of metal oxides, including transition metal oxides (TiO_2_, Co_3_O_4_), rare earth metal oxides (RuO_2_), and perovskites (LaCoO_3_), as promising materials for water electrolysis and b) Intercomparison of η for HER and OER of metal oxides, based on **Table** **2**.

Metals, particularly those in the transition metal series, are promising candidates for electrocatalysis due to their low cost and attributes comparable to noble metals. Although noble metals provide the best efficiency, their high cost and scarcity make them commercially unsuitable. Utilizing metal oxides, perovskites, and mixed metal oxides can yield attractive and favorable results, which can further be enhanced through doping, deposition, and heterojunction formation.^[^ ^52^, ^53^ ^]^ **Figure** **6**a provides a schematic representation of metal oxide‐based electrocatalysts for water‐splitting. The capability of some of the metal oxide catalysts is discussed below.

Elakkiya et al.^[^ ^54^ ^]^ introduced a versatile method to synthesize highly ordered nanomaterials of 2D metal oxide, specifically Co_3_O_4_ nanosheets. These nanosheets served as effective electrocatalysts for OER, demonstrating the η: 384.0 mV and Tafel slope of ≈52.0 mV dec^−1^ in 1 m KOH. Similarly, novel molybdenum‐doped ruthenium‐cobalt oxide (Mo‐RuCoOx) nanosheet array was developed by Zhang et al.^[^ ^55^ ^]^ as a high‐performance bifunctional electrocatalyst for OER and HER. The theoretical calculations and experimental results show that the electronic structure can be effectively tuned by incorporating Ru and Mo. Additionally, the kinetics for both HER and OER can be greatly accelerated by controllably coupling Mo dissolution with the generation of oxygen vacancies during surface reconstruction to optimize the adsorption energy of hydrogen/oxygen intermediates. Mo‐RuCoOx nanoarrays for HER and OER showed ultralow η: 41 and low η: 156 mV in 1 m KOH, respectively, with Tafel slopes of 24.3 and 69.1 mV dec^−1^. Furthermore, Mo‐RuCoOx catalyst demonstrated efficacy for water‐splitting, obtaining η: 1.585 V in neutral media, i.e., (1 m Phosphate Buffer Saline (PBS)). In an investigation, Li et al.^[^ ^56^ ^]^ offered the interconnected Ru‐cluster/carbon hybrid micro sheet (called Ru@V‐RuO_2_/C HMS) with a sub‐nanometer RuO_2_ skin that is rich in oxygen vacancies as a useful modification strategy, that can inherit HER activity of Ru and more importantly, activate the superior activity toward OER in both acidic and alkaline conditions. The final product retained the initial sheet‐like morphology, decorated with small nanostructures. Ru@V‐RuO_2_/C HMS illustrated an ultralow η: of 46/6 mV having Tafel slope of 55.1/45.1 mV dec^−1^ for HER and low and moderate η: 176/201 mV having Tafel slope of 104.9/114.3 mV dec^−1^ for OER, respectively, in acidic/alkaline solution. Inspiringly, the cell voltage of 1.467/1.437 V at J10 in 0.5 m H_2_SO_4_/1 m KOH drove the overall water‐splitting.

**Figure 7:**
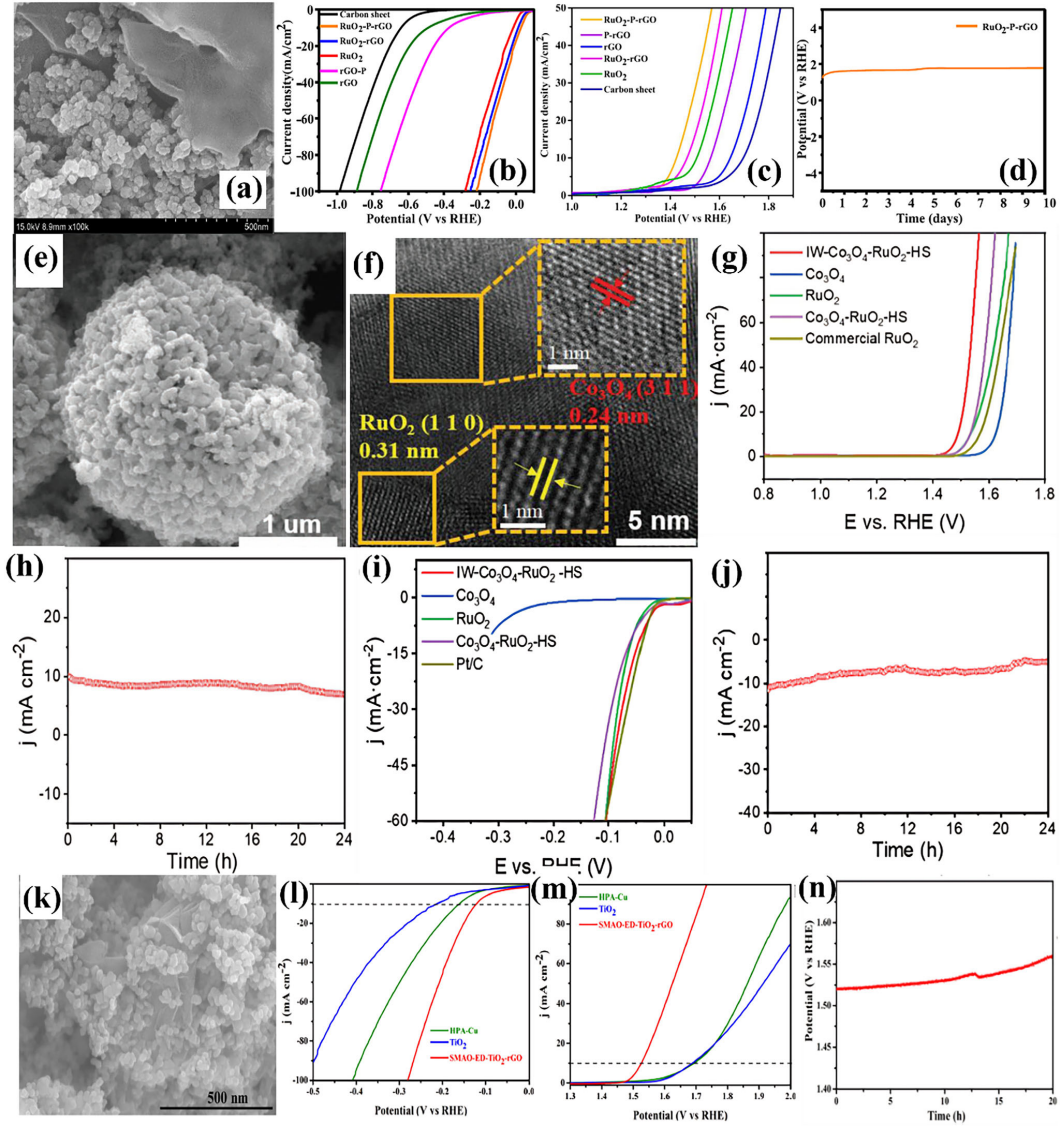
a) TEM image of RuO_2_‐P‐rGO, b,c) its HER and OER polarization curves, d) Chronopotentiometry curve (for 10 days) of RuO_2_‐P‐rGO, e,f) SEM and HRTEM images of IW‐Co_3_O_4_‐RuO_2_‐HS, g,h) OER curve and stability test for 24h, i,j) HER and stability test of IW‐Co_3_O_4_‐RuO_2_‐HS for HER, k) SEM image of SMAO‐ED‐TiO_2_‐rGO, with l,m) Polarization curve for HER and OER and n) Stability for 20h. Reproduced from,^[^ ^52^, ^57^, ^58^ ^]^ with permission of Wiley and Elsevier.

For improved electrocatalytic HER and OER activity, Raja et al.^[^ ^57^ ^]^ synthesized RuO_2_ particles decorated on a phosphate‐doped reduced graphene oxide (rGO) layer. A few‐layer 2D plane with a transparent sheet‐like structure is shown in **Figure** **7**a. RuO_2_‐P‐rGO showed an ultralow η: 43 mV for HER in 0.5 m H_2_SO_4_ with Tafel slope of 28 mV dec^−1^, as exhibited in Figure 7b. While the electrocatalyst had a low Tafel slope of 94 mV dec^−1^ and a low η: 194 mV for OER with excellent stability and minimal change in potential with respect to time, as displayed in Figure 7c,d. For the same purpose, a simple and environmentally friendly ST low‐temperature oxidation process, followed by ice water treatment, was used by Gao et al.^[^ ^52^ ^]^ to produce Co_3_O_4_‐RuO_2_ hollow spheres (IW‐Co_3_O_4_‐RuO_2_‐HS). The hollow nanostructure can be visualized from Figure 7e,f, which may be able to supply enough channels and active sites for electrocatalytic processes. Toward HER, ultralow η:40 mV and for OER, low η:250 mV was presented by IW Co_3_O_4_‐RuO_2_‐HS, with a Tafel slope of 42.6 and 55.4 mV dec^−1^, respectively. The corresponding LSV polarization curves for OER and HER are shown in Figure 7g,i. The catalyst further illustrated excellent stability for practical usage, as shown in Figure 7h,j. Similarly, single metal–atom oxide (metal: W/Cu) attached on TiO_2_‐rGO nanomaterials (SMAO‐ED‐TiO_2_‐rGO) was reported by K. Selvakumar et al.^[^ ^58^ ^]^ using a straightforward sonication technique. High‐angle annular dark‐field scanning transmission electron microscopy (HAADF‐STEM) image in Figure 7k identified the uniform dispersion of W/Cu metal atom oxide over TiO_2_‐rGO material. With a η:121 (low)/295 (moderate) mV and low Tafel slope of 96/60.3 mV dec^−1^ in 1 m KOH solution, SMAO‐ED‐TiO_2_‐rGO nano‐catalyst has demonstrated remarkable HER/OER activity, as provided in Figure 7l,m. During 20 h of operation, SMAO‐ED‐TiO_2_‐rGO exhibited 1.56 V, a progressive increase from 1.52 V as displayed in Figure 7n, ensuring stable performance for practical usage.

Karmakar et al.^[^ ^59^ ^]^ presented a rapid microwave‐assisted synthesis approach to fabricate NiMoO_4_ nanorods within a mere 30‐min reaction time. These nanorods, initially grown on NF, underwent treatment with NaBH_4_ to induce internal oxygen vacancies [NiMoO_4_(Vo)]. The introduction of oxygen vacancies into the lattice structure modulated the electronic configuration, promoting sustainable OER activity. Under alkaline conditions, vacancy‐enriched NiMoO_4_ (Vo) nanorods exhibited moderate η: 220 mV for OER with a Tafel slope of 104 mV dec^−1^ and moderate η: 255 mV for HER with a Tafel slope of 30 mV dec^−1^ at a **J** of 50 mA cm^−2^, supported by EIS investigations. The stability of OER and HER further ensured long‐term usage. In a practical two‐electrode system (with NiMoO_4_(Vo) nanorods serving as both anode and cathode), an η_50_: 490 mV was observed. Molecular orbital and band structure theories indicated that the formation of band gap states (BGS) generates antibonding states with low electron population, thereby facilitating efficient OER and HER kinetics.

Zhou et al.^[^ ^60^ ^]^ developed a Ni–Mo‐based metal/oxide heterostructure catalyst (NiMo M/O) by integrating polyoxometalates (POM) clusters onto Ni hydroxide precursor. This precursor featured interfaces created through the attachment of Ni_4_Mo nanoparticles onto Ni‐doped MoO_2_ nanosheets (Ni‐MoO_2_), verified via HRTEM analysis. Leveraging the etching influence of POM, the resulting catalyst displayed numerous mesopores on nanosheets, leading to a significant exposure of interfacial atoms within the catalyst. The unique Ni–Mo‐based metal/oxide heterostructure with abundant mesopores enables the interfacial Ni atoms to be exposed extensively. This catalyst recorded an ultra‐low η: 16 mV and Tafel slope of 31.9 mV dec^−1^ for alkaline HER. The results were supported by chronopotentiometry. As synthesized heterostructure exhibited remarkable OER performance as well, having a moderate η: 221 and Tafel slope of 82.9 mV dec^−1^. The catalyst also displayed stability for 40 000 sec. Similarly, Mondal et al.^[^ ^61^ ^]^ synthesized AgMnO_4_, palladium nanoparticles, and silver permanganate nanocomposite (AMO/PdO_x_/Pd), verified via HRTEM. By annealing the AMO/Pd nanocomposite, PdO_x_ (x = 1, 1.5, 2) was further modified. Mixed‐valence Pd (0, 2^+^, 3^+^) was formed at 260 °C, which provided considerable OER/HER activities. AMO/PdO_x_/Pd nanocomposite showed Tafel slopes of 64.9 mV dec^−1^ for OER, 37.8 mV dec^−1^ for HER, and low η: 160 mV for OER and ultra‐low η: 58 mV for HER. AMO/PdO_x_/Pd nanocomposite also provided exceptional long‐term stability and required just 1.50 V to split water molecules.

**Figure 8:**
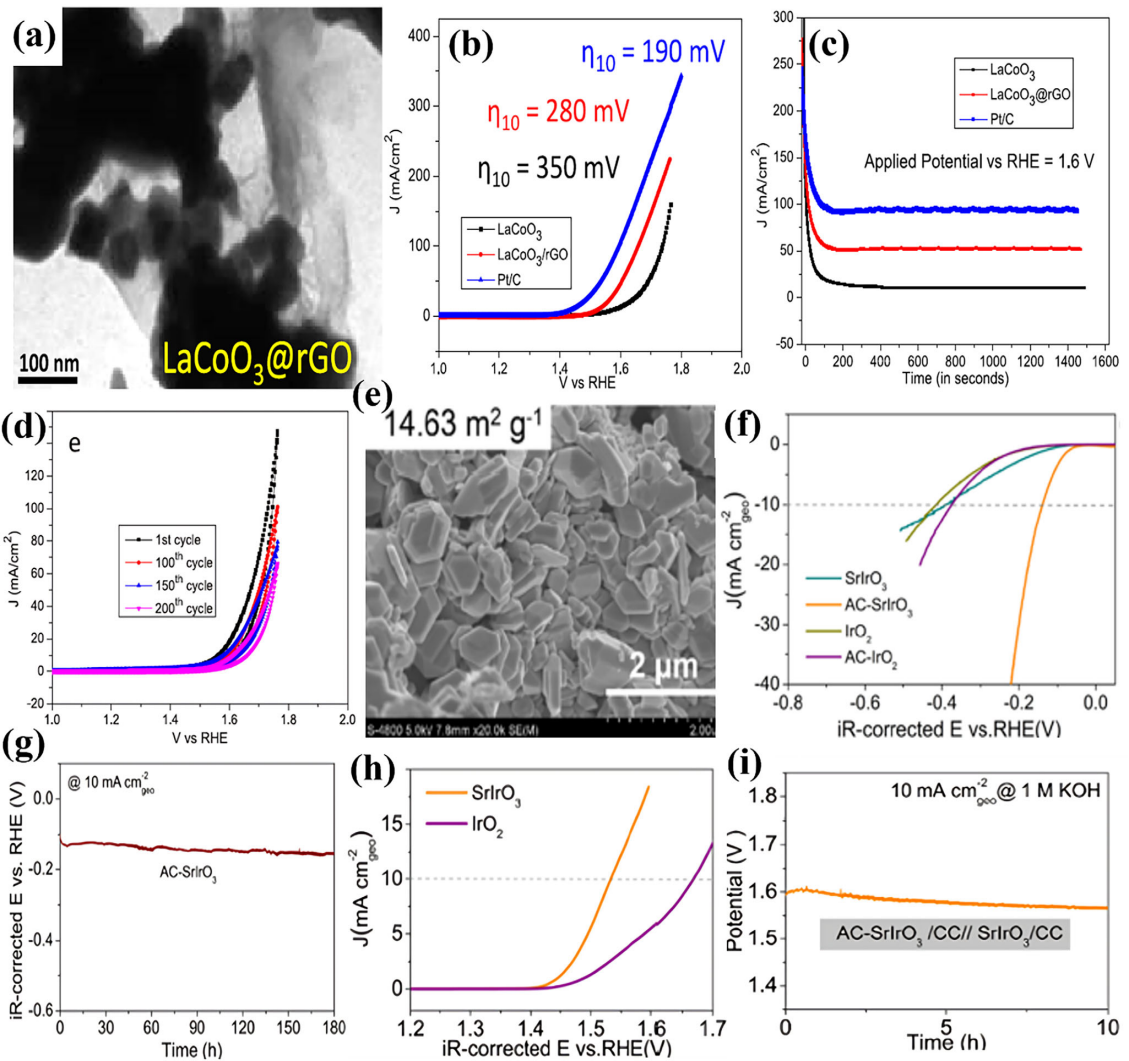
a) TEM image of LaCoO_3_@rGO, b) LSV curve and c,d) Cyclic stability curves of LaCoO_3_@rGO for OER, e) SEM image of AC‐SrIrO_3_, f,g) LSV curve and stability curve of AC‐SrIrO_3_ for HER, h,i) Polarization and chronoamperometric curves of AC‐SrIrO_3_ for OER. Reproduced from ref. [62] and ref. [63] with permission of Elsevier and American Chemical Society.

LaCoO_3_ for oxygen reactions (ORR and OER) may be modified for high surface area, strong electrical conductivity, and good chemical stability with rGO sheets. Electron transport during electro‐chemical oxygen reactions is effectively increased by rGO addition. rGO encapsulated perovskite‐type lanthanum cobalt oxide nanoparticles (LaCoO_3_@rGO) were synthesized by Ahmed et al.^[^ ^62^ ^]^ via an ultra‐sonication procedure for electrochemical OER in an O_2_‐saturated alkaline medium. LaCoO_3_‐encapsulated rGO nanosheets, shown in **Figure** **8**a, exhibit better electro‐catalytic performance toward OER, with moderate η: 280 mV and Tafel slope of 104 mV dec^−1^ when compared to the pure LaCoO_3_ electro‐catalyst, along with strong stability, evidenced in Figure 8b–d. In another research, Jie Yu et al.^[^ ^63^ ^]^ developed bulk‐phase metallic SrIrO_3_ perovskite with monoclinic structure using a solid‐state route under mild synthesis conditions, the morphology is displayed in Figure 8e. During the hydrogen‐evolution activation process in alkaline solution, monoclinic SrIrO_3_ exhibited enhanced activity through continuous reconstruction, facilitated by the leaching of lattice Sr^2+^. This led to the development of surface reconstruction‐derived catalyst AC‐SrIrO_3_, which demonstrated low η: 139 mV, Tafel slope of 49 mV dec^−1^, and stable operation for 180 h, presented in Figure 8f,g. The researchers identified the precise structure of reconstruction‐derived species and highlighted that improved electrical conductivity and increased active surface areas were pivotal for achieving outstanding performance for HER. Furthermore, SrIrO_3_ displayed remarkable catalytic behavior for OER in 0.1 m KOH, requiring moderate η: 300 mV with a Tafel slope of 42 mV dec^−1^, recorded in Figure 8h. The overall water‐splitting performance is also evaluated and provided in Figure 8i, which significantly surpassed most of the perovskite‐based oxides.

The performance of metal‐based electrocatalysts for water‐splitting is presented in Table 2 and Figure 6b. Among these, transition metal oxides, perovskites, and mixed metal oxides have gained considerable attention due to their low cost, abundant availability, and flexible synthesis into various dimensionalities (0D to 3D) and morphologies such as rod‐like, flower‐like, and polygonal structures. These materials have shown reasonably low overpotentials and favorable Tafel slopes for both HER and OER, establishing them as promising candidates for practical electrocatalytic applications. Catalytic performance can be further enhanced through nano‐structuring, defect/vacancy engineering, heteroatom doping, and substrate optimization. However, several intrinsic limitations hinder their long‐term applicability. Metal oxides, especially 3d transition‐metal oxides, typically exhibit poor electrical conductivity, which limits electron transport and reduces the number of catalytically active sites participating in the reaction. This results in higher resistance and lower mass activity.^[^ ^64^ ^]^ Additionally, during OER, substantial overpotentials beyond the thermodynamic 1.23 V versus RHE are required, making the process energetically inefficient. For HER, unfavorable adsorption energies of intermediates and sluggish kinetics contribute to higher Tafel slopes.^[^ ^65^ ^]^ Moreover, metal oxides are prone to surface reconstruction and degradation during electrocatalysis, including dissolution or enrichment/depletion of surface elements, which compromises their structural integrity and stability over time.^[^ ^64^ ^]^ These issues are particularly severe under acidic or industrial‐scale high‐current conditions, where even noble oxides such as RuO_2_ may undergo dissolution, and earth‐abundant oxides often fail to survive, rendering them incompatible with proton‐exchange membrane (PEM) electrolyzers.^[^ ^66^ ^]^ To overcome these challenges, shifting toward metal hydrides and hydroxides has proven advantageous, as these materials offer better electrical conductivity, more accessible active sites, and improved catalytic kinetics, particularly under alkaline conditions, resulting in enhanced electrocatalytic performance and long‐term operational stability.^[^ ^67^ ^]^

**Table 2:** Metal oxide‐based catalysts for water electrolysis.

Electrocatalyst	Synthesis	Morphology	Electrolyte	Hydrogen evolution		Oxygen evolution		Refs.
				η [mV]	Tafel slope [mV dec^−1^]	η [mV]	Tafel slope [mV dec^−1^]	
Co_3_O_4_	CR	Nanosheet	KOH	–	–	384	52	[54]
NiO	Electrochemistry	Micro flowers	KOH	–	–	314	40	[68]
SrIrO_3_	Solid‐State Reaction	Irregular particles	KOH	139	49	300	42	[63]
NiO	HT	Nanosheets	KOH	83	209	380	299	[69]
NiCo_2_O_4_	HT	3D Unchin‐like	KOH	–	–	344	71.99	[70]
SrWO_4_	HT, Coprecipitation, Ultra Sonification	Proximal phalanges, dumbbell, bar‐like	KOH	660	138	500	218	[71]
Sr_2_LaCoMnO_7_	SST^a)^	Microstructures	KOH	612	141	538	123	[72]
NiMoO_4_	Microwave Heating	Nanorods	KOH	255	104	220 η_50_	30	[59]
SnFeS_X_O_Y_	ST Method	Spherical nanoparticles	KOH	85 167 η_100_ 249 η_500_ 324 η_1000_	90	281 η_100_	60	[73]
Ca_2_FeRuO_6_	Solid‐state reaction	Microstructural	KOH	420	–	400	102	[74]
CaSrFeMnO_6_	SST Method	Irregular structure	KOH H_2_SO_4_	0.31	157 in acidic and 163 in basic	370	75	[75]
ZnGaAlO_4_	SST reaction route	–	KOH	390	–	460	–	[76]
Ba_0.5_Sr_0.5_(Co_0.8_Fe_0.2_)_0.9_O_3–δ_	Solgel and single‐site doping	Nanosheets	KOH	–	–	290	53	[77]
Sr_0.9_Ag_0.1_RuO_3_	Solgel	Nanoparticles	KOH	–	–	220	156	[78]
SrTiFe_0.9_Pr_0.1_O_6‐δ_	Electrochemical	Sponge like	KOH	182.4	77	277.6	73	[79]
La_x_Sr_1−x_Fe_1−20y_Co_19y_P_y_O_3−δ_	Solid‐state phase reaction	Cubic structure	KOH	280	119.2	330	53.2	[80]
NiMo‐oxide	Solution process	Nanosheets	KOH	16	31.9	221	82.9	[81]
Co‐ZnRuO_x_	Ion‐exchange	Nanocages	KOH	17	21.61	224	67.55	[82]
Mn‐RuO_2_	Electrospinning	Nanofibers	KOH	269	39.2	461	56.4	[83]
Mo‐Co_3_O_4_	HT Method	Nanoneedle	KOH	–	–	276	53.9	[84]
Ru‐TiO_2_	Electrolysis	Nanotubes	KOH	41	317	390	94	[85]
Mo–RuCoO_x_	MOF derived	Nanoarrays	KOH	41	24.3	15	69.1	[55]
Mn‐CoO	HT	Nanospheres	KOH	25.6	72.6	301 η_50_	96.7	[86]
Ni/RuO_2_	Glucose‐blowing approach	Nanocrystals	KOH	51	34.4	245	66	[87]
Ru/RuO_2_	Carbothermal	Nanoclusters	H_2_SO_4_ KOH	46 (acidic) 6 (alkaline)	55.1 (acidic) 45.1 (alkaline)	176 (acidic) 201 (alkaline)	104.9 (acidic)114.3 (alkaline)	[56]
Pt_3_Rh/Co_3_O_4_	CR^b)^	Spherical nanoparticles	N_2_ saturated HClO_4_	55	47	290	102	[88]
Pd/NiFeO_x_	HT	Nanosheets	H_2_SO_4_	46	38.56	169	76.56	[89]
CuO/RuO_2_	Pulsed laser ablation	Nanopores	KOH	108	98.4	346	85.7	[90]
Er_2_O_3/δ_‐Bi_2_O_3_	Biomimetic route	Nanoplates	KOH	154	–	445	–	[91]
LaCoO_3_/rGO	Sol‐Gel	Nanosheet	KOH	–	–	280	104	[62]
La_2_Mo_2_O/GO	Precipitation	Nanosheets	KOH	210	81	560	186	[92]
CdO/Co_3_O_4_	Co‐Precipitation	Nanowires	KOH	–	–	310	62	[93]
NiO/RuO_2_	HT	Nanosheets	KOH	20	42	250 η_50_	68.7	[94]
RuO_2_/rGO	HT	Nanosheets	H_2_SO_4_	43	92	194	231	[95]
Co_3_O_4_/RuO_2_	ST	Hollow spheres	KOH	40	42.6	250	55.4	[96]
MoS_2_/CuS	HT	Nanoflakes	KOH	85	80	–	–	[97]
W,Cu/TiO_2_/rGO	Sonication	Nano‐catalyst	KOH	121	96	295	60.3	[98]
Ru‐NiO/Co_3_O_4_	HT	Needle‐like array	KOH	138 η_100_	58	269 η_100_	59	[99]
CeO_2_/CuO/Co_3_O_4_	Coprecipitation	Nanorods	KOH	110	88	520	87	[100]
N‐C/NiFe_2_O_4_	HT	Coreshell	KOH	200 η_100_	59.6	230 η_100_	42	[101]
AgMnO_4_/PdO/Pd	Annealing	Nanorods	KOH	58	37.8	160	64.9	[61]

^a)^ Solid State Thermal Reaction;

^b)^ Chemical Reduction.

### 3.3 Hydrides/Hydroxides

**Figure 9:**
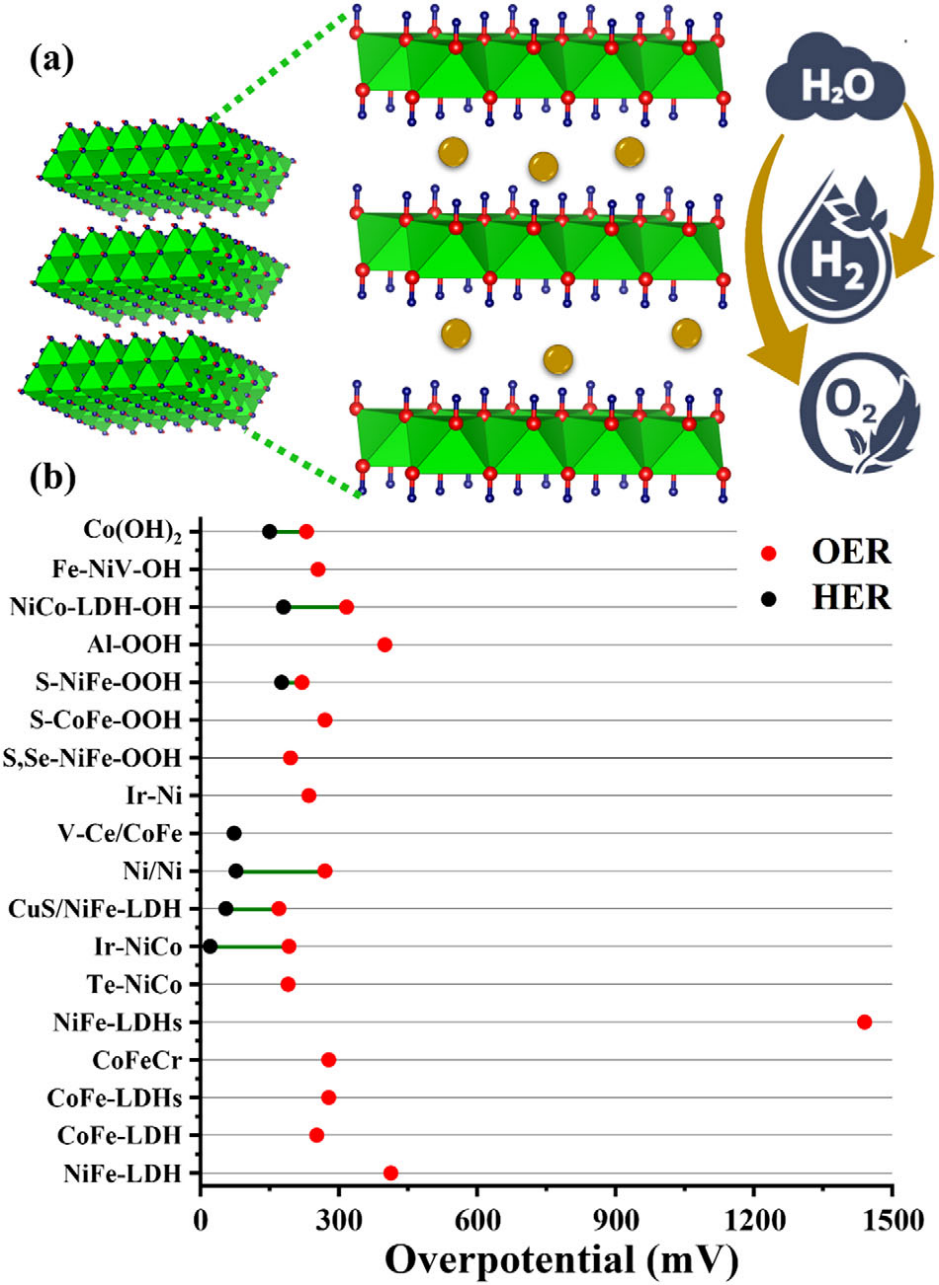
a) Schematic represents the structure of hydrides and LDH performing water electrocatalysis and b) Intercomparison of η for HER and OER of hydrides/hydroxides based on **Table** **3**, showcasing their efficient oxygen production.

A new class of materials known as hydrides, characterized by a high level of homogeneity, has the capability to promote efficient HER and OER. Hydrides utilize intermediates to facilitate hydrogen (H_2_) evolution, presenting an innovative approach in the field of electrocatalysis.^[^ ^102^ ^]^ The versatile structure of hydrides is given in **Figure** **9**a, and the efficacy toward water‐splitting is discussed below.

**Figure 10:**
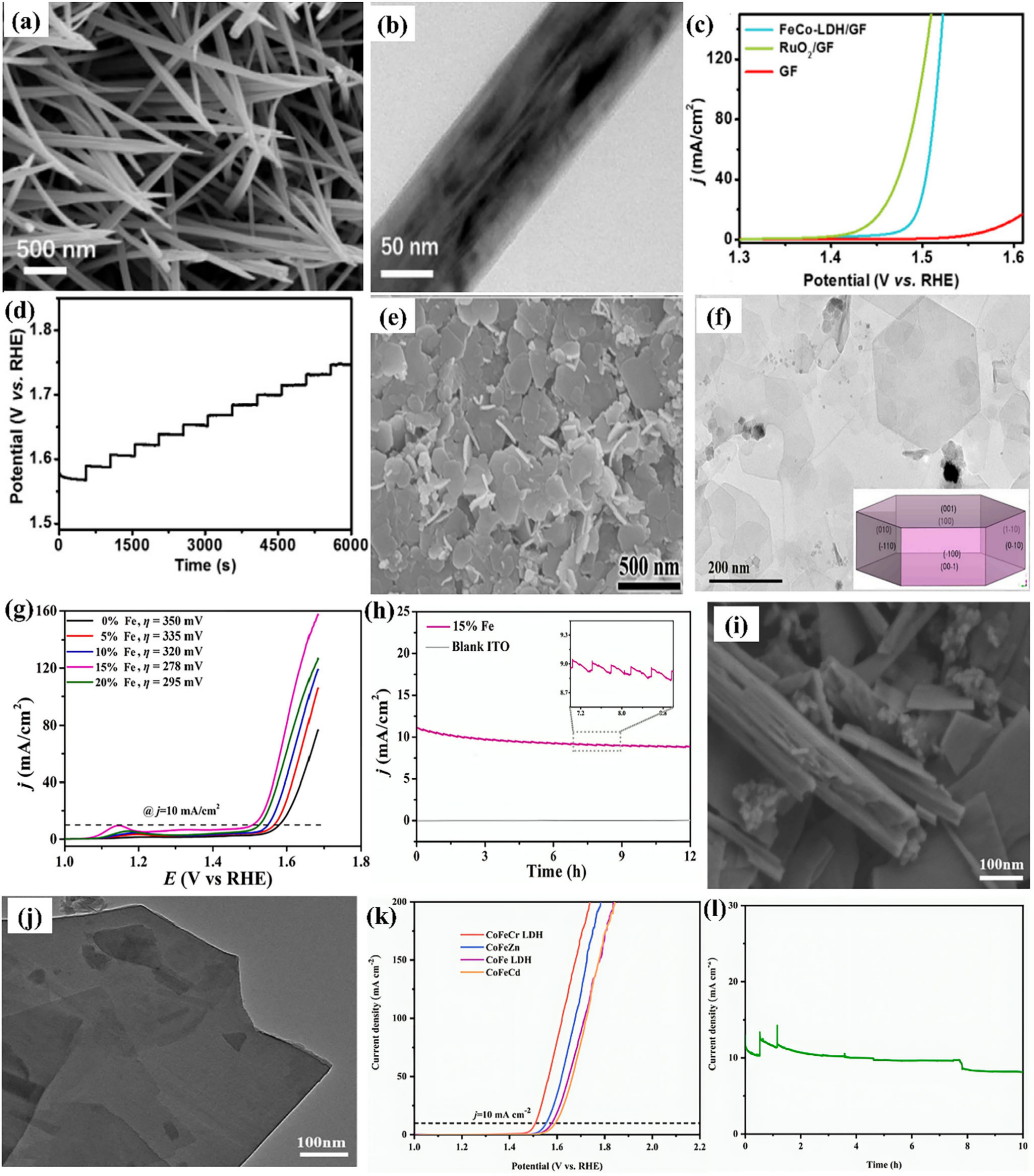
a,b) SEM and TEM images of CoFe‐LDH/GF, c) OER activity of CoFe‐LDH/GF, d) Multi‐current process of CoFe‐LDH/GF, starting at 40 mA cm^−2^ and increasing by 20 mA cm^−2^ every 500 s, up to 260 mA cm^−2^, e,f) SEM and TEM images of Co_0.85_Fe_0.15_‐LDHs, g) OER performance and h) stability test of Co_0.85_Fe_0.15_‐LDHs, i,j) SEM and TEM images of CoFeCr‐LD, k) OER performance and l) Stability analysis of CoFeCr‐LDH. Reproduced from,^[^ ^102^, ^103^, ^104^ ^]^ with permission of Elsevier.

Deng et al.^[^ ^103^ ^]^ created CoFe‐Layored Double Hydroxide (LDH) on graphite felt (CoFe‐LDH/GF) having a nanowire structure, evident from **Figure** **10**a,b. As a strong integrated 3D OER anode, CoFe‐LDH/GF showed good catalytic activity and required moderate η: 252 and η_100_: 285 mV in 1 m KOH; the activity is also displayed in Figure 10c. The electrochemical stability test via multi‐step chronopotentiometry in Figure 10d displayed that the material is stabilized at 1.57 V and remains unchanged for 500 s. In a similar way, a series of Co_x_Fe_y_‐LDHs nanosheets with varying Fe doping from 5% to 20% (molar ratio) were described by Wang et al.,^[^ ^102^ ^]^ surface visualization at the nanometer scale is provided in Figure 10e,f. Through electrochemical testing, CoFe_‐_LDH with 15% Fe doping executed moderate η_:_ 278 mV in terms of OER performance (Figure 10g). After 12 h of operation at 1.6 V (versus RHE), its electrolytic **J** dropped somewhat and stayed at ≈90% of the initial **J**, showing good stability, result is attached in Figure 10h. Furthermore, a simple one‐step hydrothermal (HT) approach was used by Lingxia Qiao et al.^[^ ^104^ ^]^ to introduce nonprecious chromium dopant into CoFe‐LDH. The morphology of CoFe‐LDH was akin to a nanorod‐like arrangement, as presented in Figure 10i,j. In contrast to CoFeZn, CoFe‐LDH, and CoFeCd, CoFeCr‐LDH displayed superior electrocatalytic oxygen evolution with moderate η: 278 mV, as well as high stability following 3000 cycles, attached in Figure 10k,l.

Fan et al.^[^ ^105^ ^]^ developed Ir‐doped NiCo‐LDH via ST method, followed by spontaneous galvanic displacement as a cost‐effective bifunctional electrocatalyst. Ni centers have a great affinity for water molecules and OH‐ intermediates, however, Co centers possess poor binding strength to hydrogen, resulting in a large kinetic energy barrier for the Volmer step and thus slowing down the HER. Ir doping improves the adsorption and binding strength and thus the kinetics for water‐splitting. Atomic Ir‐doped NiCo‐LDH presented vertically aligned interconnected nanosheet‐like morphology. As‐synthesized catalyst achieved exceptional overall water‐splitting performance, requiring a voltage of 1.45 V in 1.0 M KOH electrolyte, with superior durability over 200 h compared to NiCo‐LDH. Similarly, Sarfraz et al.^[^ ^106^ ^]^ deposited CuS on NiFe‐LDH to prepare an electrocatalyst for overall water‐splitting. CuS nanoparticles encapsulated NiFe‐LDH with spherical structure like morphology presented an ultra‐low η: 55 mV for HER and low η_30_: 170 mV for OER. The unique electronic configuration of Ni and Fe and their lamellar structure promoted electrical conductivity, while CuS provided additional reactive sites for electrochemical reaction. The synergistic effects of CuS and NiFe‐LDH are responsible for boosted electrocatalytic performance. With a cell voltage of 1.51 V, the composite catalyst displayed overall water‐splitting along with stability of 72 h. In another report, Sang et al.^[^ ^107^ ^]^ developed CoNi‐LDH on ZIF‐derived cobalt nanoparticles and nitrogen‐doped carbon framework (CoNi‐LDH/Co@NC) as a cost‐effective bifunctional catalyst toward water‐splitting investigations. Surface morphology revealed the dodecahedral structure, which is characteristic of ZIF‐67, decorated with nanosheets of CoNi‐LDH, grown on nitrogen‐doped carbon. Co@NC offers a porous structure with a large specific surface area. While CoNi‐LDH furnishes high conductivity to enhance the reaction activity. Their synergistic effect results in outstanding electrocatalytic performance. The composite catalyst exhibited low η_100_: 187 mV and high η_100_: 359 mV for HER and OER, respectively. It is worth mentioning that the composite catalyst delivered a cell voltage of 1.51 V when used as both anode and cathode. Furthermore, CoNi‐LDH/Co@NC retained stable activity even after 20 h.

**Figure 11:**
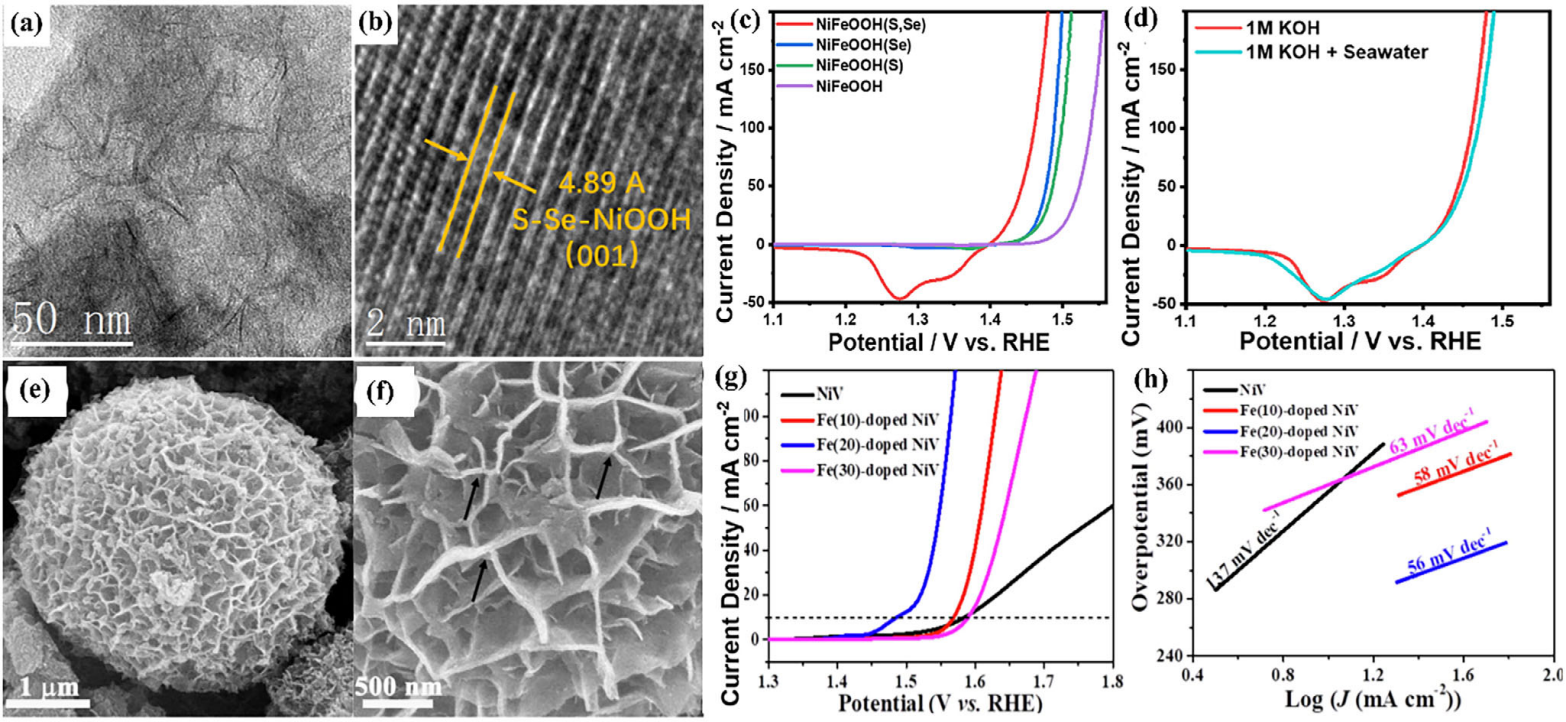
a,b) Surface morphology of Rh^3+^‐NiFeOOH(S, Se), c) its OER performance in alkaline KOH and d) OER performance in 1 M KOH with seawater, e, f) Surface morphology of Fe‐doped NiV HMS, g) LSV polarization curve and h) Corresponding Tafel slope of Fe‐doped NiV HMS. Reproduced from ref. [108] and ref. [109] with permission of Elsevier.

Zhao et al.^[^ ^108^ ^]^ designed NiFeOOH from nickel iron‐sulfide selenide surface to enhance the active sites, verified via TEM analysis in **Figure** **11**a,b. NiFeOOH(S, Se) demonstrated outstanding OER performance with low η: 195 mV and an impressively low Tafel slope of ≈31.99 mV dec^−1^ in alkaline KOH. As‐prepared catalyst remarkably provided moderate η_100_: 231 mV in KOH and 239 mV in KOH + seawater solution. The corresponding LSV polarization curves are shown in Figure 11c,d. The activity is credited to reactive sites formed during surface reconstruction. Furthermore, there was no significant change in overpotential even after 4 days of continuous testing in KOH + seawater solution was noticed, ensuring the stability of the catalyst for commercial purposes. In another investigation, Deng et al.^[^ ^109^ ^]^ carried out facile cation‐exchange methods to fabricate iron‐doped nickel‐vanadium hydroxide microspheres (Fe‐doped NiV HMS). The SEM images in Figure 11e,f revealed vertically aligned nanosheets intersecting and interpenetrating, forming intricate 3D porous flower‐like nanostructures. This hierarchical porosity significantly increases the number of active sites and improves the diffusion rate of electrolyte ions and electrons at the interfaces. For hierarchical Fe‐doped NiV HMS, moderate η: 255 mV and high η_50_:314 mV for OER, outperformed hierarchical NiV HMS, which has high η: 354 mV and η_50_:524 mV under the same conditions. Additionally, hierarchical Fe‐doped NiV HMS exhibited Tafel slope of just 56 mV dec^−1^, indicative of a superior OER catalytic rate in an alkaline environment. The associated LSV curve and Tafel slopes are attached in Figure 11g,h. Similarly, Karthick et al.^[^ ^110^ ^]^ synthesized nickel iron carbonate hydroxide hydrate (NiFeCHH) nanosheets to evaluate water‐splitting performance. The innovative approach of forming trimetallic RhNiFe carbonate hydroxide via incorporation of Rh^3+^ ions into NiFe system significantly enhances the catalytic performance. Rh^3+^‐NiFeCHH (1:0.5) showed moderate OER η: 207 mV and Tafel slope of 30 mV dec^−1^, while attaining an ultra‐low η: 36 mV for HER, boasting a Tafel slope of 34 mV dec^−1^. The catalyst on the anode and cathode for overall water‐splitting resulted in an η_50_: 286 mV, demonstrating outstanding potential for efficient energy conversion. The enhanced activity is credited to the layered structure of Rh^3+^‐NiFeCHH.

The capability of hydrides/hydroxides toward water‐splitting is recorded in Table 3 and Figure 9b. These materials have gained much importance in recent times due to their ability to achieve lower overpotentials and Tafel slopes. Despite their promising activity, especially in alkaline conditions, hydroxides and hydrides suffer from several intrinsic limitations. Their inherently poor electronic conductivity restricts efficient electron transfer to active sites, thereby diminishing catalytic current unless coupled with conductive supports such as carbon‐based materials.^[^ ^111^ ^]^ In HER, many hydroxide‐based catalysts display less favorable hydrogen adsorption energies and sluggish kinetics, requiring high overpotentials to reach industrially relevant current densities, with noticeable performance drop‐offs at elevated currents (e.g., 1000 mA cm^−2^).^[^ ^112^ ^]^ Structural transformation, surface oxidation, and active phase reconstruction during electrocatalysis further complicate the mechanistic understanding and compromise long‐term stability.^[^ ^111^ ^]^ Among hydroxides, LDHs have received considerable interest but remain challenging due to morphology‐dependent limitations that reduce the exposure of active sites. The catalytic activity of LDHs can be enhanced through cationic doping, though such modifications may significantly alter both morphology and reaction kinetics. Interestingly, tuning the interlayer spacing in LDHs has been shown to improve ion transport and accessibility of active sites, thereby enabling ultra‐low overpotential operation.^[^ ^106^ ^]^ Nevertheless, substantial research is still required to ensure operational stability, especially under high‐current regimes. Moving forward, integrating hydrides and hydroxides with conductive carbon‐based materials offers a promising route to overcome these challenges by enhancing charge transport, improving mechanical durability, and achieving synergistic effects in overall water‐splitting catalysis.

**Table 3:** Hydrides/hydroxides‐based catalysts for water electrolysis.

Electrocatalyst	Synthesis	Morphology	Electrolyte	Hydrogen Evolution		Oxygen Evolution		Refs.
				η [mV]	Tafel Slope [mV dec^−1^]	η [mV]	Tafel Slope [mV dec^−1^]	
NiFe‐LDH	Co‐Precipitation	Nanosheet	KOH	–	–	413	56	[113]
CoFe‐LDH	HT	Nanowires	KOH	–	–	252	61	[103]
CoFe‐LDHs	HT	Nanosheets	KOH	–	–	278	49	[102]
CoFeCr LDH	HT	Nanosheets	KOH	–	–	278	48	[104]
NiFe‐LDHs	Liquid Phase Method	Nanosheets	KOH	–	–	1440	25.85	[114]
Te‐NiCo LDHs	Metalloid Incorporation	Nanosheet	KOH	–	–	190	23.38	[115]
Ir‐NiCo LDH	ST	Nanosheet	KOH	21	33.2	192	41.2	[105]
CuS/NiFe‐LDH	HT	Nanosheet	KOH	55	33	170	91	[106]
Mo‐CoFe LDH	HT	Nanosheets	KOH	227 η_100_	94	331 η_100_	101	[116]
CoNi‐LDH	HT Method	Nanosheets	KOH	187 η_100_	94	359 η_100_	57	[107]
Ni(OH)_2_/Ni	Electrodeposition Method	Nanosheets	KOH	192 η_100_	129	564 η_100_	336	[117]
NiTe/FeOOH	HT Method	Nanoarrays	KOH	282 η_100_	99	280 η_100_	52	[118]
Ni/Ni (OH)_2_	Partial Reduction Strategy	Nanosheet	KOH	77	53	270	70	[119]
V‐Ce/CoFe LDH	HT Method	Nanosheet	KOH	73	66	–	–	[120]
Ir‐Ni (OH)_2_	Precipitation Method	Nanosheet	KOH	–	–	235	58.4	[121]
S,Se‐NiFe‐OOH	Chemical	Nanosheets	KOH	–	–	195	31.99	[108]
S‐CoFe‐OOH	Activation Method	Nanosheets	KOH	–	–	270	42.23	[122]
Ni‐Fe‐OOH	HT	Nanosheets	KOH	168 η_100_	102	224 η_100_	22.8	[123]
S‐NiFe‐OOH	HT	Nanosheets	KOH	176	90	220	40	[124]
Al‐OOH	Ball Milling	Nanosheets	KOH	–	–	400	52	[125]
FeNi‐OH	HT	Worm‐Like Structure	KOH	196 η_50_	88.8	236 η_50_	48.43	[126]
NiCo‐LDH‐OH	HT Method	Nanosheets	KOH	180	172.85	317	92.17	[127]
NiCo/NiCo‐OH and NiFe/NiFe‐OH	Electrodeposition and electrochemical oxidation method	Nanosheets	KOH	184 η_500_	42	296 η_500_	41	[128]
NiFeCHH	HT Method	Nanosheets	KOH	36 η_50_	34	207 η_50_	30	[110]
NiFe(OH)_2_	ST Ion‐Assisted	Nanosheets	KOH	–	–	259 η_1000_	67	[129]
Fe‐NiV‐OH	Cation‐exchange method	Microspheres	KOH	–	–	255	56	[109]
FeNi(OH)_2_/Fe‐LDHs	HT	Nanosheets	KOH	116	95.87	292 η_50_	121.37	[130]
Co(OH)_2_	HT	3D Flakes	KOH	150	64	230	43	[131]
CoFe‐OH/FeOOH	Wet chemical	Nanosheets	KOH	145	51	200 η_50_	48	[132]

### 3.4 Carbon‐Based Electrocatalysts

**Figure 12:**
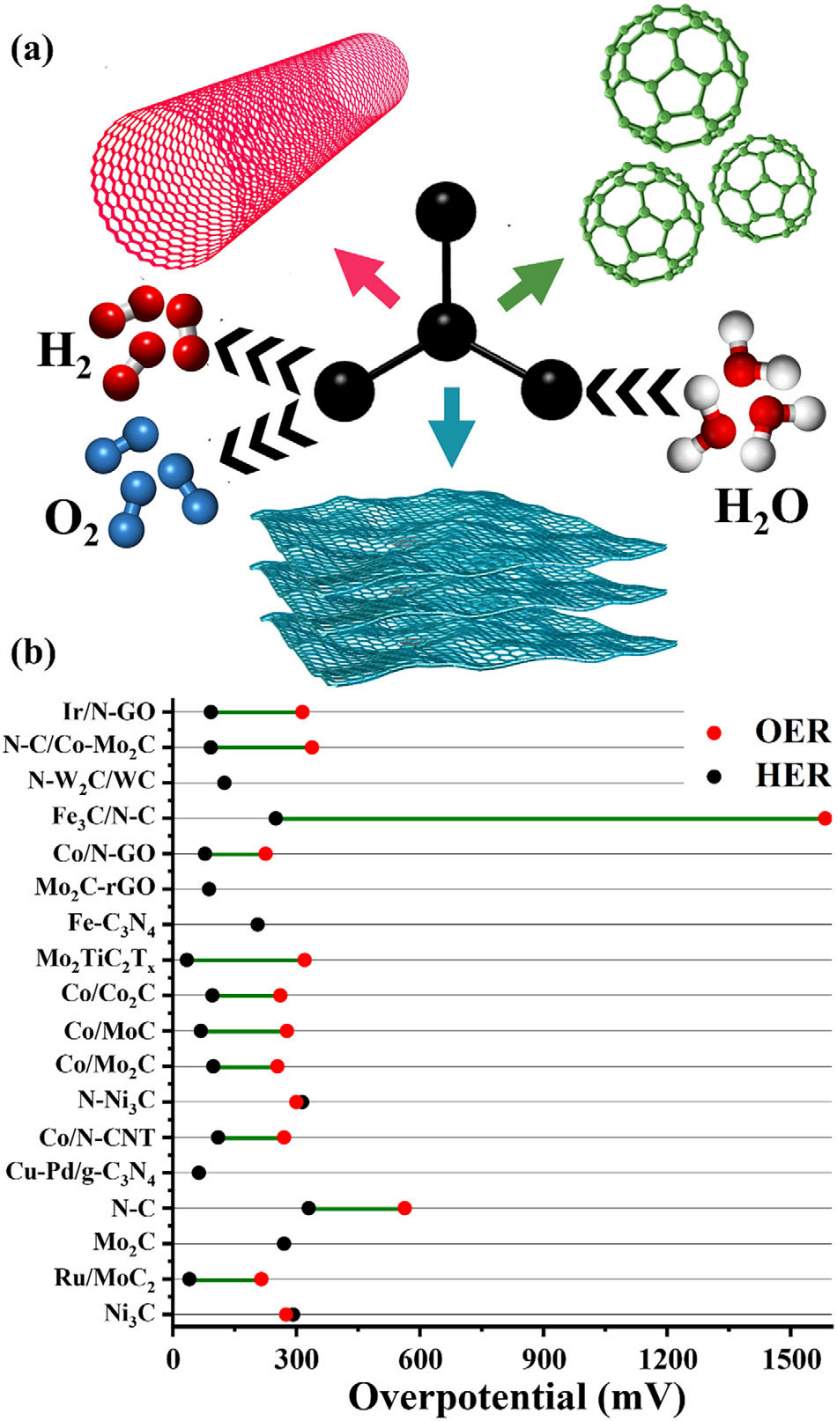
a) Carbon‐based materials, including carbon nanotubes, graphene sheets, and carbon buckyballs, highlight their potential as efficient electrocatalysts for HER and OER, and b) Intercomparison of η for HER and OER of carbon‐based materials on the basis of **Table** **4**.

Carbon‐based materials do not segregate and demonstrate enhanced electrocatalytic activity while being cost‐effective and thus exhibit significant potential in the field of electrocatalysis. Carbonaceous materials are easily tunable, possessing excellent thermal and electrical conductivity, substantial mechanical strength, symmetric structures, and high surface areas with unique morphologies. The regular arrangement of molecules produces symmetrical structures ranging from zero‐dimensional (0D) to three‐dimensional (3D), greatly impacting the porosity and active sites, beneficial for electrocatalysis, as in **Figure** **12**a.^[^ ^133^ ^]^ Another method employed to enhance their activity is the doping of heteroatoms; introducing impurities of varying atomic radii and electronegativities creates unique charge distributions, leading to modified properties.^[^ ^134^, ^135^ ^]^

Brief details of carbon‐based materials, including carbides, heteroatom doping in carbon architecture, and composites for enhanced electrocatalysis, are given below. Fan et al.^[^ ^136^ ^]^ synthesized Ni_3_C nanosheets doped with 2 at% of Fe to assess water‐splitting efficacy. As‐synthesized catalyst exhibited moderate η: 292 mV and Tafel slope of 41.3 mV dec^−1^ toward HER in KOH solution. Additionally, exceptional catalytic performance for OER was characterized by moderate η: 275 mV and Tafel slope of 62 mV dec^−1^ in alkaline KOH. The catalyst was further found stable even after 1000 cycles. Similarly, Zhao et al.^[^ ^134^ ^]^ synthesized Ru/Mo_2_C to control the valence states of both Ru and Mo within the catalyst, verified by HRTEM investigations. This led to improvement HER and OER in acidic electrolyte, reducing to ultra‐low η: 40 mV with Tafel slope of 26.8 mV dec^−1^ and moderate η: 215 mV with Tafel slope of 82.7 mV dec^−1^, respectively. The catalyst demonstrated significant potential for applications in overall water‐splitting, requiring merely 1.51 V of applied voltage and maintaining excellent stability for 12 h. Moreover, Chaitoglou et al.^[^ ^137^ ^]^ utilized chemical vapor deposition (CVD) to fabricate a vertically stacked bilayer graphene/Mo_2_C heterostructure. This configuration demonstrates exceptional stability, attributed to the strong interconnectivity of carbide nanostructures on the surface and elimination of polymeric binders during the electrode preparation process, enabled by its interconnected nanostructure. The heterostructure exhibited a Tafel slope of 56 mV dec^−1^ and moderate η: of 270 mV for HER in acidic electrolyte, approaching the performance of commercially available Pt‐catalysts. Graphene/Mo_2_C further illustrated outstanding durability over 1000 cycles.

**Figure 13:**
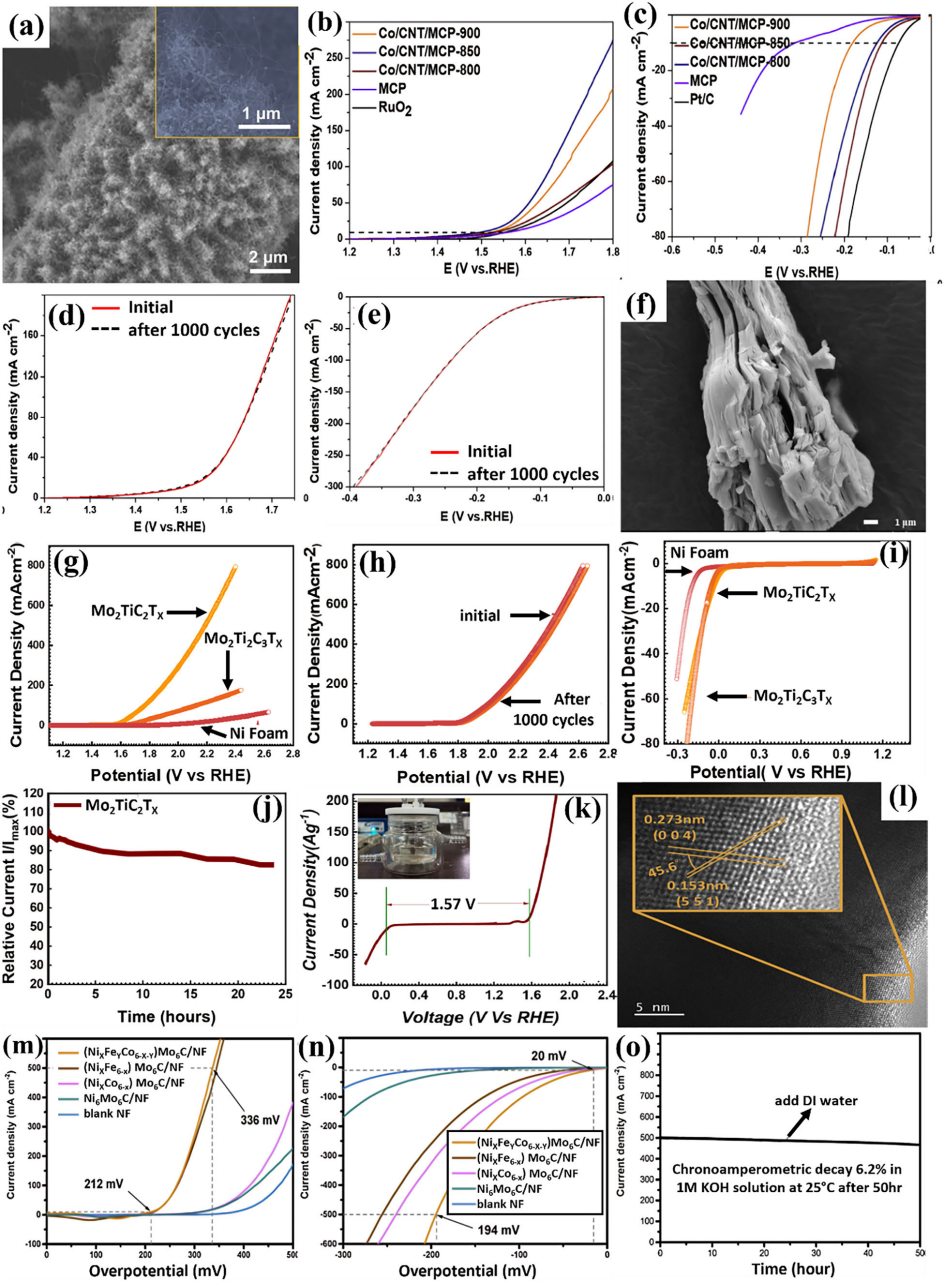
a) SEM image of Co/CNT/MCP‐850, b,c) LSV curves for OER and HER of Co/CNT/MCP‐850, d,e) Comparison of LSV curves for Co/CNT/MCP‐850 before and after 1000 cycles for OER and HER, f) Morphological analysis of Co/CNT/MCP‐850, g,i) LSV polarization curves for OER and HER h) Stability test over 1000 cycles, j) 24‐h chronoamperometric curve of Co/CNT/MCP‐850, k) LSV curve for overall water splitting of Mo_2_TiC_2_Tx, l) TEM image of (Ni_x_Fe_γ_Co_6−x−γ_)Mo_6_C, m,n) LSV curves for OER and HER and o) Chronoamperometric stability test over 50 h of (Ni_x_Fe_γ_Co_6−x−γ_)Mo_6_C. Reproduced from,^[^ ^133^, ^138^, ^139^ ^]^ with the permission of Elsevier.

Zhou et al.^[^ ^138^ ^]^ developed an effective approach to fabricate multifunctional self‐supporting electrocatalysts featuring uniformly distributed cobalt nanoparticles and clusters onto nitrogen‐doped carbon nanotubes to prevent agglomeration and corrosion issues of metal‐based electrocatalysts, the SEM image is shown in **Figure** **13**a. Leveraging excellent electrical conductivity and multiple active sites, the resulting trifunctional self‐supporting catalyst, resistant to stacking and aggregation, demonstrated outstanding performance in alkaline media. Specifically, it achieved impressive OER and HER activities with moderate η: 270 mV and low η: 110 mV, respectively, in 1 m KOH (Figure 13b,c). The catalyst was found to be stable for 1000 cycles (Figure 13d,e), paving the way for the design of highly efficient and multifunctional self‐supporting electrocatalysts for electrochemical energy conversion technologies. MXenes, noted for their rich surface chemistry, unique physiochemical properties, and stability, are also gaining prominence as highly efficient carbon‐based catalysts for green hydrogen production. In this regard, Zahra et al.^[^ ^133^ ^]^ successfully synthesized Mo_2_TiC_2_T_x_, a dual transition metal carbide, as a non‐precious metal‐based bifunctional catalyst for effective overall water‐splitting in alkaline conditions (1 m KOH). The surface visualization is provided in Figure 13f. Using LSV analysis, the catalyst exhibited an ultra‐low η: 34 mV for HER along with stability for extended time (Figure 13i,j). However, Mo_2_TiC_2_T_x_ required high η: of 320 mV for OER and remained stable even after 1000 cycles, as presented in Figure 13g,h. While Mo_2_TiC_2_T_x_ presented a cell voltage of 1.57 V for overall water‐splitting in Figure 13k. Similarly, He et al.^[^ ^139^ ^]^ synthesized a volcano‐like structure of (Ni_x_Fe_y_Co_6‐x‐y_)Mo_6_C via HT method, verified by HRTEM investigations in Figure 13l. As‐synthesized material achieved an ultra‐low η:20 mV for HER with a Tafel slope of 47.4 mV dec^−1^ and moderate η: 212 mV for OER with Tafel slope of 60 mV dec^−1^ in 1 m KOH. The associated LSV polarization curves are presented in Figure 13m,n. The catalyst further demonstrated chronoamperometric decay of only 6.2% despite operation at a high initial **J** of 500 mA cm^−2^ for 50 h in Figure 13o.

Table 4 summarizes the carbon‐based catalysts that have been reported for water‐splitting in the last 5 years and is visually presented in Figure 12b. Carbonaceous materials, including multiwalled‐CNTs and reduced‐GO, are considered to be effective candidates for HER and OER electrocatalysis as a result of their excellent electrical conductivity, chemical stability, and abundance of surface active sites. These materials exhibit acceptable overpotential values for both reactions, although their Tafel slopes tend to display suboptimal intrinsic catalytic kinetics. Notably, different carbon morphologies also function well as support platforms for embedding active electrocatalytic species, optimizing overall performance through synergistic effects. Nevertheless, there are challenges. Under OER conditions and especially when in alkaline environments, carbon supports tend to oxidize to CO_2_ or carbonates, causing structural damage and compromised long‐term stability.^[^ ^140^ ^]^ Moreover, graphene sheets are prone to restacking through van der Waals interactions, which restrict available surface area, whereas CNTs tend to aggregate and have poor dispersibility owing to their chemically inert nature.^[^ ^141^ ^]^ Heteroatom doping (e.g., N, B, P, S) and incorporation of structural defects are methods to enhance catalytic performance, but doping may disrupt the sp^2^ carbon network, increase the bandgap, and decrease electrical conductivity.^[^ ^142^ ^]^ On the other hand, functionalization and synthesis of carbon materials also entail intricate and occasionally expensive procedures, so scalability becomes an issue.^[^ ^143^ ^]^ In spite of the above restrictions, defect‐rich rGO and doped carbon nanostructures are extremely promising for improving electrocatalytic activity, both theoretically (by the use of DFT modeling) and experimentally. Future research needs to involve in situ/operando characterization in order to get a more dynamic insight into the role of carbon materials in catalysis, along with computational modeling toward optimizing their electronic features and active species interactions. In general, whereas carbon‐based materials tend to function more as conductive scaffolds than actual catalysts, their superior electron transfer ability, mechanical stability, and low cost make them extremely useful components in large‐scale, composite electrocatalyst systems.

**Table 4:** Carbon‐based catalysts for water electrolysis.

Electrocatalyst	Synthesis method	Morphology	Electrolyte	HER		OER		Refs.
				η [mV]	Tafel slope [mV dec^−1^]	η [mV]	Tafel slope [mV dec^−1^]	
Ni_3_C	Carburization	Nanosheets	KOH	292	41.3	275	62	[144]
Ru/MoC_2_	Pyrolysis	Lallemar Structure	H_2_SO_4_	40	26.8	215	82.7	[145]
Mo_2_C	CVD	Nanocrystals	H_2_SO_4_	270	56	–	–	[146]
Fe_3_C/Mn_3_(BO_3_)_2_	Electrospinning	Nanofibers	KOH	–	88.82	–	57.70	[147]
N‐C sheets	Carbonization	Nanosheets	KOH	330	420	563	71	[148]
Cu‐Pd/g‐C_3_N_4_	Impregnation Method	Nanoparticles	H_2_SO_4_	63	42	–	–	[149]
Co/N‐CNT	In Situ Approach	Bamboo Shape	KOH	110	79	270	79	[138]
N‐Ni_3_C	Co‐Precipitation	Nanosheets	KOH	314	91	300 η_100_	83	[144]
Co/Mo_2_C	Segregation	Irregular Nanoparticles	KOH	98	68	254	123	[150]
Co/MoC	Solution‐Phase Precipitation	Core shell	KOH	68	87	277	79	[151]
Co/Co_2_C	HT Method	Nanowires	KOH	96	82.2	261	58.8	[152]
Mo_2_TiC_2_Tx	Wet Chemical	Nanosheets	KOH	34	30	320	86	[153]
Fe‐C_3_N_4_	Pyrolysis	Mesoporous	KOH	206	82	–	–	[154]
Mo_2_C‐rGO	Wet Chemical	Nanocrystals	H_2_SO_4_	88	67	–	–	[155]
Co/N‐GO	HT	Nanosheets	KOH	78	105	225	51	[156]
Fe_3_C/N‐C	Pyrolysis	Micro‐Sized Particle	KOH	250	67.39	1584	54.48	[157]
N‐W_2_C/WC	Pyrolysis	Flaky Nanoparticles	KOH	125	62.66	–	–	[158]
N‐C/Co‐Mo_2_C	Carbonation	Nanosheets	KOH	92	89	338	73	[159]
F‐CNTs	SST	Nanotubes	KOH	–	–	25 η_15_	75	[160]
Ir/N‐Graphene	HT	Nanosheets	KOH	92.5	41	314.6	74	[161]
Ni_x_Fe_y_Co_6‐x‐y_/Mo_6_C	HT Method	Cuboid	KOH	20	47.4	212 η_500_	60	[162]

### 3.5 Phosphides

**Figure 14:**
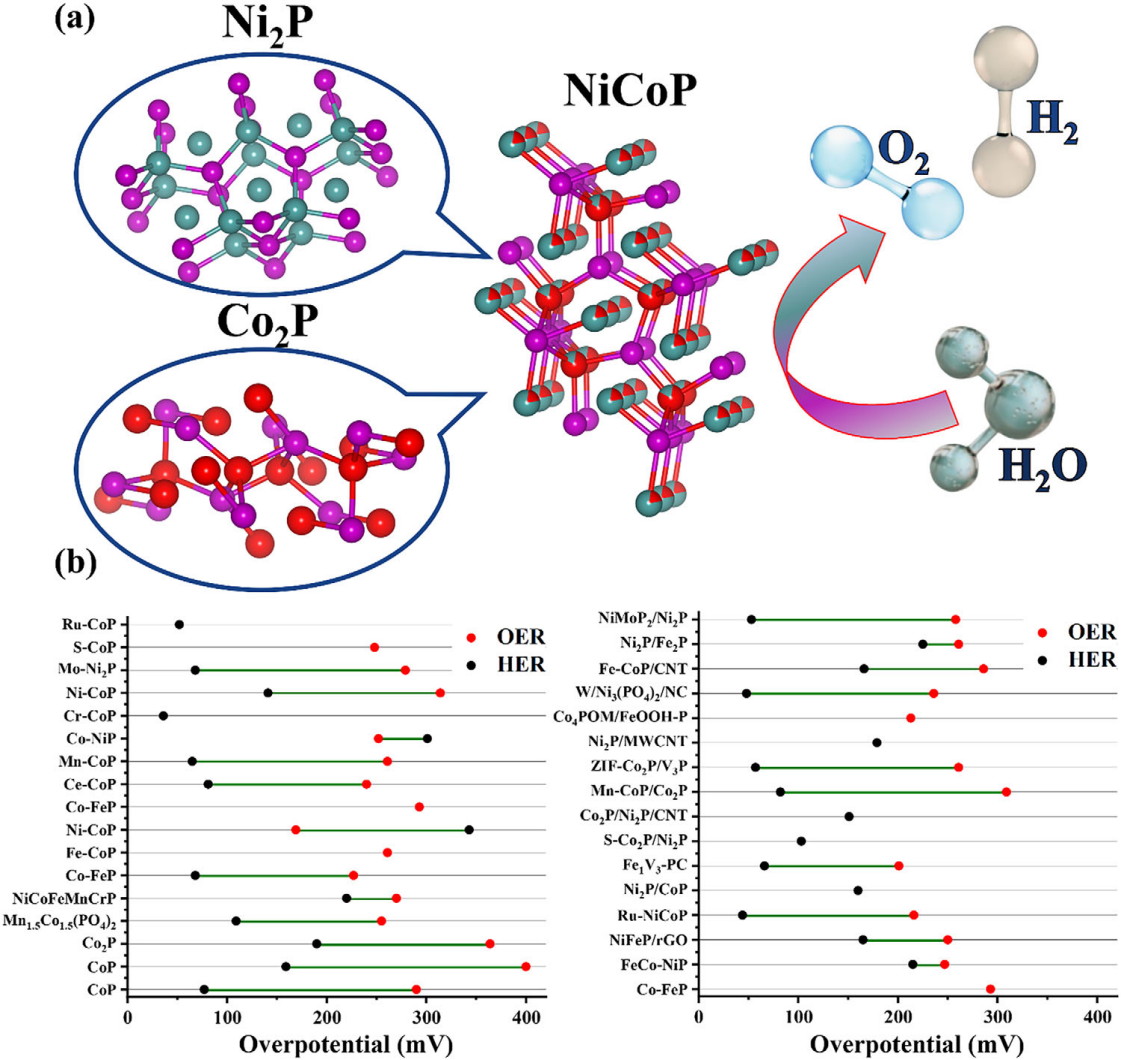
a) The unique structure of nickel cobalt phosphide is illustrated, highlighting the roles of cobalt phosphide (Co_2_P) and nickel phosphide (Ni_2_P) as efficient components for electrocatalytic water‐splitting and b) Intercomparison of η for HER and OER activity of phosphides, based on **Table** **5**.

Phosphides are highly regarded for their longevity and stability and are extensively studied as bifunctional catalysts for HER and OER. Phosphides act as proton acceptors, and due to the availability of proton acceptor sites, they help to increase OER efficiency. Furthermore, they can produce HER and OER results comparable to noble metals.^[^ ^163^, ^164^, ^165^ ^]^ The schematic representation of their structure is provided in **Figure** **14**a, and details of some phosphides as water‐splitting catalysts are given below.

**Figure 15:**
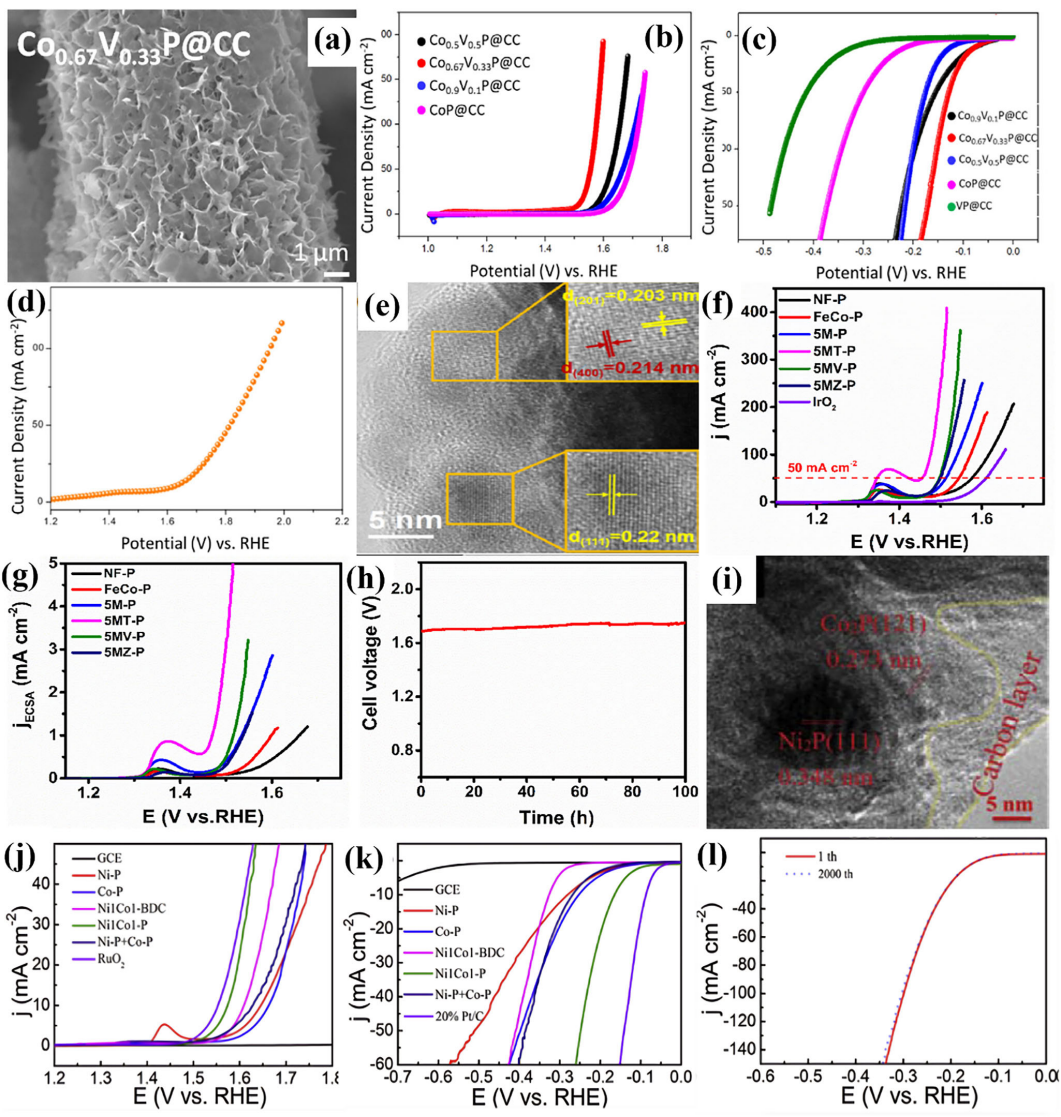
a) FESEM image of CoVP@CC, b,c) LSV polarization curves for OER and HER of CoVP@CC, d) LSV polarization curve for CoVP@CC//CoVP@CC in a two‐electrode system, e) HRTEM image of 5MT‐P, f,g) LSV curves for OER and HER of 5MT‐P, h) Stability test at 100 mV cm^−2^ for 100 h of 5MT‐P, i) TEM image of 5MT‐P, j,k) Polarization curves for OER and HER of 5MT‐P, l) Polarization curve of the first 2000 CV sweeps of 5MT‐P. Reproduced from,^[^ ^163^, ^166^, ^167^ ^]^ with permission of Elsevier.

Han et al.^[^ ^166^ ^]^ synthesized vanadium‐incorporated cobalt phosphide (CoVP) self‐supported catalysts on carbon cloth (CC) (CoVP@CC) via a wet chemical technique. V controls the self‐supported CoP nanosheets' surface electronic structure in CoVP@CC, which imparts superior catalytic activity. The SEM image of CoVP@CC demonstrates the combination of nanosheets and nanoparticles in **Figure** **15**a. For OER and HER, Co_0.67_V_0.33_P@CC had moderate η: 290 and ultra‐low η: 77 mV, respectively, in alkaline media (Figure 15b,c). The estimated Tafel slopes for HER and OER were 37.8 and 64.9 mV dec^−1^, respectively. Additionally, overall water‐splitting was achieved at a remarkably low potential of 1.61 V, evident from Figure 15d. According to Nguyen et al.,^[^ ^163^ ^]^ adding Ti to quinary‐metal phosphide (5MT‐P) exhibits the greatest performance, providing exceptional durability and bi‐functional catalytic activity in alkaline media. 5MT‐P with amorphous structure and solid spherical morphology (Figure 15e) needs moderate η_50_: 226 mV, with a Tafel slope of 35.3 mV dec^−1^ for OER, also shown in Figure 15f,g. With a Tafel slope of 92 mV dec^−1^ and moderate η: 220 mV, 5MT‐P exhibits HER activity. Also, an electrolytic cell with 5MT‐P||5MT‐P demonstrated good durability for 100‐h operation in Figure 15h and recorded a low cell voltage of 1.69 V at 100 mA cm^−2^. Similarly, Shuai et al.^[^ ^167^ ^]^ prepared NiCo bimetallic phosphides (NiCo‐P) by one‐step phosphorization of NiCo, combining the synergistic effect of active sites of metal and superior conductivities of bimetallic phosphides. NiCo‐P nanostructures are shown in Figure 15i. NiCo‐P demonstrated exceptional electrocatalytic activity for both OER and HER, with high η: 343 mV for OER and low η: 169 mV for HER in alkaline conditions, displayed in Figure 15j,k. NiCo‐P also exhibited durability for HER even after 2000 cycles in Figure 15l.

Rare‐earth elements have emerged as essential catalysts for managing transition metal electrocatalysis. Li et al.^[^ ^168^ ^]^ created an array‐like Ce‐CoP catalyst to investigate HER and OER. Ce‐CoP@CC possessing a nanoneedle‐arrays morphology illustrated ultra‐low η: of 81 and η:240 mV with Tafel slopes of 68.7 and 50.39 mV dec^−1^ for HER and OER, respectively.

Another interesting method for creating effective water‐splitting catalysts with increased surface area and catalytic activity is the direct growth of metal phosphide nanowires on porous electrodes. Using cation exchange and phosphorization, Roh et al.^[^ ^169^ ^]^ created different metal (Fe, Mo, V, and Co)‐doped Ni_2_P nanowires (NiMP‐NWs) on NF. Fe‐doped Ni_2_P‐NWs exhibited the highest OER performance, with a Tafel slope of 34 mV dec^−1^ and a moderate η_100_: 279 mV in alkaline conditions. Mo‐doped Ni_2_P‐NWs exhibited excellent performance toward HER activity, with a Tafel slope of 87 mV dec^−1^ and ultra‐low η: 68 mV. The catalyst displayed excellent HER performance even after 5000 cycles. A low cell voltage, i.e., 1.57 V was needed for overall water‐splitting, combining NiMoP/NiFeP as the anode and cathode to obtain **J**_10_. NiCoP stands out due to its unique electrical properties, which facilitate efficient charge transfer. However, its OER performance is frequently inadequate. Ma et al.^[^ ^170^ ^]^ developed Mn‐doped nickel cobalt phosphide (Mn‐NiCoP) nano pin arrays by in‐situ growth on NF, to solve this issue. Mn‐NiCoP attained moderate η_100_: 266 mV for OER, having Tafel slopes of 53 mV dec^−1^. Mn‐NiCoP further demonstrated efficient water‐splitting performance when used as both HER/OER electrodes, achieving a voltage of only 1.69 V at a high **J** of 100 mA cm^−2^. Furthermore, it maintained this performance at 94% efficiency over a period of 240 h. Similarly, Zhang et al.^[^ ^171^ ^]^ used a simple HT method to create a unique Er‐doped NiCoP nanowire array supported on Ni foam (Er‐NiCoP/NF) for the first time, followed by phosphorization, confirmed via HRTEM. The developed electrode was notable for its remarkable bifunctional catalytic activity. In alkaline conditions, it needed low η: 46 mV and moderate η: 225 mV for OER and HER, respectively. The stability of the catalyst toward HER and OER after 5000 cycles is also evidence of its commercialization potential. In the same manner, trimetallic phosphide (Fe–Co–Ni–P) was synthesized by Zhang et al.^[^ ^172^ ^]^ by coating Fe–Co Prussian blue analogue (PBA) with Ni‐Co PBA. OER and HER with moderate η: 247 mV and η: 215 mV, respectively, were noticed when Fe−Co−Ni−P was employed as an electrocatalyst. In addition, Fe−Co−Ni−P demonstrated improved long‐term stability for OER and HER and faster kinetics due to abundant active sites.

The metal hydroxide/hydroxyl oxide produced by oxidation of TM phosphide offers an active site for OER, whereas TM phosphide serves as the active site for the catalysis of HER. The formation of active sites is hypothesized to be facilitated by the synergistic interaction of Fe, Co, and Ni, which collectively modulate the electronic structure to enhance catalytic activity. Chang et al.^[^ ^173^ ^]^ synthesized bimetal Prussian blue analogue (PBA) precursors integrated with graphene oxide (GO) to create phosphide‐rGO hybrid. In addition to having significant HER qualities, the hybrid undergoes partial surface oxidation to produce oxide layers to improve OER activities. NiFeP‐rGO combination showed moderate HER catalytic characteristics, reaching low η:165 mV and adequate stability, along with a moderate η: 250 mV for OER performance. NiFeP‐rGO‐based symmetric water electrolytic cell displayed an adequate input voltage value and high faradaic efficiency (FE) for O_2_ and H_2_ production because of the bifunctional catalytic characteristics. The catalyst was also found to be stable for 50 h.

**Figure 16:**
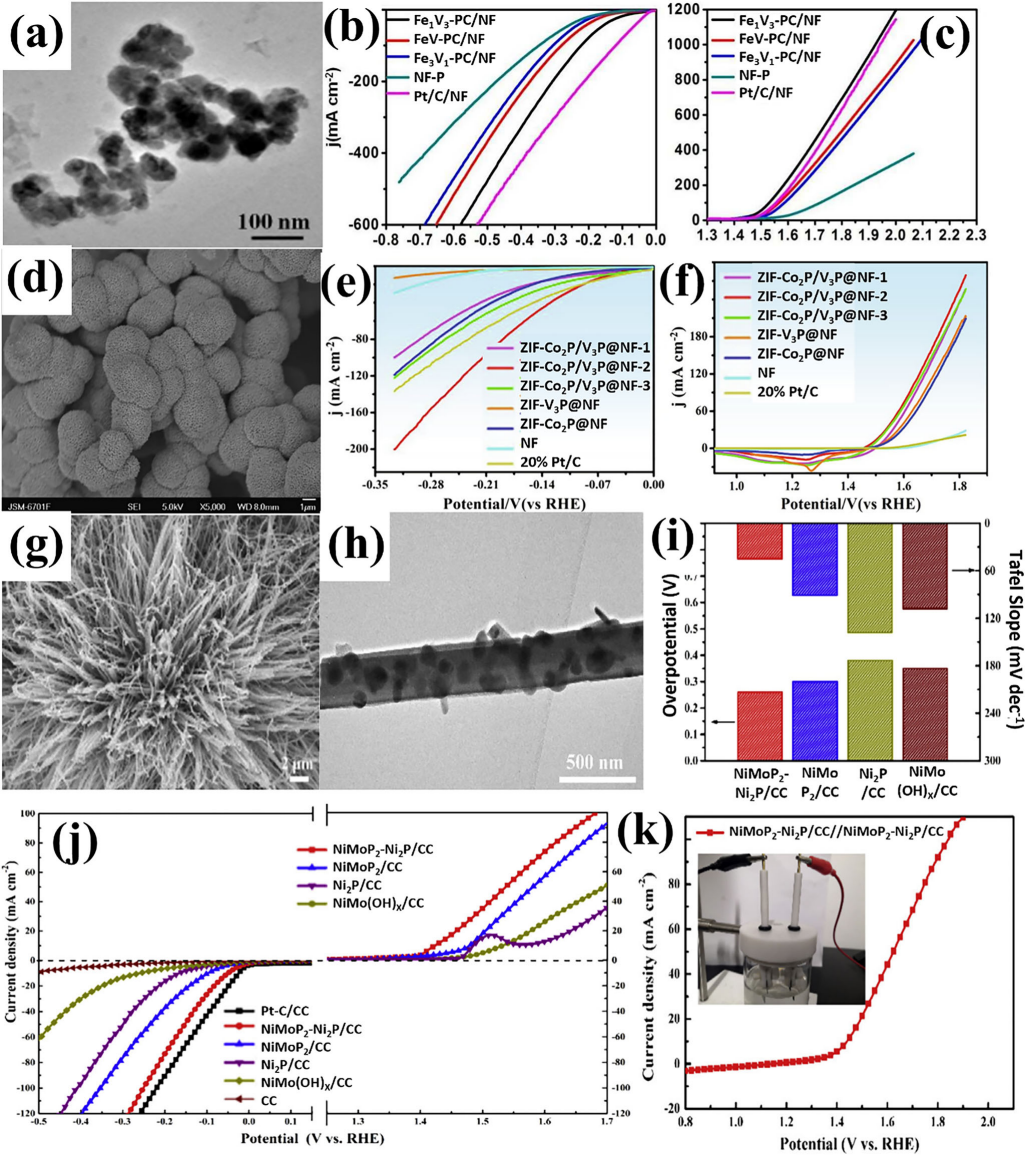
a) TEM image of Fe_1_V_3_‐PC/NF, b) HER performance of Fe_1_V_3_‐PC/NF, c) OER activity of Fe_1_V_3_‐PC/NF, d) SEM image of ZIF‐Co_2_P/V_3_P@NF, e) HER performance of ZIF‐Co_2_P/V_3_P@NF, f) OER activity of ZIF‐Co_2_P/V_3_P@NF, g,h) Surface morphology of NiMoP_2_‐NiP_2_/CC, i) Overpotential and Tafel slope values for HER and OER of NiMoP_2_‐NiP_2_/CC, j) LSV polarization curves for HER and OER of NiMoP_2_‐NiP_2_/CC, k) Overall water splitting using NiMoP_2_‐Ni_2_P/CC (+) // NiMoP_2_‐Ni_2_P/CC (−) couple. Reproduced from,^[^ ^174^, ^175^, ^176^ ^]^ with permission of Elsevier.

Suo et al.^[^ ^174^ ^]^ used an amalgamation of ST processes and phosphorization techniques to create Fe‐enriched vanadium phosphide customized porous carbon packing on NF (Fe_x_V_y_‐PC/NF) catalyst from the bimetallic Fe/V metal‐organic framework (Fe_x_V_y_‐MOF) for the first time. The modified electrocatalyst exhibited improved HER, OER, and overall water‐splitting performance since it inherits the advantages of MOF. The addition of Fe can be used to adjust the structure and conductance of vanadium phosphide; the morphology for Fe_1_V_3_‐PC/NF is displayed in **Figure** **16**a. In 1 m KOH, the electrocatalyst was impressively able to provide ultra‐low η: 66 mV and moderate η: 201 mV for HER and OER, respectively (Figure 16b,c). According to the findings from experiments, carbonation and phosphating were responsible for enhanced performance. In another study, by using a logical HT ‐coprecipitation‐phosphidation technique, Yang et al.^[^ ^175^ ^]^ skillfully created a new hierarchical dual‐functional electrocatalyst ZIF‐Co_2_P/V_3_P. The unique qualities of ZIF nanocrystalline with a large specific surface area, high porosity, and NF substrate with 3D interconnected porous structure, shown in Figure 16d, collectively impart excellent electrocatalytic activity. ZIF‐Co_2_P/V_3_P@NF exhibited both HER and OER with an ultra‐low η: 57 mV and moderate 261 mV in 1 m KOH solution, respectively, illustrated in Figure 16e,f. Meanwhile, at 25 mA cm^−2^, the electrocatalytic cell had outstanding persistence for at least 24 h with low degradation in performance.

Furthermore, Tian et al.^[^ ^176^ ^]^ provided a straightforward HT and phosphorization technique under H_2_/Ar (10/90) atmosphere for the fabrication of NiMoP_2_‐Ni_2_P/CC heterostructure. NiMoP_2_‐Ni_2_P/CC possessed an amorphous characteristics junction that can effectively decrease the energy required for hydrogen and oxygen desorption and may speed up electron transmission, corresponding morphology is exhibited in Figure 16g,h. Consequently, the heterostructure NiMoP_2_‐Ni_2_P/CC required moderate η: 258 mV and ultra‐low 53 mV for OER and HER, respectively. The associated LSV polarization curves, overpotential, and Tafel slope values are attached in Figure 16i,j. Interestingly, NiMoP_2_‐NiP_2_/CC for overall water‐splitting needed less voltage to operate, i.e., at 1.48 V at **J**_10_ in Figure 16k.

Transition metal phosphides (TMPs) are known for their high electrical conductivity and low water electrolysis overpotential. The documented phosphide‐based electrocatalysts from literature are provided in Table 5 and Figure 14b. Although TMPs exhibit superior activity, they are plagued by serious issues that make their utilization on a practical level impossible. Among the most significant problems is their susceptibility to oxidation under environmental and operating conditions, causing drastic degradation and reduced catalytic activity. In alkaline HER conditions, TMPs have a tendency to leach phosphorus atoms, particularly in amorphous forms, because P‐metal bonds are weaker there.^[^ ^177^ ^]^ Under oxidative OER conditions, these materials often experience surface reconstruction, changing into metal oxides or (oxy)hydroxides. This threatens intrinsic catalytic identity and durability over time, since the phosphide can be just a precatalyst.^[^ ^178^ ^]^ Moreover, TMPs have larger overpotentials and poorer activation kinetics in practical PEM electrolyzers than noble metal catalysts.^[^ ^179^ ^]^ There are also issues with synthetic routes, which may include toxic phosphine gas or the creation of environmentally unsafe byproducts such as white phosphorus.^[^ ^180^ ^]^ Under high current densities, intense gas evolution may lead to the physical removal of TMPs from the electrode surface with severe loss of active material.^[^ ^181^ ^]^ Methods of surface doping, defect engineering, and nanostructuring have been conceived to counteract these disadvantages, increase oxidation resistance, and enhance structural stability.

**Table 5:** Phosphides‐based catalysts for water electrolysis.

Electrocatalyst	Synthesis	Morphology	Electrolyte	Hydrogen evolution		Oxygen evolution		Refs.
				η [mV]	Tafel slope [mV dec^−1^]	η [mV]	Tafel slope [mV dec^−1^]	
NiP	Electroless plating	Nanoballs	KOH	208 η_200_	98.12	392 η_200_	101.12	[182]
CoP	Wet‐chemical route	Nanosheets	KOH	77	56	290	55.59	[183]
CoP	Microwave assisted ST	Spherical structure	KOH	220 η_50_	92	226 η_50_	35.3	[184]
CoP	MOFs	Polyhedral morphology	KOH	159	59	400	57	[185]
Co2P	ST	Nanoparticles	KOH	190	68	364	81	[186]
Mn_1.5_Co_1.5_(PO_4_)_2_	Electrodeposition	Nanowires	KOH	109	99	255	36	[187]
NiCoFeMnCrP	Sol–gel route	Nanoparticles	KOH	220	94.5	270	52.5	[188]
Co‐FeP	HT Method	Micro flowers	KOH	68	58.69	227	30.78	[189]
Fe‐CoP	Heat Treatment	Nano cubes	KOH	–	–	261	50	[190]
Ni‐CoP	Phosphorization	Nanosheets	KOH	343	68	169	77	[191]
Co‐FeP	ST Route	Spindle‐morphology	KOH	–	–	293	43.5	[192]
Ce‐CoP	Phosphorization	Nanoneedle	KOH	81	68.7	240	50.39	[193]
Mn‐CoP	Electrodeposition	Nanosheets	KOH	65	46.16	261	162.25	[194]
Co‐NiP	HT Method	Nanowires	KOH	301	–	252	54	[195]
Cr‐CoP	Phosphorization Method	Nanorods	KOH	36	54	–	–	[196]
Mo‐CoP	HT	Nanosheets	KOH	112 η_100_	69.1	329.9 η_100_	86.8	[197]
Ni‐CoP	ST	Nanoflower	KOH	141	68	314	71	[198]
Mo‐Ni2P	Phosphorization	Nanowires	KOH	68	87	279	34	[199]
Ni‐CoP	Molten salt method	Nanowires	KOH	159 η_400_	64.81	391 η_400_	89.68	[200]
S‐CoP	HT	Nano‐needle	KOH	–	–	248	63	[201]
Ru‐CoP	Phosphorization	Nano‐array	KOH	52	39.7	–	–	[202]
Mn‐NiCoP	HT Method	Nano pin arrays	KOH	148 η_100_	84	266 η_100_	53	[203]
Er‐NiCoP	HT Method	Nanowires	KOH	46	42	225 η_10_	26	[204]
Mo‐Ni2P	Thermal phosphating process	Nanosheet array	KOH	259 η_3000_	70.9	410 η_1000_	152.9	[205]
Co‐FeP	ST Route	Spindle‐like morphology	KOH	–	–	293	43.5	[192]
FeCo‐NiP	Phosphidation	Coreshell	KOH	215	72.1	247	45.5	[172]
NiFeP/rGO	Phosphorization Treatment	nano cubes	KOH	165	96	250	68.4	[173]
Ru‐NiCoP	Phosphating	Nanoflowers	KOH	44 η_20_	45.4	216	84.5	[206]
Ni_2_P/CoP	HT Method	Nanospheres	H_2_SO_4_	160	57	–	–	[207]
Fe_1_V_3_‐PC	Phosphorization approach	Nanoparticles	KOH	66	37	201	75	[174]
S‐Co_2_P/Ni_2_P	Ion‐exchange strategy	Nanowires	KOH	103 η_100_	58.7	–	–	[208]
Ni‐CoP/Co_2_P	Phosphating process	Nanosheets	KOH	170 η_100_	67.1	352 η_100_	93.5	[209]
Co_2_P/Ni_2_P/CNT	Phosphorization	Cubic structure	H_2_SO_4_ KOH	151 202	41.64 57.95	–	–	[210]
Mn‐CoP/Co_2_P	Phosphating Treatment	Nanotubes	KOH	82	86.84	309	103.27	[211]
ZIF‐Co_2_P/V_3_P	ST	Coreshell	KOH	57	65	261	67.3	[175]
Fe‐CoP/PN‐C	Carbonization treatment	Porous structure	KOH,	110	101	300 η_50_	91	[212]
Ni_2_P/MWCNT	HT	Nanoparticles	H_2_SO_4_	179	71	–	–	[213]
Co_4_POM/FeOOH‐P	HT	Nanotubes	KOH	–	–	213	47	[214]
W/Ni_3_(PO_4_)_2_/NC	Electrodeposition	Nanorods	KOH	48	59.8	236	157.28	[215]
Fe‐CoP/CNT	Co‐coordination synthesis	Nanotubes	KOH	166	57.1	286	39.6	[216]
Ni_2_P/Fe_2_P	Growth‐ion exchange‐phosphidation	Micro sheets	KOH	225	86	261	58	[217]
NiMoP_2_/Ni_2_P	HT	Nanowires	KOH	53	58	258	45	[176]

Expanding the discussion to chalcogenides, consisting of metals being bonded to chalcogen atoms such as sulfur, selenium, or tellurium, provides another highly promising family of electrocatalysts. The layered structure of these materials enhances ion diffusion and provides rich active sites for favorable exposure, enabling efficient charge transfer and high catalytic activity toward both HER and OER.

### 3.6 Chalcogenides

**Figure 17:**
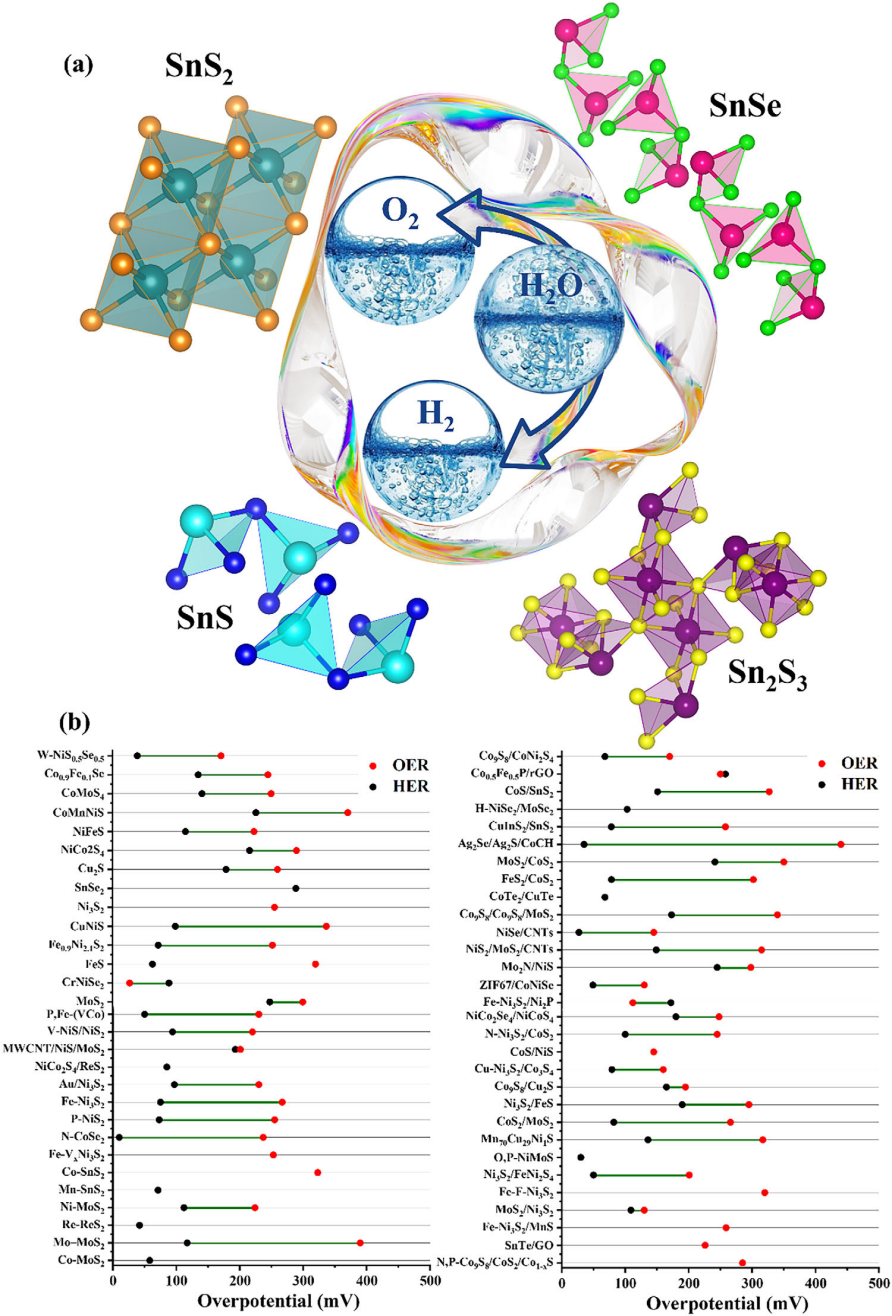
a) 2D layered structure of chalcogenides is illustrated, emphasizing the potential of tin sulfides for water dissociation and b) Intercomparison of η for HER and OER of chalcogenides, based on **Table** **6**.

Chalcogenides usually exhibit two‐dimensional (2D) morphologies and exceptional physicochemical characteristics. They can provide higher efficiency for HER and OER across a wide pH range owing to their electronic structure,^[^ ^218^ ^]^ as illustrated in **Figure** **17**a.

The electrocatalytic activity of some chalcogenides is given as follows:

Using the combined advantages of high electronic conductivity, porous structure, and enhanced sulfur adsorption on Cu(I), Pei et al.^[^ ^219^ ^]^ created a sulfur‐tolerable bifunctional Cu_2_S micro‐flake catalyst endorsed on NF (Cu_2_S/NF), to catalyze the electrochemical water‐splitting process. Cu_2_S/NF electrode demonstrated satisfactory activity and reliable HER at low η: 180 mV and high η_100_: 320 mV. Galvanostatic electrolysis further verified the electrode's long‐term stability for 48 h at 50 mA cm^2^. In an investigation, Song et al.^[^ ^220^ ^]^ created caterpillar‐like NiCo precursor arrays on CC (NiCo_2_S_4_/CC), using a simple HT procedure. Binary active metal sites within a single nanoparticle can provide tunable selectivity for various catalytic reactions. Furthermore, its distinctive caterpillar‐like hierarchical nanostructure exposes numerous active catalytic sites, enhancing the adsorption of electrolyte ions and facilitating the diffusion of reactants and products, thereby improving overall catalytic efficiency. NiCo_2_S_4_/CC thus exhibited electrocatalytic activity toward both HER and OER with moderate η: 216 mV and η: 290 mV, respectively. The overall water‐splitting was recorded with a cell voltage of 1.66 V, significantly better than that of its monometallic NiS/CC and Co_9_S_8_/CC counterparts. Likewise, using a ST process, Yin et al.^[^ ^221^ ^]^ constructed NiFeS hollow hierarchical sphere with better OER and HER activities than single‐phase NiS and FeS hollow hierarchical spheres, as well as the majority of bifunctional electrocatalysts when catalyzing water‐splitting. Ni–Fe–S hollow hierarchical sphere needed a moderate η: 223 mV for OER and low η: 115 mV for HER. As‐prepared electrocatalyst required overall cell voltage of 1.5 V to generate **J**_10_, which is equivalent to Pt||IrO_2_ performance.

**Figure 18:**
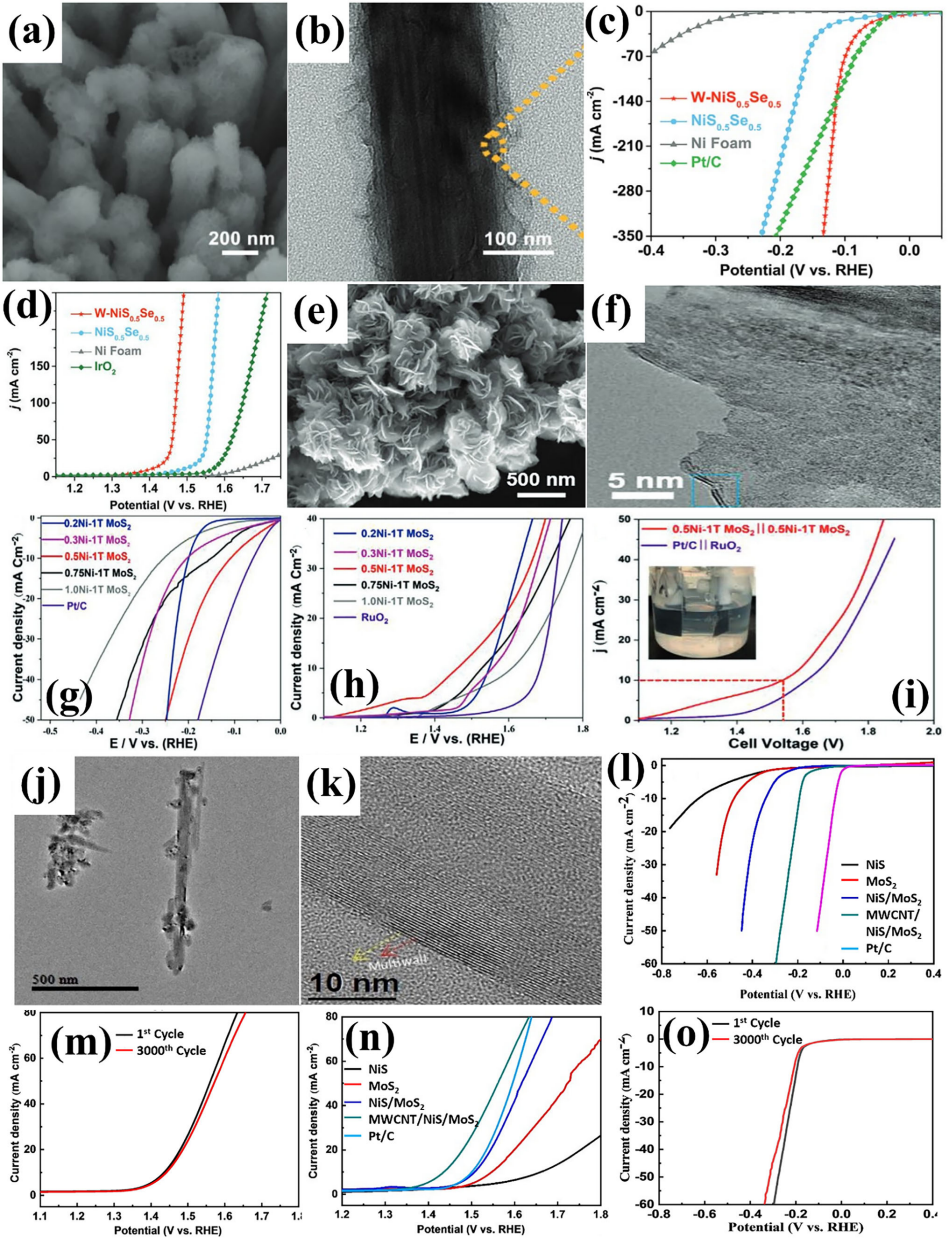
a) SEM micrograph of W‐NiS_0.5_Se_0.5_, b) TEM image of W‐NiS_0.5_Se_0.5_, c) HER performance of W‐NiS_0.5_Se_0.5_, d) OER performance of W‐NiS_0.5_Se_0.5_, e) SEM image of IT‐MoS_2_, f) TEM analysis of IT‐MoS_2_, g) HER activity of IT‐MoS_2_, h) OER performance of IT‐MoS_2_, i) Overall water splitting efficacy of IT‐MoS_2_ in 1 M KOH, j) TEM image of MWCNT/NiS/MoS_2_, k) HRTEM results of MWCNT/NiS/MoS_2_, l) HER performance of MWCNT/NiS/MoS_2_, m) OER activity of MWCNT/NiS/MoS_2_, n,o) Cyclic stability of MWCNT/NiS/MoS_2_ for 3000 cycles toward HER and OER. Reproduced from with permission of,^[^ ^218^, ^222^, ^223^ ^]^ with permission of Wiley.

The spinning state of Ni is delocalized upon the addition of single‐atom W, leading to an increase in electron density in d‐orbital of Ni.^[^ ^218^ ^]^ As a result, the adsorption of free energy for the rate‐determining step is significantly reduced, and the adsorption/desorption process is optimized. A single‐atom W‐doped NiS_0.5_Se_0.5_ nanosheet@NiS_0.5_Se_0.5_ nanorod heterostructure (W‐NiS_0.5_Se_0.5_) electrocatalyst was developed, with morphology is being presented in **Figure** **18**a,b. W‐NiS_0.5_Se_0.5_ illustrated catalytic activity for both HER and OER at ultra‐low η:39 and low η_100_:106 mV for HER and low η: 171 and moderate η_100_: 239 mV for OER respectively. associated LSV curves are attached in Figure 18c,d. Low potentials, i.e., 1.44 V and 1.55 V were needed for the W‐NiS_0.5_Se_0.5_/W‐NiS_0.5_Se_0.5_ configuration to achieve the current densities of 10 and 100 mA cm^−2^, respectively. Also, Wang et al.^[^ ^222^ ^]^ used a single‐step HT technique to prepare IT‐MoS_2_ with a flower‐like structure, displayed in Figure 18e,f. Ni, Co, and Fe transition metals were consecutively deposited onto IT‐MoS_2_. Ni‐1T‐MoS_2_ had improved bifunctional catalytic activity for both HER and OER in alkaline media with low η: 112 mV for HER and moderate η: 224 mV for OER, illustrated in Figure 18g,h. Ultimately, Ni‐IT‐MoS_2_/Ni‐IT‐MoS_2_ electrolytic cell achieved an overall water‐splitting with a cell voltage of only 1.54 V, recorded in Figure 18i. Similarly, Ahamd et al.^[^ ^223^ ^]^ developed NiS/MoS_2_ embedded multiwall carbon nanotubes (MWCNT/NiS/MoS_2_) catalysts; the TEM and HRTEM images are shown in Figure 18j,k. For HER and OER, MWCNT/NiS/MoS_2_ hybrid provided moderate η: 201 mV and low η: 193 mV for OER and HER, respectively, and exhibited stable performance up to 3000 cycles (Figure 18l–o).

A unique structure of nanosheets for V_0.3_‐NiS/NiS_2_ catalyst was prepared by Xu et al.^[^ ^224^ ^]^ This heterointerface's as‐generated dynamic and self‐equilibrium electron environment modifies an ideal adsorption harmony toward different H/O‐containing intermediates, improving the HER, OER, and overall water‐splitting ’s intrinsic activity and reaction kinetics. For HER and OER, V_0.3_‐NiS/NiS_2_ showed an ultra‐low η: 94 and moderate η: 220 mV. Similarly, two distinct nanostructures of two different, highly potent active electrocatalysts were hybridized and incorporated into a single heterostructure (P, Fe‐(VCo) Se_2_) on Ni‐Foam by Narayan et al.^[^ ^225^ ^]^ P, Fe‐(VCo)Se_2_ provided exceptional performance with ultra‐low η: 51 mV for HER and moderate η: 250 mV for OER activity. To achieve **J**_10_ for overall water‐splitting, P, Fe‐(VCo)Se_2_ needed a cell potential of 1.48 V. Moreover, to modify the electrical structure of Ni sites and activate their concealed catalytic potential, Xue et al.^[^ ^226^ ^]^ created heterostructures of nickel selenide (NiSe) and carbon nanotubes (CNTs). As anticipated, the electrocatalyst demonstrated improved activity for HER with moderate η:27 mV and low η_100_:145 mV. With a cell voltage of just 1.43 V at **J**_10_, NiSe@CNTs, as bifunctional catalysts, efficiently split water in a two‐electrode system.

Guo et al.^[^ ^227^ ^]^ using a sequential ionic layer adsorption reaction method (SILAR) and simple HT methodology, a high conductivity and porous structure of Ni foam substrate, intrinsic activity of sulfides, and interface among sulfides and Ni foam prepared CuInS_2_/SnS_2_/NF with a large specific surface area. As obtained heterostructure array demonstrated OER activity with moderate η:258, moderate η_20_: 286, and high η_100_: 382 mV in an alkaline environment, as well as high electrocatalytic efficacy toward HER with ultra‐low η: 78, low η_20_: 191, and high η_100_: 337 mV. Additionally, the overall water‐splitting performance of CuInS_2_/SnS_2_/NF electrode was investigated and found to be 1.58 V at **J**_10_. Like this, the coupled interface between Co_9_S_8_ and CoNi_2_S_4_ phases boosts the reaction kinetics of water‐splitting and enhances the adsorption of multiple intermediates to improve electrocatalytic performance. Chen et al.^[^ ^228^ ^]^ fabricated a 3D Co_9_S_8_@CoNi_2_S_4_ heterostructure that integrates 1D nanowires with high ion/electron transport, 2D nanosheets with an extensive specific surface area, and 3D NF with high electrical conductivity. The electrode shows a strong coupling between the CoNi_2_S_4_ and Co_9_S_8_ phases. In 1 m KOH solution, Co_9_S_8_@CoNi_2_S_4_/NF heterojunction catalyst exhibited an electrocatalytic activity, with low η: 170 mV for OER and ultra‐low η: 68 mV for HER. Furthermore, Zao et al.^[^ ^229^ ^]^ created Fe‐doped Ni_3_S_2_/MnS heterostructure on NF, 3D honeycomb structure with lots of voids, using ST process. Ni_3_S_2_/MnS effectively controls electronic structure and active sites through electron redistribution, resulting in an electron‐deficient state, advantageous for maximizing the adsorption energy of intermediates comprising oxygen. The obtained Fe‐doped Ni_3_S_2_/MnS, based on the benefits, showed remarkable OER catalytic activity with moderate η_50_: 259 mV as well as exceptional durability for 4000 cycles in 1 M KOH.

Zheng et al.^[^ ^230^ ^]^ using a multistep impregnation and electrodeposition process to design a new heterostructure catalyst, Co_9_S_8_/Cu_2_S on copper foam (Co_9_S_8_/Cu_2_S/CF). TEM image revealed a heterogeneous structure, comprised of Cu_2_S nanorods and Co_9_S_8_ nanosheets. OER and HER show low η: 195 mV and η: 165 mV, respectively, using Co_9_S_8_/Cu_2_S/CF catalyst. Moreover, the catalytic threshold of Co_9_S_8_/Cu_2_S to split water in a bipolar electrode is merely 1.67 V at **J**_10_. Concurrently, the embedding of Cu‐cation may promote the creation of high‐valent Ni/Co sites by improving the microstructure and raising the specific surface area of the electrocatalyst. Thus, using ST reaction, So et al.^[^ ^231^ ^]^ created a series of M(M = Fe, Cu, Zn, Mo)‐Ni_3_S_2_/Co_3_S_4_ materials. Cu‐Ni_3_S_2_/Co_3_S_4_ manifested significantly thinner nanosheets than Ni_3_S_2_/Co_3_S_4_. HER and OER performance were noticed by Cu‐Ni_3_S_2_/Co_3_S_4_, with an ultra‐low η: 79 mV and moderate η_250_: 250 mV, respectively. Additionally, Cu‐Ni_3_S_2_/Co_3_S_4_//Cu‐Ni_3_S_2_/Co_3_S_4_ only needed a cell voltage of 1.49 V to generate **J**_10_ for overall water‐splitting. Also, Jin et al.^[^ ^232^ ^]^ synthesized Ni–S, Fe–S, and Ni–Fe–S using an ion‐exchange technique. Specifically, Ni–Fe–S performed OER and HER, requiring only moderate η: 223 and low η: 115 mV, respectively. Furthermore, a two‐electrode setup was put up with Ni–Fe–S catalyst, where Ni–Fe–S|Ni–Fe–S demonstrated a considerable durability advantage over Pt/IrO_2_. The cell voltage needed to produce a **J**_10_was ≈1.57 V.

The catalytic activity of chalcogenide‐based electrocatalysts toward water‐splitting is described in Table 6 and Figure 17b. These materials exhibit favorable kinetics, as evidenced by their capacity to attain low Tafel slopes and overpotentials, which poses them well for real‐world HER and OER applications. Among these, transition metal dichalcogenides (TMDs) such as MoS_2_ and WS_2_ have been highly researched; nevertheless, their OER activity is limited unless enhanced by heterojunction formation or phase engineering. For instance, monolayer MoS_2_ exhibits poor OER activity unless co‐catalyzed with WS_2_, and even in that case, improvement in performance is limited.^[^ ^233^ ^]^ Semiconducting 2H‐phase TMDs are plagued by comparatively low electrical conductivity, whereas metallic 1T‐phase materials exhibit improved conductivity but are usually thermodynamically unstable. Methods like lithium intercalation in WS_2_ have been reported to enhance conductivity and lower Tafel slopes (e.g., ≈70 mV dec^−1^), but these are still behind benchmark noble‐metal catalysts.^[^ ^234^ ^]^ Moreover, most chalcogenides, particularly Ni‐Co selenides, are subject to surface change under oxidative OER conditions to form metal oxides or (oxy)hydroxides and function as precatalysts instead of the real active phases. Aside from intrinsic conductivity and structural instability, chalcogenides demand sophisticated defect tuning, elemental doping, intercalation, or heterostructure engineering to become significantly active, thus complicating synthesis and limiting scalability.^[^ ^235^ ^]^ Furthermore, more in‐depth atomic‐level insights into HER and OER mechanisms in both acidic and alkaline conditions are still required to optimize these materials. Although 2D chalcogenides remain theoretically and experimentally promising, practical applicability is thwarted by chemical instability and long‐term stability in extreme environments. In order to bypass these constraints, incorporation of nanocomposites or heterojunction‐based hybrids, integrating chalcogenides with other electrocatalysts like metal oxides, phosphides, hydroxides, or carbonaceous supports, is a compelling path forward. Such nanoscale interfaces can substantially promote charge separation, increase electron transfer, and induce synergistic effects, finally resulting in enhanced catalytic efficiency and operational stability.

**Table 6:** Chalcogenides‐based catalysts for water electrolysis.

Electrocatalyst	Synthesis	Morphology	Electrolyte	Hydrogen evolution		Oxygen evolution		Refs.
				η [mV]	Tafel Slope [mV dec^−1^]	η [mV]	Tafel Slope [mV dec^−1^]	
MoS_2_	HT Method	Nanosheets	H_2_SO_4_ (HER) KOH (OER)	248	84	300	220	[236]
FeCuS	HT Method	Nanorods	KOH	292	195.2	216 η_100_	88.1	[237]
CrNiSe_2_	HT Method	Nanosheets	KOH	89	98	27	110	[238]
FeS	Electrochemical strategy	Nanosheets	KOH	–	–	320	69	[239]
Fe_0.9_Ni_2.1_S_2_	Chemical etching process	Nanosheets	KOH	72	76	252	64	[240]
CuNiS	Chemical bath deposition	Nanospheres	KOH	99	63	337	43	[241]
FeCoNiS_x_	Annealing	Nanowires	KOH	97	88	260 η_50_	72	[242]
Ni_3_S_2_	HT Sulfurization	Nanoflakes	KOH	–	–	255.4	26.46	[243]
SnSe_2_	Colloidally synthesized	Nanoplates	H_2_SO_4_	289	79	–	–	[244]
FeS	Sulfurization etching	Nano fan‐like structure	KOH NaCl	63	95	–	–	[245]
Ni_3_S_2_	HT	Nanorods	KOH	–	–	263 η_100_	72	[246]
Cu_2_S	HT	Micro Flakes	KOH	179	68	260	110	[219]
NiCo_2_S_4_	HT	Caterpillar‐Like	KOH	216	138	290	139	[220]
NiFeS	ST	Nanospheres	KOH	115	108	223	41	[221]
CoMnNiS	Electrodeposition	Nanoflakes	KOH	226	101	371	48	[247]
CoMoS_4_	Ultrasound HT	Nanoflower	KOH	141	60.1	250	51.9	[248]
Co_0.9_Fe_0.1_Se	Electrodeposition	Porous Nanosheets	KOH	135	85.4	245	55.6	[249]
W‐NiS_0.5_Se_0.5_	Single atom doping	Nanorods Raped Nanosheets	KOH	39	51	171	41	[218]
Mo‐NiS_0.5_Se_0.5_	One‐step method	Nanorods	PBS	209 η_1000_	52	514 η_1000_	48	[250]
Mo‐NiFeSe	HT Method	Nanosheets	KOH	107 η_100_	73.82	325 η_100_	55.19	[251]
Co‐MoS_2_	HT Method	Nano Catalyst	H_2_SO_4_	58	72	–	–	[252]
Mo–MoS_2_	Carbonization	Nanosheets	KOH	117	64.3	390	72	[253]
Re‐ReS_2_	HT	Irregular Nanoparticles	KOH	42	36	–	–	[254]
N‐Fe‐Ni_3_S_2_	HT	Nanosheets	KOH	–	–	270 η_100_	58.3	[255]
Ni‐MoS_2_	HT	Flower‐like nanostructures	KOH	112	48.2	224	54.5	[222]
Mn‐SnS_2_	HT Sulfurization	Nanosheets	KOH	71	72	–	–	[256]
Co‐SnS_2_	HT	Flower‐like nanostructures	KOH	–	–	323	60.61	[257]
Fe‐V_x_Ni_3_S_2_	HT	Spherical nanostructures	KOH	–	–	253 η_100_	53.3	[258]
N‐CoSe_2_	HT	Nanosheets	KOH	10	109	237	49	[259]
P‐NiS_2_	Prussian‐Blue‐Analogue‐Sacrificed	Microspheres	KOH	73	86.51	255 η_20_	118.17	[260]
Fe‐Ni_3_S_2_	Chemical vapor transport method	Nano Particles	KOH	75	95	267	36	[261]
Au/Ni_3_S_2_	HT	Nanosheets	KOH	97	72	230	51	[262]
NiCo_2_S_4_/ReS_2_	HT Method	Nanosheets	KOH	85	78.29	–	–	[263]
MWCNT/NiS/MoS_2_	HT Method	Dense particle structure	H_2_SO_4_	193	41	201	58	[223]
V‐NiS/NiS_2_	Orbital occupancy	Nanorods	KOH	94	82	220	72	[224]
P,Fe‐(VCo) Se_2_	HT Method	Hybridized Nanosheet Nanorods	KOH	50	69	230	55	[225]
Fe‐Ni_3_S_2_/Ni_2_P	HT Method	Nanoflower linked nanosheet	KOH	172	87	112	67	[264]
ZIF67/CoNiSe	Ultrasound‐assisted self‐assembly and electrodeposition	Nanoflower	KOH	49	41.4	130	44.3	[265]
Mo_2_N/NiS	In‐Situ HT Method	Nanoparticles	KOH	245	130	298	181	[266]
NiS_2_/MoS_2_/CNTs	HT	Nanosheets	KOH	149	88	315	63	[267]
NiSe/CNTs	Electrodeposition	Nanofibers	KOH	27	46.2	145 η_100_	10.5	[226]
Co_9_S_8_/Co_9_S_8_/MoS_2_	Sulfurization, pyrolysis, and HT method	Polyhedral	KOH	173	71.5	340	82.7	[268]
CoTe_2_/CuTe	HT Method	Nanosphere	H_2_SO_4_ KOH PBS	68 106 125.8	59.94 67.6 69.53	–	–	[269]
FeS_2_/CoS_2_	Coprecipitation method and annealing	Nanosheet	KOH	78.2	44	302 η_100_	42	[270]
MoS_2_/CoS_2_	HT	Nanoflakes	KOH	241.5	169.8	350	64.3	[271]
Ag_2_Se/Ag_2_S/CoCH	HT	Nanoneedle	KOH	35	169	440 η_100_	–	[272]
CuInS_2_/SnS_2_	HT	Nanosheets	KOH	78	94	258	49.2	[227]
H‐NiSe_2_/MoSe_2_	HT	Nanosheets	H_2_SO_4_	103	43.5	‐	‐	[273]
CoS/SnS_2_	HT	Nanosheets	KOH	151	180	327	75.3	[274]
Co_0.5_Fe_0.5_P/rGO	Nanocasting	Mesoporous	KOH	258	53.8	250	42.4	[275]
Co_9_S_8_/CoNi_2_S_4_	HT	Nanowires/Nanosheets	KOH	68	56	170	42	[228]
N,P‐Co_9_S_8_/CoS_2_/ Co_1‐x_S	Pyrolysis	Nanosheets	KOH	–	–	285	70	[276]
SnTe/GO	CR	Nanosheets	KOH	–	–	226	53	[277]
Fe‐Ni_3_S_2_/MnS	ST	Nanosheets	KOH	–	–	259 η_50_	39	[229]
MoS_2_/Ni_3_S_2_	HT	Spherical Heterostructure	KOH	109	81	130	91	[278]
Fe‐F‐Ni_3_S_2_	HT	Nanoarrays	KOH	–	–	320 η_100_	37.1	[279]
Ni_3_S_2_/FeNi_2_S_4_	HT	Nanocluster On Nanosheets	KOH	50	117	201	36.2	[280]
O,P‐NiMoS	Ion Exchange	Nanosheets	H_2_SO_4_ KOH	39 30	37 36	–	–	[281]
Mn_70_Cu_29_Ni_1_S	Sulfuration	Nanoporous Network	KOH	136 η_50_	69.9	317 η_50_	104.2	[282]
CoS_2_/MoS_2_	Chemical vapor sulfurization	Nano Sheets	KOH	82	59	266	104	[283]
Ni_3_S_2_/FeS	HT Method	Nano Sheets	KOH	190 η_30_	56.3	295 η_30_	79.8	[284]
Co_9_S_8_/Cu_2_S	Chemical method	Nano Rods	KOH	165	80.2	195	78.8	[230]
Cu‐Ni_3_S_2_/Co_3_S_4_	ST	Nanosheets	KOH	79	50.4	160 η_50_	59.7	[231]
CoS/NiS	Electrodeposition	Nanosheets	KOH	–	–	145	73.79	[232]
N‐Ni_3_S_2_/CoS_2_	Electrochemical Deposition	Nanosheets	KOH	100	89	245	84	[285]
NiCo_2_Se_4_/NiCoS_4_	HT	Flowers	KOH	180	107.4	248	98.5	[286]

### 3.7 Nanocomposites/Heterojunctions

**Figure 19:**
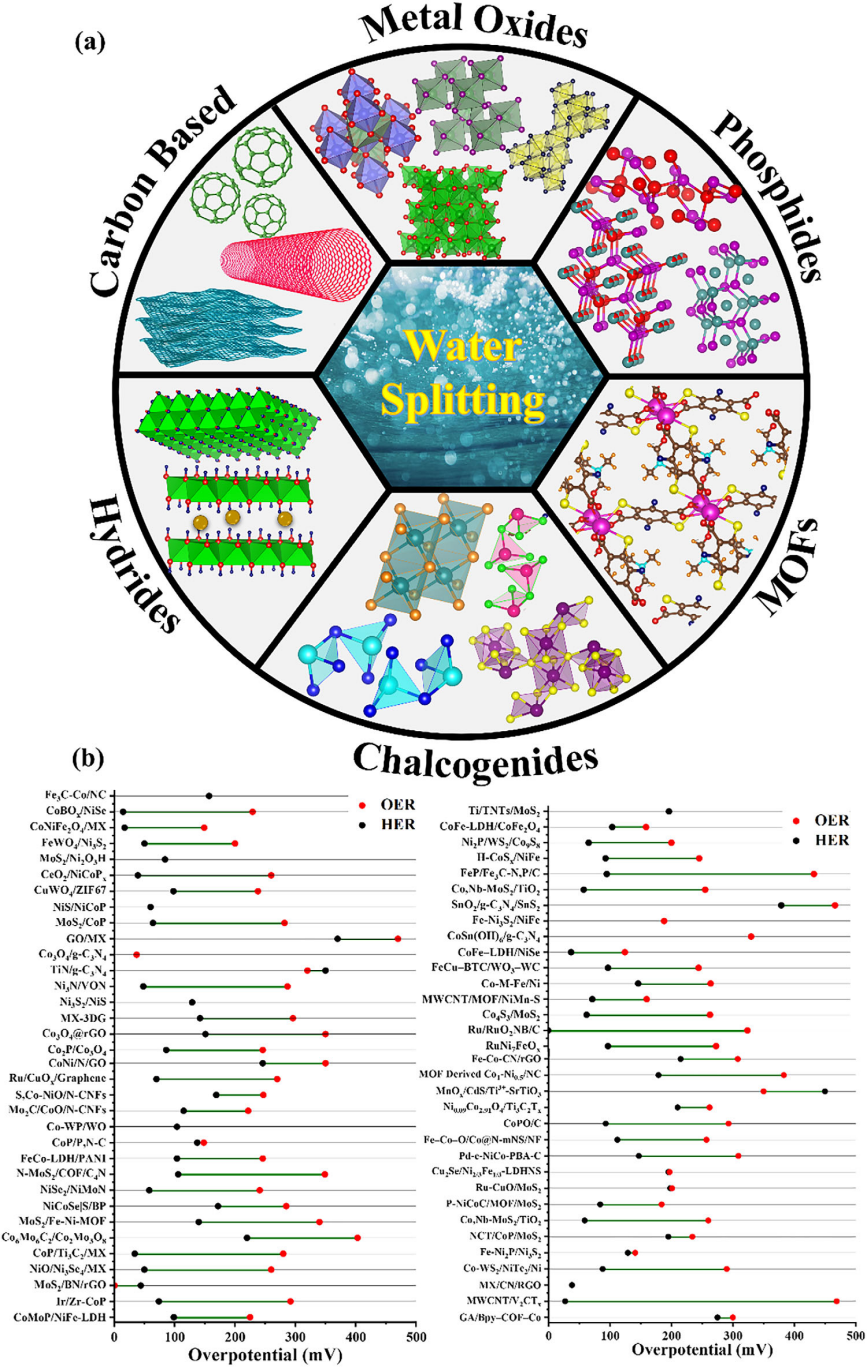
a) The formation of hybrid materials through the combination of hydrides/hydroxides, chalcogenides, MOFs, carbon‐based materials, phosphides, and metal oxides is illustrated, leveraging the dual properties of individual components to enhance hydrogen and oxygen evolution and b) Intercomparison of η for HER and OER efficacy of hybrid materials, based on **Table** **7**.

Carbon‐based materials, metal‐based electrocatalysts, phosphides, chalcogenides, MOFs (MOFs), and hydrides/hydroxides represent excellent classes of materials for electrocatalysis. To further enhance catalytic activity and address the inherent limitations of individual materials, the formation of heterojunctions and nanocomposites has emerged as a highly favored and viable strategy. The synergistic effects resulting from the combination of two or more materials can significantly improve electrocatalytic performance by increasing efficiency, reducing overpotential, and providing exceptional stability across various pH conditions and extended operational periods, as schematically provided in **Figure** **19**a.

A comprehensive discussion on the role and advantages of nanocomposites in electrocatalysis is provided below. Active sites exert influence in electrocatalytic reactions as they facilitate chemical transformations at the electrode‐electrolyte interface. These sites possess specific structural and chemical properties that lower activation barriers and enhance the rate of reaction. Maximizing active site availability and accessibility is essential for improving electrocatalytic efficiency. In this respect, Hu et al.^[^ ^287^ ^]^ developed an interface between P‐doped MoS_2_ and CoP on CC to improve water‐splitting efficacy. The catalyst exhibited ultra‐low η: 64 mV for HER and moderate η: 282 mV for OER in 1 m aqueous KOH with long‐term stability and two‐electrode cell voltages of 1.83 and 1.97 V at 500 and 1000 mA cm^−2^, respectively. Similarly, Wen et al.^[^ ^288^ ^]^ fabricated in situ hybrid nanowire‐nanosheet structure of CeO_2_ and NiCoP catalyst using the template of Ni‐Co foam to determine its efficiency toward overall water‐splitting. Undoubtedly, hybrid architectures of nanowires and nanosheets offer enough ion transport channels and adequate contact surfaces for electrochemical reactions. The composite thus displayed an ultra‐low η: ≈39 mV and moderate η: ≈260 mV for HER and OER, respectively in 1 m KOH. Following an extended time (i.e., 100 h), negligible changes in performance were reported, and the composite well retained its morphology. CeO_2_/NiCoP_x_/NCF required 1.49 V to attain **J**_10_, better than a noble metal catalyst (1.52 V), ascribed to reduced charge transfer resistance throughout the electrochemical reaction. In another investigation, Wang et al.^[^ ^289^ ^]^ carried out ST method followed by calcination to design carbon‐encapsulated FeWO_4_/Ni_3_S_2_ having flower‐like morphology. The composite required ultra‐low η: 50 mV for HER and low η: 200 mV for OER in 1 m KOH and was found stable when steadily operated for 100 h at 1000 mA cm^−2^ in 6 m KOH. The exceptional catalytic performance is credited to multiple factors. They include carbon encapsulation, which shields the catalyst from corrosion in an alkaline environment, the charge transport between Ni_3_S_2_ and FeWO_4_ at the interface to boost intrinsic activity, and flower‐like morphology, which provides improved distribution of active sites for reaction and improves stability by desorbing gas bubbles.

Electronic interactions are pivotal in enhancing electrocatalytic performance by modulating charge transfer processes and accelerating the reaction kinetics. Therefore, synergistic interactions in composite catalysts lead to amplifying catalytic activity beyond individual components. Understanding and controlling these interactions are essential for designing exceptional electrocatalysts with top‐tier efficiency. In this regard, Xiong et al.^[^ ^290^ ^]^ constructed a heterointerface of Ni_3_N and vanadium oxynitride (VON) porous nanosheets on carbon fibers (CF) to effectively trigger the electronic interactions among Ni and V‐atoms via HT method and nitridation. The composite catalyst exhibited ultra‐low η: 48 mV for HER and moderate η: 287 mV for OER in 1 m KOH. As‐prepared catalyst needed an overall voltage of 1.63 V to attain **J**_10_ for alkaline water‐splitting. Furthermore, Ni_3_N/VON/CF displayed an extraordinary stability for 40 h. In another study, Mu et al.^[^ ^291^ ^]^ employed a controllable synthesis to develop a novel interface between pure CoP and Co_3_(Si_2_O_5_)_2_(OH)_4_ nanosheets (CoP/CSNSs) to precisely design the electrocatalyst. The electrocatalyst demonstrated moderate η: 251 mV toward HER and high η: 309 mV for OER. While the cell required a potential of 1.50 V for overall water‐splitting, exceeding the Pt/C||RuO_2_ system. The outstanding performance of CoP/CSNSs in HER and OER is primarily associated with the optimized surface electronic structures resulting from phase modification of pure CoP and abundance of catalytically active sites facilitated by Co_3_(Si_2_O_5_)_2_(OH)_4_ nanosheet structures. The neighboring CoP and Co_3_(Si_2_O_5_)_2_(OH)_4_ components serve as active catalysts, surficial CoP improves the Volmer step of HER via dissociation of H‐OH bonds and Co_3_(Si_2_O_5_)_2_(OH)_4_ support enhanced OH‐ adsorption to boost the OER kinetics; collectively facilitating the respective reaction steps and synergistically enabling efficient water‐splitting. Similarly, Wang et al.^[^ ^292^ ^]^ obtained an optimized electronic structure having an elevated density of states near the Fermi level to trigger the adsorption and take advantage of the synergistic effects of NiSe_2_ and NiMoN. NiSe_2_ nanoparticles were found anchored on NiMoN nanorods. As‐prepared catalyst needed an ultra‐low η: 58 mV for HER and moderate η: 241 mV for OER in 1 m KOH.

Chen et al.^[^ ^293^ ^]^ devised an approach to assemble a multicomponent nanocomposite of functionalized MWCNTs, Cu‐based MOF(HK), and sulfurized NiMn LDH (NiMn‐S). MW/HK/NiMn‐S had a distinctive design with a lot of electroactive Cu/Ni/Mn sites and porous morphology. Nanocomposite catalyst drove ultra‐low η: 73 mV for HER and low η: 163 mV for OER, argument was supported by Tafel slopes of 75 and 57 mV dec^−1^, respectively. The superb electrocatalytic activity is linked to the design of the nanocomposite catalyst. The multicomponent composite with dual‐functionality in electrocatalysis for both HER and OER entails harnessing the electrical conductivity inherent in MW, benefits of enhanced surface area and facile mass transport by HK, and incorporating cost‐effective NiMn‐S to provide additional transition metal active centers. In another research, Huang et al.^[^ ^294^ ^]^ employed a facile sol‐gel method followed by supercritical dehydration to fabricate novel Ru‐NiFe‐oxyhydroxide anchored onto N‐carbon aerogel (RuNi_7_FeO_x_(OH)_y_ @NCA). The characteristic “pearl‐like” aerogel structures of carbon overlap the oxyhydroxide, facilitating improved electron transport via thin tunnels having a pore width of ≈7.0 nm, providing diffusion pathways for reaction intermediates. Consequently, the novel composite catalyst provided remarkable HER and OER activity with ultra‐low η: 99 mV and moderate η: 278 mV, respectively, in 1 m KOH. This improvement arises from the addition of Ru, which modifies the electronic structure of nearby Ni and Fe, providing adsorption strength to reaction intermediates and hence improved catalytic activity. For the same purpose, Gong et al.^[^ ^295^ ^]^ incorporated the electrospinning method to design Mo_2_C‐CoO encapsulated in N‐doped carbon nanofibers (Mo_2_C‐CoO@*N‐*CNFs). The compositions were authenticated using XPS measurements, revealing the co‐existence of Mo, Co, C, N, and O, as well as specific chemical bonds and functional groups such as Mo‐N, pyridinic‐N, pyrrolic‐N, and graphitic‐N, which contribute toward enhanced surface wettability, catalytic activity, and rapid electron transport. Mo_2_C‐CoO@*N‐*CNFs established excellent electrocatalytic activity as a bifunctional catalyst in an alkaline electrolyte, providing low η: 115 mV and moderate η: 222 mV for catalyzing HER and OER, respectively. The catalytic performance is attributed to porous N‐CNFs, which provide increased availability of active sites and Mo_2_C‐CoO hetero‐structure that enhances conductivity and stability. As‐synthesized composite electrocatalyst was superior to Pt/C‐NF //RuO_2_‐NF cell in a two‐electrode system with 1.56 V at **J**_10_.

**Figure 20:**
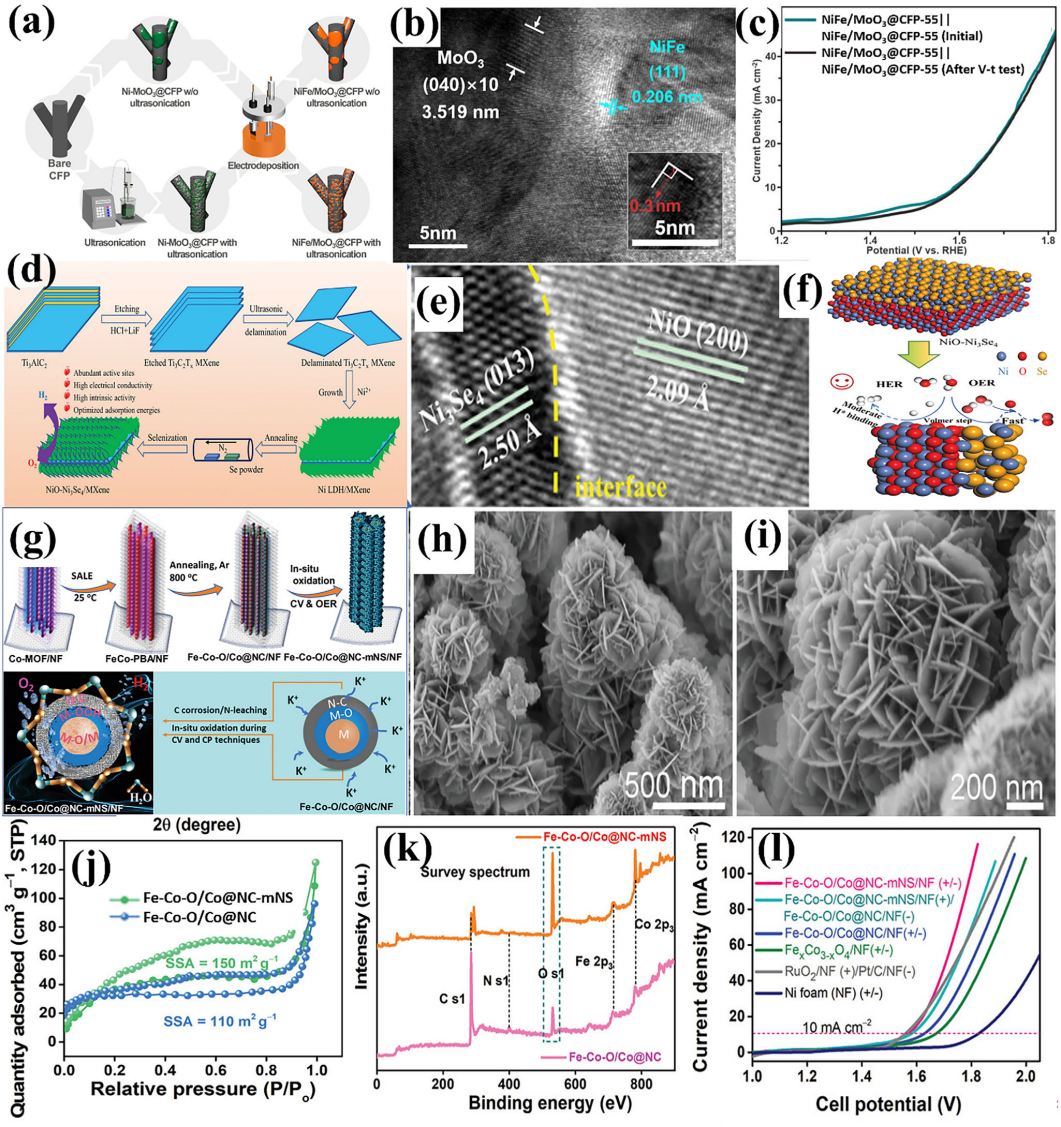
a) Schematic of NiFe/MoO_3_@CFP synthesis, b) Verification of NiFe/MoO_3_@CFP via HRTEM, c) Durability test of NiFe/MoO_3_@CFP, d) Schematic of NiO/Ni_3_Se_4_/MX synthesis, e) HRTEM image of NiO/Ni_3_Se_4_/MX, f) Mechanisms involved in HER and OER for NiO/Ni_3_Se_4_/MX, g) Schematic development of Fe‐Co‐O/Co@NC mNS/NC/NF, h,i) HRTEM images of Fe‐Co‐O/Co@NC mNS/NC/NF, j) N_2_ adsorption‐desorption isotherm of Fe‐Co‐O/Co@NC mNS/NC/NF, k) XPS survey spectra of Fe‐Co‐O/Co@NC mNS/NC/NF, l) LSV profile of Fe‐Co‐O/Co@NC mNS/NC/NF in two‐electrode arrangement. Reproduced from,^[^ ^296^, ^297^, ^298^ ^]^ with permission of Elsevier and Wiley.

The synthesis method significantly influences electrocatalytic efficacy by varying the configuration, surface morphology, crystallographic structure, and topographical properties of the catalyst. Through controlled synthesis methods such as ST or HT routes, controlled manipulation over particle size, shape, and crystallinity can be achieved, which affects the surface area and active sites exposure to get the desired catalytic activity. Cho et al.^[^ ^296^ ^]^ prepared NiFe/MoO_3_ on carbon fiber paper (CFP) through electrochemical deposition and ultrasonic synthetic technology, illustrated in **Figure** **20**a, and confirmed synthesis via HRTEM in Figure 20b. The technique entails the formation of bubbles, their expansion, and then collapse when ultrasonic waves are applied and generates defects and strain on the surface of solids. These defects favor the integration of H^+^ and evolution of H_2_, improving the overall water‐splitting. For OER and HER, NiFe/MoO_3_@CFP attained moderate η_20_: 251 mV and η_20_: 204 mV, respectively in 1 m KOH. Collectively, the water‐splitting process was determined to require a cell potential of 1.589 V to accomplish **J**_10_ in two‐electrode arrangements. The catalyst's stability before and after V‐I test is presented in Figure 20c. Similarly, Yan et al.^[^ ^297^ ^]^ carried out partial selenization of nickel LDH /MX precursor to synthesize the hybrid NiO/Ni_3_Se_4_/MX, as shown in Figure 20d. NiO/Ni_3_Se_4_ nanosheets were arranged over a highly conducting MX substrate, evident from the HRTEM image in Figure 20e. The composite catalyst showed an ultra‐low η: 50 mV for HER, moderate η: 260 mV for OER, and attained a cell voltage of merely 1.54 V, surpassing that of Pt/C||RuO_2_ (1.59 V). During water electrolysis, preferential adsorption of H* species onto Ni_3_Se_4_, which is enriched with negative charges, facilitates H_2_ formation, while NiO, with abundant positive charge, captures OH‐ and catalyzes OER; hence, resulting in superior bifunctional activity for overall water‐splitting, the mechanism is also summarized in Figure 20f.

Singh et al.^[^ ^298^ ^]^ reported the synthesis of *in‐situ* oxidized Fe–Co–O/Co metal@N‐doped carbon mesoporous nanosheets anchored on Ni foam (Fe‐Co‐O/Co@NC mNS/NC/NF) by carbonization of FeCo–PBA/NF in an inert environment, illustrated in Figure 20g. The resulting composite demonstrated a highly porous nanosheet structure in Figure 20h,i, with a specific surface area of 150 m^2^ g^−1^ in Figure 20j, ascribed to oxidized Co species and their topotactic alterations, which supports the diffusion of electrolyte and offers interaction with active sites at the electrocatalyst surface. Furthermore, XPS investigations in Figure 20k revealed in situ oxidation‐induced physiochemical modifications, including abundant oxygen vacancies, which significantly influence catalytic performance. Fe‐Co‐O/Co@NC mNS/NC/NF ultimately demanded moderate η: 257 mV and low η: 112 mV for OER and HER, respectively. For the overall water‐splitting process, the catalyst required 1.58 V at **J**_10_ in two electrode arrangements, evident from Figure 20l.

**Figure 21:**
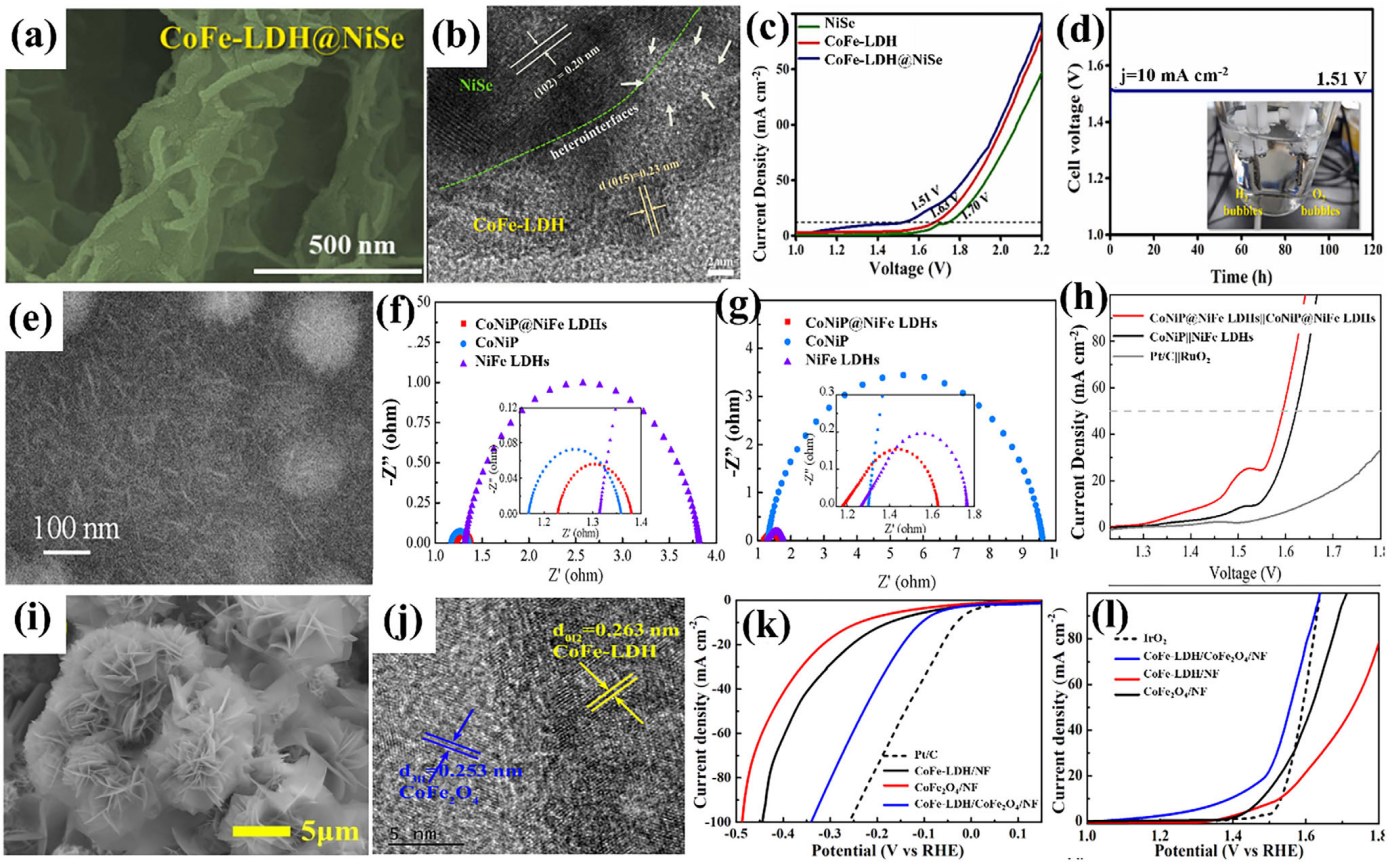
a,b) Morphological investigations of CoFe‐LDH@NiSe, c) Polarization curve for overall water splitting of CoFe‐LDH@NiSe, d) Durability test of CoFe‐LDH@NiSe for 120 h, e) Morphology visualization of CoNiP@NiFe‐LDHs, f) EIS spectra for HER of CoNiP@NiFe‐LDHs, g) EIS spectra for OER of CoNiP@NiFe‐LDHs, h) Polarization curve for overall water splitting using CoNiP@NiFe‐LDHs, i,j) Morphological analysis of CoFe‐LDH/CoFe_2_O_4_, k) HER polarization curve of CoFe‐LDH/CoFe_2_O_4_, l) OER polarization curve of CoFe‐LDH/CoFe_2_O_4_. Reproduced from,^[^ ^299^, ^300^, ^301^ ^]^ with permission of Elsevier.

Similarly, Jeghan et al.^[^ ^299^ ^]^ employed the interface linking of cobalt‐iron LDH and nickel selenide (CoFe‐LDH@NiSe) to investigate water electrolysis performance. CoFe‐LDH has many active sites but suffers from low conductivity, while NiSe possesses high conductivity but few active sites, the core‐shell architecture of the composite in **Figure** **21**a is thought to have high conductivity and increased reactive sites. The defects were also observed with HRTEM in CoFe‐LDH (Figure 21b), indicating extensive disordered lattice dislocations. This may enhance the accessible active sites and alter the properties at the interface, consequently leading to substantial augmentation in catalytic activity. The composite catalyst provided low η: 127 mV and ultra‐low η: 38 mV, with 37 and 33 mV dec^−1^ calculated values of Tafel slope for OER and HER, respectively, in an alkaline environment. CoFe‐LDH@NiSe exhibited a relatively lower voltage requirement, i.e., 1.51 V to achieve **J**_10_, notably surpassing both CoFe‐LDH (1.63 V) and NiSe (1.70 V) individually, shown in Figure 21c. The durability test was also conducted over 120 h, revealing consistent activity with an impressive retention rate of 98%, recorded in Figure 21d. In another report, Li et al.^[^ ^300^ ^]^ assembled CoNiP and NiFe‐LDH to utilize their interface interaction for HER and OER. Morphological analysis in Figure 21e displayed the growth of NiFe‐LDH on CoNiP nanosheets, while EIS in Figure 21f,g unveiled lower charge transfer resistance for CoNiP@NiFe‐LDHs than CoNiP and NiFe‐LDHs, indicating superior reaction kinetics. The composite electrocatalyst required ultra‐low η: 68 mV for HER in an alkaline environment, outperforming both CoNiP (80 mV) and NiFe LDHs nanosheets (283 mV) at the same **J**. While for OER, CoNiP@NiFe‐LDHs remarkably attained moderate η_50_: 255 mV. The overall water‐splitting was further tested using CoNiP@NiFe‐LDHs as anode and cathode, simultaneously, and achieved 100 mA cm^−2^ at 1.641 V, attached in Figure 21h. Similarly, Gou et al.^[^ ^301^ ^]^ constructed composites of CoFe_2_O_4_ and Co_3_O_4_ via HT method to effectively enhance the electrocatalytic activity by leveraging unique structural advantages, including interfacial bonding and electronic transport. The spherical morphology having vertically arranged nanosheets was visualized from the SEM and HRTEM images in Figure 21i,j, and such structures offer greater surface area and reactive sites for catalytic reactions. The resulting nanocomposite CoFe‐LDH/CoFe_2_O_4_/NF not only displayed HER and OER performance with low η: 106 mV and 162 mV (Figure 21k,l) but also provided a bias voltage of 1.55 V in a two‐electrode configuration in an alkaline medium.

In an investigation, Chen et al.^[^ ^302^ ^]^ fabricated novel Fe‐doped Ni_2_P/Ni_3_S_2_ from MOF, coated with carbon (NPZFNS@C/NF) having urchin‐like heterostructure and BET surface area of 15.3 m^2^ g^−1^ for water‐splitting. The interface between Ni_3_S_2_ and Ni_2_P efficiently controls the d‐band center and electronic structure, increases the number of active sites, and improves conductivity. Additionally, the carbon coating improves interfacial interactions and even makes electronic conduction faster. NPZFNS@C/NF manifested outstanding HER and OER activity with low η: 129 mV and η: 141 mV in an alkaline environment, respectively, favored by reaction kinetics with a Tafel slope of 66.8 mV dec^−1^ for HER and 72.8 mV dec^−1^ for OER. The electrocatalytic system requires 1.5 V to accomplish **J**_10_ when NPZFNS@C/NF is employed as both electrodes. NPZFNS@C/NF also maintained exceptional performance for 25 h, indicating outstanding stability. Local electronic structure reconfiguration and design of active and durable electrocatalysts through the introduction of appropriate transition metals impacts the electrochemical water dissociation performance. In this respect, Qiao et al.^[^ ^303^ ^]^ reported the fabrication of unique nitrogen‐doped carbon‐hosted polymetallic carbides (NC@Cu‐Co‐W‐C) from annealing of CuWO_4_@ZIF‐67 in an argon atmosphere. As‐prepared catalyst hosted metallic Cu, hexagonal WC, and Cu‐doped Co_3_W_3_C. The inclusion of Cu effectively prevents the formation of metallic Co during synthesis, while WC with transition metals Cu and Co promotes electron transfer within NC@Cu‐Co‐W‐C. This sophisticated structure facilitates the presence of abundant Co^3+^, low‐valence‐state Cu and W species, providing an increased abundance of active sites. The resulting catalyst exhibited moderate η: 238 mV for OER and ultra‐low η: 98 mV for HER. Furthermore, NC@Cu‐Co‐W‐C electrolytic cell required a cell potential of 1.64 V to achieve **J**_10_ in a two‐electrode configuration. In another research, Yan et al.^[^ ^304^ ^]^ utilized the precipitation method and in‐situ phosphorization to prepare novel CoP/Ti_3_C_2_ MX. Morphological examination revealed CoP nanoarrays were anchored vertically on the Ti_3_C_2_ MX substrate to pattern a hierarchical 3D porous structure with a reported large surface area and high conductivity. This greatly improves the accessibility to electrolyte, speeds up mass transfer, and allows for the speedy generation of effluents during electrocatalysis. Two electrode devices were constructed with CoP/Ti_3_C_2_ MX as positive and negative ends of the electrodes to investigate overall water‐splitting in alkaline conditions. The catalyst presented 1.578 V at **J**_10_, with a slight increase in potential, which was noticed after 60‐h operation.

**Figure 22:**
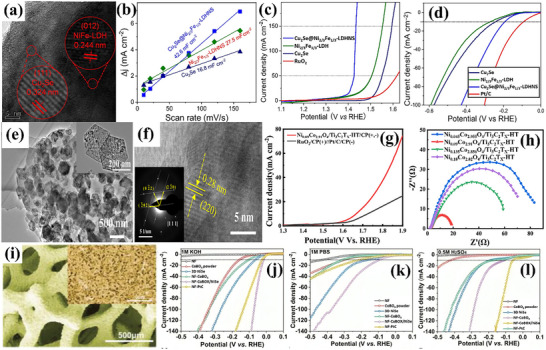
a) Morphology of Cu_2_Se@NiFe‐LDHNS, b) Capacitive current versus scan rate for Cu_2_Se@NiFe‐LDHNS, c) OER polarization curve for Cu_2_Se@NiFe‐LDHNS, d) HER polarization curve for Cu_2_Se@NiFe‐LDHNS, e,f) Morphology of Ni_0.09_Co_2.91_O_4_/Ti_3_C_2_Tx‐HT, g) Overall water splitting potential of Ni_0.09_Co_2.91_O_4_/Ti_3_C_2_Tx‐HT, h) EIS spectra of Ni_0.09_Co_2.91_O_4_/Ti_3_C_2_Tx‐HT, i) Morphology of CoBOx/NiSe, j) HER polarization curve for CoBOx/NiSe in an alkaline environment, k) HER polarization curve for CoBOx/NiSe in neutral environment, l) HER polarization curve for CoBOx/NiSe in an acidic environment. Reproduced from,^[^ ^305^, ^306^, ^307^ ^]^ with permission of Elsevier, Wiley, and American Chemical Society.

Optimizing non‐noble metal‐based bifunctional electrocatalysts is challenging; many attempts are readily available in the literature in this regard. Qi et al.^[^ ^305^ ^]^ designed ultrathin NiFe‐LDH nanosheets onto Cu_2_Se nanowires to prepare a core–shell nanostructure, authenticated via HRTEM in **Figure** **22**a. Cu_2_Se@NiFe‐LDHNS was characterized with a large electrochemical surface area, resulting in quick charge transfer via multiple pathways. The consequential capacitive currents as a function of scan rate are documented in Figure 22b. Such a characteristic enables the electrocatalyst to capture water molecules and its interaction with electrolytes to enhance the rate of reaction. Accordingly, the exceptional electrocatalytic activity is recorded for Cu_2_Se@NiFe‐LDHNS in alkaline medium (Figure 22c,d), requiring low η_50_: 197 mV for OER, low η: 195 for HER, and 1.67 V overall water‐splitting potential at **J**_10_ with robust stability for 40 h. Similarly, Xu et al.^[^ ^306^ ^]^ used HT method to synthesize Ni_x_Co_3–x_O_4_ hexagonal nanoplates assembled on Ti_3_C_2_T_x_ nanosheets. By varying Ni content, the facet structure of Ni_x_Co_3–x_O_4_ was controlled. Ni_0.09_Co_2.91_O_4_/Ti_3_C_2_T_x_‐HT exhibited a higher proportion of (111) rather than (112) facets, when compared to Ni_0.18_Co_2.82_O_4_/Ti_3_C_2_T_x_‐HT, credited to the surface reconstruction and led to form a highly active catalyst, the corresponding TEM image of Ni_0.09_Co_2.91_O_4_/Ti_3_C_2_T_x_‐HT is provided in Figure 22e,f. When utilizing Ni_0.09_Co_2.91_O_4_/Ti_3_C_2_T_x_‐HT in 1 m KOH, OER and HER with moderate η: 262 mV and η: 210 mV, respectively, were accomplished. In the context of overall water‐splitting, the same catalysts required ≈1.66 V to attain **J**_10_ (Figure 22g) and ensured 70 h of stable performance. This activity notably surpassed that of RuO_2_–Pt/C‐based catalyst and was attributed to the strong interaction and boundary‐interphase synergy in‐between Ti_3_C_2_T_x_ and Ni_x_Co_3–x_O_4_, suggesting a greater possibility for charge transfer, in accordance with EIS spectra (Figure 22h).

There are a few factors that significantly influence electrocatalytic performance by affecting the surface charge, adsorption energies, and thus the reaction kinetics of catalysis. pH, being one of them, alters the proton concentration in electrolyte and impacts proton‐coupled electron transfer reactions during electrochemical processes. That is why pH sensitivity is particularly of concern for water electrolysis, where maintaining stability and efficiency across a wide pH range is essential for practical implementation in diverse environments. Y. Liu et al.^[^ ^307^ ^]^ constructed an amorphous‐crystalline CoBO_x_/NiSe heterostructure, with the morphology shown in Figure 22i, aiming to enhance water electrolysis efficiency while maintaining stability across a range of pH conditions. CoBO_x_/NiSe surprisingly exhibited Pt‐beyond HER activity with ultra‐low η: 14.5 mV in alkaline environment, followed by ultra‐low η: 93.4 mV in neutral and low η: 107 mV in acidic conditions, results are attached in Figure 22j–l. The reaction kinetics were supported by Tafel slopes of 25.5, 131.6, and 153.4 mV dec^−1^ in acidic, alkaline, and neutral conditions, respectively. The outcomes surpassed those achieved by individual constituents of the composite catalyst. CoBO_x_/NiSe nanostructure also exhibited a very small Tafel slope of 11.7 and 166.6 mV dec^−1^ in alkaline and neutral environments, accompanied by moderate η: 229.1 mV and high η: 473.2 mV, respectively, and hence improved OER kinetics in comparison to individual CoBO_x_ and NiSe.

The catalytic performance of hybrid catalysts toward water electrolysis is presented in Table 7 and summarized in Figure 19b.

**Table 7:** Hybrid catalysts for water electrolysis.

Electrocatalyst	Synthesis	Morphology	Electrolyte	Hydrogen evolution		Oxygen evolution		Refs.
				η [mV]	Tafel Slope [mV dec^−1^]	η [mV]	Tafel slope [mV dec^−1^]	
SnO_2/_MoS_2_	HT Method	Nanoforest	KOH	127	73.2	290 η_50_	42	[308]
Ni_2_P/MoO_2_	Phosphorization	Nanorods	KOH	34	45.8	–	–	[309]
Ni_3_N/CeO_2_	HT	Catalyst Morphology	KOH	30	42.79	31 η_50_	64.44	[310]
Co_2_P/Co_3_O_4_	Heat treatment	3d Network Porous Frame Structure	KOH	86	49.7	246	69.5	[311]
Co_3_O_4_@rGO Co_3_S_4_@rGO	HT	Nanoboxes Nanosheets	H_2_SO_4_	151	153 59	350	121 65	[312]
MX‐3DG	HT	Nanonetwork	KOH	142	142.81	296	58.17	[313]
NiF/MoO_3_	Electrochemical decomposition method	Ultrasonication Process	KOH	204 η_20_	–	251 η_20_	78	[296]
Ni_3_S_2_/NiS	HT	Nanowire	KOH	129	75.5	–	–	[314]
Ni_3_N/VON	HT Method	Nanosheets	KOH	48	45	287	120.9	[290]
TiN/g‐C_3_N_4_	Ultrasonication process	Nanoflakes	KOH (OER) H_2_SO_4_ (HER)	350	142	320	181	[315]
Fe_2_O_3_/FeS	HT	Needle‐like particles	KOH	–	–	370 η_40_	90	[316]
Co_3_O_4_/g‐C_3_N_4_	Calcination	Nanosheets	KOH	–	–	37	123	[317]
Co_9_S_8_/Co_3_O_4_	HT	Nanoflakes	KOH	130	123	331 η_100_	65.5	[318]
GO/MX	Annealing	Nanoparticles	H_2_SO_4_ (HER) KOH (OER)	370	110	470	70	[319]
MoS_2_/CoP	HT	Needle‐like nanoarray	KOH	64	59.7	282	57.4	[287]
MOF/V_2_O_5_	HT	Nanorods	KOH	–	–	300 η_50_	50.3	[320]
NiS/NiCoP	HT Method	Smooth Uniform Cubes	H_2_SO_4_, KOH PBS	60 71 95	40 42 52	–	–	[321]
CuWO_4_/ZIF‐67	Pyrolysis	Core‐shell polyhedral structure	KOH	98	50	238	59	[303]
CeO_2_/NiCoP_x_	HT	Nanowire‐nanosheets	KOH	39	67	260	72	[288]
MoS_2_/Ni_2_O_3_H	HT Method	Nanosheet	KOH	84	82.3	–	–	[322]
FeWO_4_/Ni_3_S_2_	ST	Flower‐like nanosheets	KOH	50	54.1	200	39.4	[289]
C_3_N_4_/Black TiO_2_	HT	Nanosheets	KCl	990	38.96	1380	102.2	[323]
CoNiFe_2_O_4_/MX	Lewis molten salt approach	MX Sheets	KOH, H_2_SO_4_ Mg SO_4_	17	33	149	45	[291]
NiCo_2_S_4_/NiFeP	HT	Flower‐like nanosheets	KOH	205 η_100_	99	293 η_100_	110	[324]
CoBOx/NiSe	Solution process and in‐situ growth	Nanosheet	KOH, H_2_SO_4_ PBS	14.5	25.5	229.1	11.7	[307]
HfNiSe_2_/rGO	HT	Nanosheets	KOH	162	49	320 η_20_	66	[325]
Fe_2_P/Co_2_N	Electrodeposition	Nanosheets	KOH	131 η_500_	131	283 η_500_	38.6	[326]
Fe_3_C‐Co/NC	Template‐removal method	1D Carbon Nanotubes and 2D Carbon Nanosheets	KOH	157	108.8	–	–	[327]
CoP/Ni_3_FeN	HT Method	Nanosheets	KOH	88 η_100_	55	264 η_100_	62	[328]
CoMoP/NiFe‐LDH	HT ‐phosphating‐electrodeposition method	Nanoplates	KOH	98.8	93.9	225	55	[329]
Ir/Zr‐CoP	HT	Nanoneedle	KOH	74	77.1	292	70.4	[330]
Ni^2+^‐LaNiO_3_/CdS	Hybrid HT ‐reductive‐chemical method	Core‐shell structure	–	–	–	–	–	[331]
MoS_2_/BN/rGO	HT Method	Flower, Elongated Disk, Layered Structure	H_2_SO_4_ (HER) KOH (OER)	44	115	0.6	99	[332]
NiO/Ni_3_Se_4_/MX	Exfoliation	Nanosheet	KOH	50	42.9	260	39.6	[297]
MX/MoS_2/_FeVO	HT	Nanolayer	KOH	17.4	35.8	300 η_50_	49.6	[333]
CoP/Ti_3_C_2_/MX	In situ growth,	Nanosheets	H_2_SO_4_ KOH PBS	34 102 124	57.6 68.7 96.8	280	95.4	[304]
Co_6_Mo_6_C_2_/Co_2_Mo_3_O_8_	HT	Nanoparticles	KOH	220	104.7	403	89.7	[334]
MoS_2_/Fe‐Ni‐MOF	HT	Nanoflower	KOH	140	158	340	159.8	[335]
NiCoSe|S/BP	Exfoliation	Nanosheets	KOH	172	128	285	116	[336]
NiSe_2_/NiMoN	HT	Nanorods	KOH	58	68.7	241	95.2	[292]
Ag‐PMAc‐g‐CNT	Polymerization	Carbon nanotubes	KOH	–	62.74	–	22.62	[337]
N‐MoS_2_/COF/C_4_N	HT	Nanosheet And Nano Petal	H_2_SO_4_ for HER, KOH for OER	106	–‐	349	64	[338]
FeCo‐LDH/PANI	Electrodeposition	Irregular Structure	KOH	104	115	246	45	[339]
CoP/P,N‐C	Controllable Approach	Nanoneedle	KOH	137.6	59.89	148.4	56.40	[340]
Co‐WP/WO	Thermal evaporation	Nanowires	KOH	104	79	–	–	[341]
Mo_2_C/CoO/N‐CNFs	Electrospinning and calcination	Carbon Nanofibers	KOH	115	76	222	54	[295]
S,Co‐NiO/N‐CNFs	Electrospinning technique	Nanofibers	KOH	169	86.44	247	69.44	[342]
Ru/CuO_x_/Graphene	Carbonization	Carbon Nanosheets	H_2_SO_4_ (HER) KOH (OER)	70	64	270	67	[343]
CoNi/N/GO	Hummers method	Nanosheets	KOH	246	123	350	59	[344]
RuNi_7_FeOx (OH)/NCA	Sol‐gel method	Nano Pearls	KOH	99	61.1	278	102.7	[294]
Ru/RuO_2_NB/C	Thermal treatment	Nano‐Boomerangs	H_2_SO_4_	0	69.2	330	69	[345]
Co_4_S_3_/Mo_2_ C‐N SC	HT Method	Nanorods	KOH	63.6	92.1	268	132.4	[346]
MWCNT/MOF/NiMn‐S	HT Method	Nanosheets	KOH	73	75	163	57	[293]
Co‐M‐Fe/Ni	ST treatment and in‐situ growth	Nanorods	KOH	149	89	269	50	[347]
FeCu–BTC/WO_3_–WC	Hydro/ST and annealing processes	Nanodiamonds	KOH	99	29	249	22.98	[348]
Fe(OH)_3_/Ni_3_S_2_/NF‐D1–30	HT and hydrolysis	Nano Particles	KOH	–	–	242 η_50_	45	[349]
CoFe–LDH/NiSe	HT and electrodeposition	Nanosheets	KOH	38	33	127	37	[299]
FeS_2_‐MoS_2_/CoS_2_‐MOF	HT	Microporous Nanoarray	KOH	92	70.4	211 η_20_	64.5	[350]
CoSn(OH)_6_/g‐C_3_N_4_	Co‐Precipitation	Nanoparticles	KOH	–	–	336	50	[351]
Fe‐Ni_3_S_2_/NiFe LDH	HT ‐Electrodeposition	Nanosheets	KOH	–	–	192	43.1	[352]
SnO_2_/g‐C_3_N_4_/SnS_2_	Stober method	Hollow Nanospheres	KOH	386	291	475	253	[353]
Co,Nb‐MoS_2_/TiO_2_	HT	Hollow Microspheres	KOH	58.8	40	260	65	[354]
CoFe‐LDH/NiCo_2_O_4_	HT	Flower‐like nanosheets	KOH	44	39.8	370 η_50_	56.4	[355]
CoNiP/NiFe LDHs	Electrodeposition	Nanoplates	KOH	68	32	255 η_50_	78.8	[300]
FeP/Fe_3_C‐N,P/C	Solid phase polymerization	Nanoflakes	KOH	97	89.3	440	108.6	[356]
NiMoP/NiFe‐LDH	ST	Nanowires	KOH	–	–	299 η_150_	23.3	[357]
H‐CoS_x_/NiFe LDH	Wet chemical	Nanoarray	KOH	95	90	250	49	[358]
Ni_2_P/ WS_2_/Co_9_S_8_	Pyrolysis	Nanosheets	KOH	67	85.4	204	54.3	[359]
Pt/TiO_2_/Ni(OH)_2_	HT	Nanosheets	KOH	107 η_500_	38.9	434 η_1000_	53.2	[360]
CoFe‐LDH/CoFe_2_O_4_	HT	Flower sphere	KOH	106	93.1	162	85.6	[301]
Ti/TNTs/MoS_2_	HT	Nanosheets	H_2_SO_4_	200	113	–	–	[361]
GA/Bpy–COF–Co	Polymerization	Nanosheets	KOH	275	139	300	54	[362]
MWCNT/V_2_CT_x_	Wet chemical etching	Nanosheets	KOH	27	44	469	77	[363]
MX/CN/RGO	ST	Nanosheets	H_2_SO_4_	38	76	–	–	[364]
Co‐WS_2_/NiTe_2_/Ni	HT	Nanosheets	KOH	88	68	290	98	[365]
Fe‐Ni_2_P/Ni_3_S_2_	HT	Nano rods	KOH	129	66.8	141	72.8	[302]
NCT/CoP/MoS_2_	HT	Core shell	KOH	195	74	234	74	[366]
Co,Nb‐MoS_2_/TiO_2_	HT	Hollow structure	KOH	58.8	40.2	260	81.2	[354]
P‐NiCoC/MOF/MoS_2_	HT	Nanoflakes	KOH	84	96	184	63	[367]
Ru‐CuO/MoS_2_	HT	Flower Like	KOH	198	113	201	229	[368]
Cu_2_Se/Ni_2/3_Fe_1/3_‐LDHNS	Electrodeposition	Coreshell	KOH	195	69	197	42.1	[305]
Pd‐e‐NiCo‐PBA‐C	Cation exchange method	Nanosheets	KOH	147	67	309	67	[369]
Fe–Co–O/Co@N‐mNS/NF	Thermal decomposition	Micro pillar like	KOH	112	96	257	41.56	[298]
CoPO/C	Electrodeposition	Microspheres	KOH	93	103.3	293	111.4	[370]
Ni_0.09_Co_2.91_O_4_/Ti_3_C_2_T_x_	HT	Nanosheets	KOH	210	106	262	92	[306]
MnO_x_/CdS/Ti^3+^‐SrTiO_3_	Spray‐coating method	Nano Beads	KOH	450	85	350	54	[371]
MOF derived Co‐1–Ni‐0.5/NC	HT	Nanoparticles	KOH	179	110.9	383	113.2	[372]
Fe‐Co‐CN/rGO	Annealing	Lamellar	KOH	215	54	308	38	[373]

#### 3.7.1 Comparative Outlook

**Figure 23:**
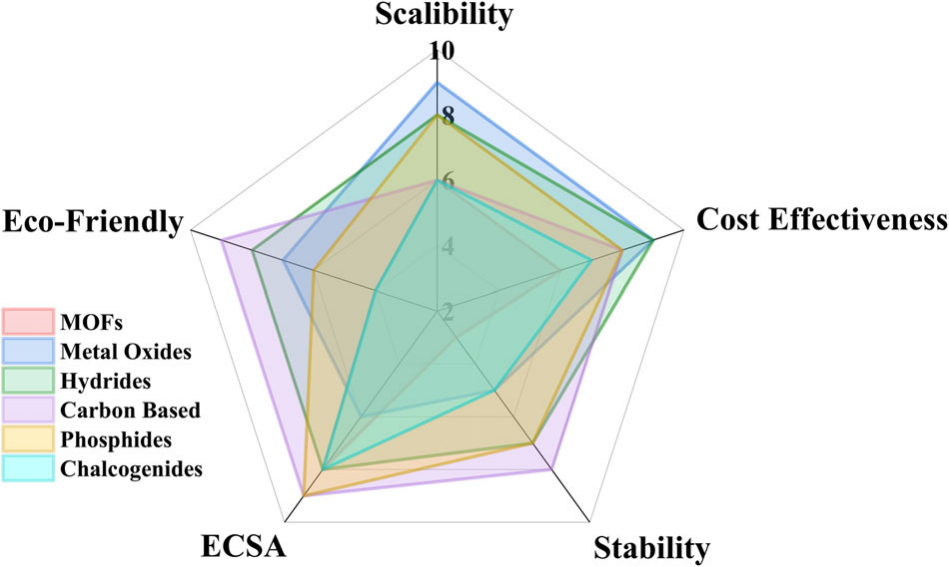
Comparative assessment of key electrocatalyst families for HER and OER based on five commercialization‐relevant criteria. Each parameter is rated on a scale of 1 to 10, where 1 indicates poor and 10 indicates excellent performance.

The potential for commercialization and practical implementation of these catalyst families was also thoroughly examined and summarized in **Figure** **23** based on key performance criteria including scalability, cost, structural stability, ECSA, and environmental impact.

Scalability remains one of the key parameters in translating lab‐scale electrocatalysts into real‐world hydrogen production technologies. Among all catalyst families, metal oxides and hydroxide/hydride‐based materials exhibit the most mature and scalable synthesis approaches, often synthesized through low‐temperature solution‐based methods that are readily adaptable to industrial‐level or continuous processing. Hydrides and hydroxides can be deposited through electrodeposition or co‐precipitation on conductive substrates, offering easy integration into an electrolyzer. In contrast, MOFs face notable scalability challenges due to their reliance on slow and solvent‐intensive synthesis. Carbon‐based materials such as graphene and metal carbides also struggle with scalable synthesis, particularly due to high‐temperature processes or exfoliation steps involved, although some advances in chemical vapor deposition (CVD) techniques are promising. Metal phosphides are relatively easy to scale, benefiting from simple phosphidation routes using abundant precursors. Chalcogenides vary in scalability: sulfides are easy to synthesize via hydrothermal or sulfidation routes, whereas selenides and tellurides face challenges due to precursor scarcity and handling requirements.

Cost‐effectiveness is closely tied to elemental abundance and the synthesis complexity of each material. Hydrides, hydroxides, and mixed‐metal oxides lead in this regard, as they rely on inexpensive, earth‐abundant elements and are typically synthesized through energy‐efficient processes. Metal phosphides also maintain low production costs and utilize widely available metals, though their synthesis sometimes involves gaseous or reactive phosphorus sources that require controlled environments. Carbon‐based catalysts (biomass‐derived carbon) are economically favourable; however, extensive post‐synthesis doping or carbide formation can increase overall cost. MOFs are constructed from cheap metals and ligands; however, their multistep, solvent‐heavy production adds to their overall cost. Chalcogenides rank lower in cost‐effectiveness when involving Se or Te due to elemental scarcity and higher precursor costs, though sulfides remain a low‐cost option among this class.

From an environmental perspective, hydroxide and hydride catalysts are among the most eco‐friendly materials, as their synthesis generates minimal waste and involves non‐toxic precursors. Carbon‐based materials may introduce moderate ecological concerns. Mixed‐metal oxides and phosphides fall in the middle range: while their metal precursors are generally non‐toxic and abundant, the presence of metals warrants‐controlled handling due to possible bioaccumulation. MOFs present environmental concerns due to their reliance on organic solvents and potential leaching of metal ions during degradation. Chalcogenides are most environmentally sensitive: while sulfides are relatively benign, selenides and tellurides pose ecological and health risks, especially due to Se/Te toxicity and poor recyclability.

ECSA is critical for maximizing catalytic efficiency by exposing a greater number of active sites. In this regard, MOFs, owing to their porous, crystalline frameworks, offer intrinsically high surface areas and tunable pore structures. Carbon‐based materials (graphene and carbon nanotubes) also demonstrate exceptional ECSA due to their architectures. Hydrides and hydroxides, particularly in LDH forms, provide high surface exposure through thin, nanosheet morphologies. Metal phosphides and sulfides exhibit favorable ECSA when nanostructured but tend to aggregate if not properly stabilized. Transition‐metal oxides and perovskites often show lower intrinsic surface areas unless nanostructured or composited with high surface area supports, making ECSA enhancement a key challenge in these materials.

Stability across varying pH ranges and prolonged operational periods is essential for commercial electrolyzer deployment. Metal oxides and phosphides show strong long‐term durability, especially in alkaline and neutral conditions, with phosphides exhibiting gradual surface transformation into active oxyhydroxides that preserve functionality. Hydroxides also demonstrate excellent alkaline stability, aided by their ability to self‐reconstruct under cycling conditions, although their performance in acidic media is less robust. Carbides are chemically stable and tolerate harsh electrochemical environments, while doped carbon materials are stable under HER but may degrade during prolonged OER due to oxidative corrosion. MOFs suffer from poor structural integrity in aqueous or high‐potential environments, where they often collapse or leach metal ions. Chalcogenides, particularly sulfides and selenides, are conditionally stable, showing good performance in HER but tending to oxidize or dissolve under sustained OER, limiting their standalone utility for bifunctional applications.

In light of the limitations faced by individual material classes, hybrid and composite catalysts have emerged as a strategic solution for optimizing electrocatalytic performance. By integrating complementary functionalities, such as combining high ECSA carbon materials with redox‐active metal oxides, hybrid catalysts can deliver synergistic enhancements in activity, stability, and conductivity. These materials can be rationally designed to address specific performance gaps: for example, coupling MOFs with conductive substrates can mitigate their poor conductivity, and stabilizing chalcogenides with oxide or carbon shells can suppress leaching and improve durability. Additionally, interface engineering, defect modulation, and heterostructure formation allow for tuning of electronic and surface properties to suit various operational conditions (e.g., acidic and alkaline). Thus, the development of tailored hybrid catalysts represents a promising direction for next‐generation water‐splitting technologies, enabling customizable and scalable solutions for clean hydrogen production.

#### 3.7.2 Conclusions and Perspectives

**Figure 24:**
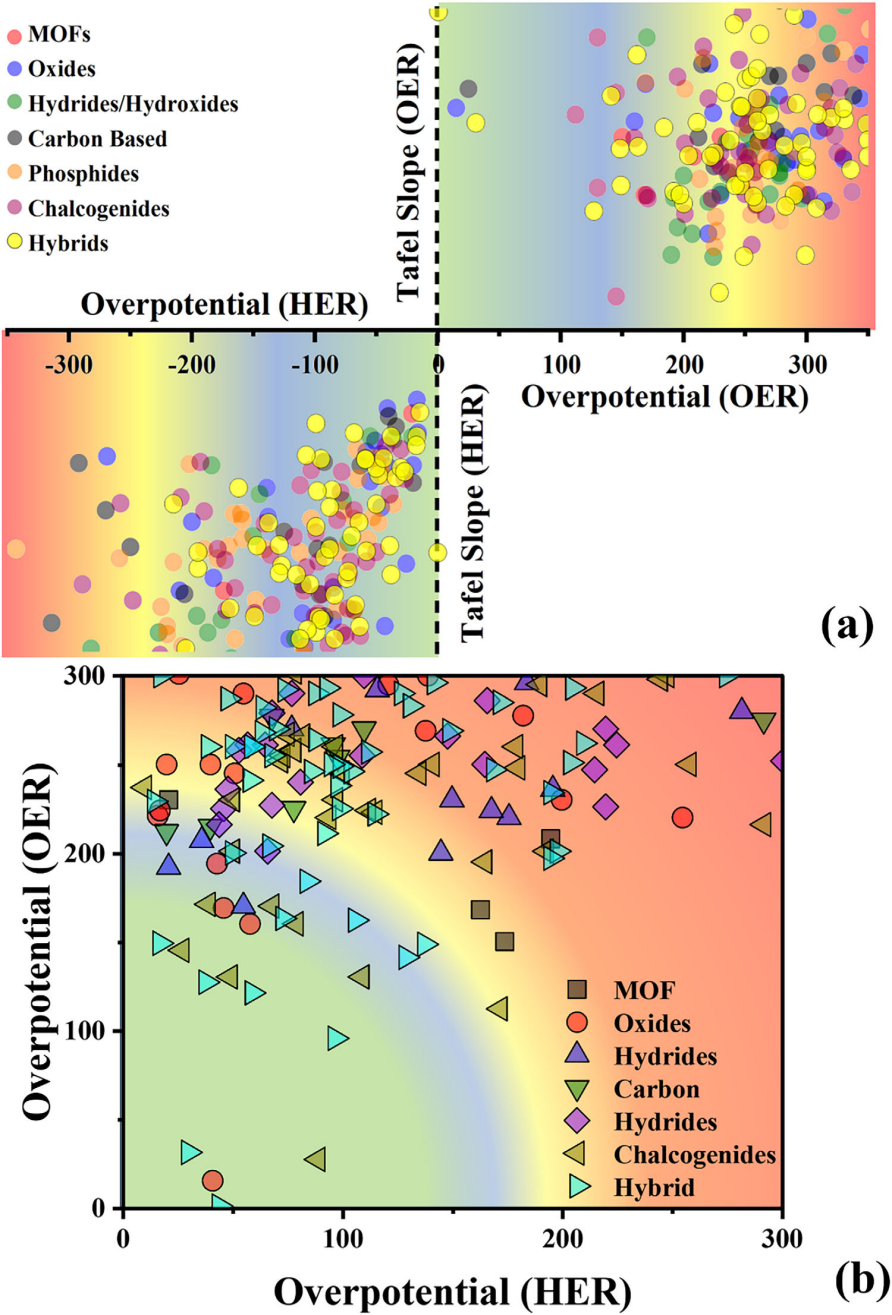
a) Inter‐comparison of HER and OER performance across diverse electrocatalyst families, including MOFs, metal oxides, hydrides/hydroxides, carbon‐based materials, phosphides, chalcogenides, and their hybrids–based on overpotential and Tafel slope values, b) Inter‐comparison of various electrocatalyst families based on HER versus OER overpotentials, highlighting the efficiency of different materials in catalyzing both reactions.

In conclusion, this review provides a comprehensive explanation of various parameters influencing bifunctional electrocatalysts for HER and OER in water electrocatalysis applications. A focal point is the in‐depth discussion of various electrocatalyst families, including metal‐organic frameworks, metal oxides, hydrides/hydroxides, carbon‐based catalysts, phosphides, chalcogenides, and their hybrids, the merits and limitations are summarized in **Table** **8**. It assessed their efficacy for enhanced HER and OER, noting their activity in different electrolytes, overpotentials, current densities, and Tafel slopes, with inter‐comparisons of catalytic performance for both reactions. The stability of materials under varying potentials was discussed, along with overall water‐splitting potentials at different current densities. Comparative data on overpotential and Tafel slopes for various catalyst families are presented in accompanying **Figure** **24**a,b, providing a clear overview of each material's effectiveness toward HER and OER.

**Table 8:** Comparison of different materials for electrocatalytic water splitting.

Class of materials	Merits	Limitations
Metal–organic frameworks (MOFs)	High surface area and tunable porosity Structurally versatile (chains, layers, clusters) Nanoporous structure supports efficient catalytic interaction	Very low electrical conductivity (≈10^−8^ S/cm) Structurally unstable in acidic or alkaline electrolytes Microporosity limits electrolyte and reactant diffusion Often act as precatalysts rather than stable active species
Metal‐based electrocatalysts (metal oxides, perovskites, mixed oxides)	Abundant, low‐cost, and versatile in composition Can be synthesized in diverse morphologies (0D–3D) Activity enhanced via doping, nanostructuring, and substrate tuning	Poor intrinsic conductivity Prone to degradation, surface dissolution, and elemental leaching Often unsuitable for PEM electrolyzers due to acidic instability
Hydrides and hydroxides	LDHs allow tuning via doping and interlayer spacing Potentially better performance under alkaline conditions Can be coupled with carbon supports for synergistic enhancement	Poor electrical conductivity requires conductive support HER kinetics can be sluggish, needing high overpotentials Morphological limitations reduce active site exposure Structural transformations reduce long‐term stability
Carbon‐based materials	Excellent electrical and thermal conductivity Chemically stable and mechanically robust Effective as support matrices for hybrid electrocatalysts Tunable structures from 0D to 3D with large surface areas	Prone to oxidation under high‐potential OER conditions Graphene restacking and CNT aggregation limit active site accessibility Complex and costly synthesis/functionalization Limited intrinsic catalytic activity; mostly supportive in composites
Phosphides	High conductivity Act as proton acceptors for enhanced OER Exhibit dual catalytic function in water splitting	Easily oxidized under ambient or operational conditions Phosphorus leaching in alkaline HER Transform into oxide phases under OER Toxic and hazardous synthesis methods Catalyst detachment at high current densities
Chalcogenides	Layered 2D structures with numerous active sites Facilitate ion diffusion and charge transfer Employed across a wide pH range with low overpotentials Tunable via doping/intercalation	Low OER performance unless engineered via heterostructures Surface oxidation during OER converts to oxides (precatalyst behavior) Complex synthesis limits scalability
Heterojunctions and nanocomposites	Combine strengths of multiple materials Enable synergistic improvement in charge separation and activity Allow tuning of interface properties Enhance stability across pH ranges and under prolonged operation	Synthesis can be technically challenging Interfacial compatibility and long‐term stability must be carefully optimized. Scalability of hybrid systems remains a bottleneck.

MOFs, metal oxides, hydrides/hydroxides, carbon‐based materials, phosphides, and chalcogenides all have unique electrocatalytic characteristics. For HER, materials like MOFs, metal oxides, carbon‐based catalysts, phosphides, and chalcogenides tend to have low to ultra‐low overpotentials and thus are good prospects. Conversely, high‐performing OER activity, with relatively lower overpotentials and moderate Tafel slope values, is predominantly found in MOFs, hydrides/hydroxides, and chalcogenides. Carbon materials and phosphides exhibit greater overpotentials for OER but are aided by good Tafel slopes, which favor rapid kinetics. Hybrids of the above material families offer enhanced electrocatalytic performance, exhibiting ultra‐low overpotentials for HER and moderate overpotentials for OER, along with low Tafel slopes. Using hybrid materials, such as nanocomposites or heterojunctions, improves both catalytic efficiency and stability, establishing them as suitable candidates for commercial‐grade electrocatalysts.

The analysis presented in Figure 24a,b highlights that hybrid materials consistently demonstrate the lowest overpotentials and superior kinetics compared to other electrocatalysts. By leveraging the complementary properties of different material families, hybrid systems enable ultra‐low overpotential electrocatalysis with low slopes, significantly improving reaction kinetics. However, for practical and commercial implementation, several critical factors must be addressed.

A detailed atomic‐level understanding of the catalytic process is necessary to ensure optimization of performance. Such as *in‐situ* characterization techniques as X‐ray absorption spectroscopy, environmental pressure XPS, and Raman spectroscopy are invaluable for understanding the dynamic behaviour of electrocatalysts under operational conditions. Those techniques also orient the refinement of dynamic and accurate theoretical models based on variables such as voltage, temperature, and surface interaction, bridging experimental findings with computational predictions.

While bifunctional electrocatalysts offer the appealing prospect of integrating HER and OER activity into a single material, several inherent challenges limit their practical application. One key limitation is the compromised performance for each half‐reaction; materials designed to operate under both reductive and oxidative conditions rarely deliver optimal activity for HER or OER individually, often resulting in elevated overpotentials or lower current densities. Moreover, distinct redox environments required for HER (reducing) and OER (oxidizing) pose significant stability challenges, as many materials are prone to structural degradation, including phase transformations, surface leaching, or morphological collapse during extended operation. Achieving bifunctionality also demands intricate material engineering, which complicates synthesis, reduces reproducibility, and presents barriers to scale‐up. The range of truly bifunctional materials is inherently limited, as most known catalysts exhibit asymmetrical activity toward either HER or OER, making material selection nontrivial. Finally, mismatched kinetics between the two reactions can lead to energy inefficiencies in overall water splitting systems, necessitating greater input energy compared to tandem systems using reaction‐specific catalysts. These factors underscore the importance of a balanced design approach and exploration of hybrid strategies that combine complementary materials or functional domains.

For industrial water‐splitting purposes, high overall efficiencies demand robust catalysts that can bear high‐voltage‐induced polarization; indeed, OER is crucially the most challenging amongst those, since it demands sustainability and rapid reaction rates with efficient control over gas generation. Again, the stability of these materials would have to be quite intensely investigated, including identification of degradation pathways and any eventual conversion products upon degradation. The integration of *in‐situ* monitoring with theoretical calculations will become a crucial factor in optimizing hybrid materials for commercial use, enhancing the performance, durability, and scalability of electrocatalytic applications.

Research conducted for electrocatalysis employing a comprehensive testing methodology includes material studies at varied overpotential values and onset potential analyses, as well as under diverse operational conditions with an estimate of the stability of the respective material. Thereby, the development of universally agreed‐upon standard conditions is important while comparing the ability of an electrocatalyst to work effectively and thus analysing it. These parameters include Faradaic efficiency, overpotential, Tafel slope, **J,** and mass loading for a more complete analysis of material performance for practical applications. Currently, most of the electrocatalytic tests are conducted in alkaline conditions. This limits the ability to assess the bifunctional performance of materials across various pH environments. Moreover, excess overpotential is required to evaluate material performance in neutral conditions, suggesting the necessity for catalysts optimized for operation near neutral pH. The development of advanced electrocatalysts that can work with great efficacy in freshwater and seawater is important for expanding their practical applications. This will not only allow for environmentally versatile catalytic processes but also make the materials more scalable for commercial electrochemical systems. Standardizing testing protocols and extending the focus of research to include diverse operating environments will significantly contribute to the optimization and adoption of electrocatalysts for real‐world applications.

The synthesis of catalytic materials should be performed in an eco‐friendly manner to avoid environmental damage. Most conventional synthesis techniques involve hazardous chemicals and energy‐intensive processes, posing risks to human health and the ecosystem. The synthesis process should therefore be optimized to use greener reagents, employ energy‐efficient protocols, and minimize waste generation. This would not only lead to alignment with environmental objectives but also enhance the practicality of scaling these materials for commercial applications, thus fostering a more responsible and sustainable approach to advancing catalytic technologies.

Ultimately, this review underscores that despite remarkable progress, overcoming the challenges of catalyst durability, scalability, and cost remains vital for the widespread adoption of water‐splitting technologies. Continued research into hybrid materials and electrolyte optimization holds promise for enhancing catalytic efficiency, setting the stage for the practical deployment of electrocatalysts in clean energy systems and advancing the transition toward a sustainable energy future.

## Conflict of Interest

The authors declare that they have no conflict of interest.
